# Supplementary material for: Grounding deep neural network predictions of human categorization behavior in understandable functional features: The case of face identity
Source: Patterns (N Y). 2021 Sep 10;2(10):100348. doi: 10.1016/j.patter.2021.100348 (PMC8515012; doi:10.1016/j.patter.2021.100348)
Supplement: Document S1. Figures S1–S20 [file mmc1.pdf]

Patterns, Volume *2*

Supplemental information

Grounding deep neural network predictions of human categorization behavior in understandable functional features: The case of face identity

Christoph Daube, Tian Xu, Jiayu Zhan, Andrew Webb, Robin A.A. Ince, Oliver G.B. Garrod, and Philippe G. Schyns

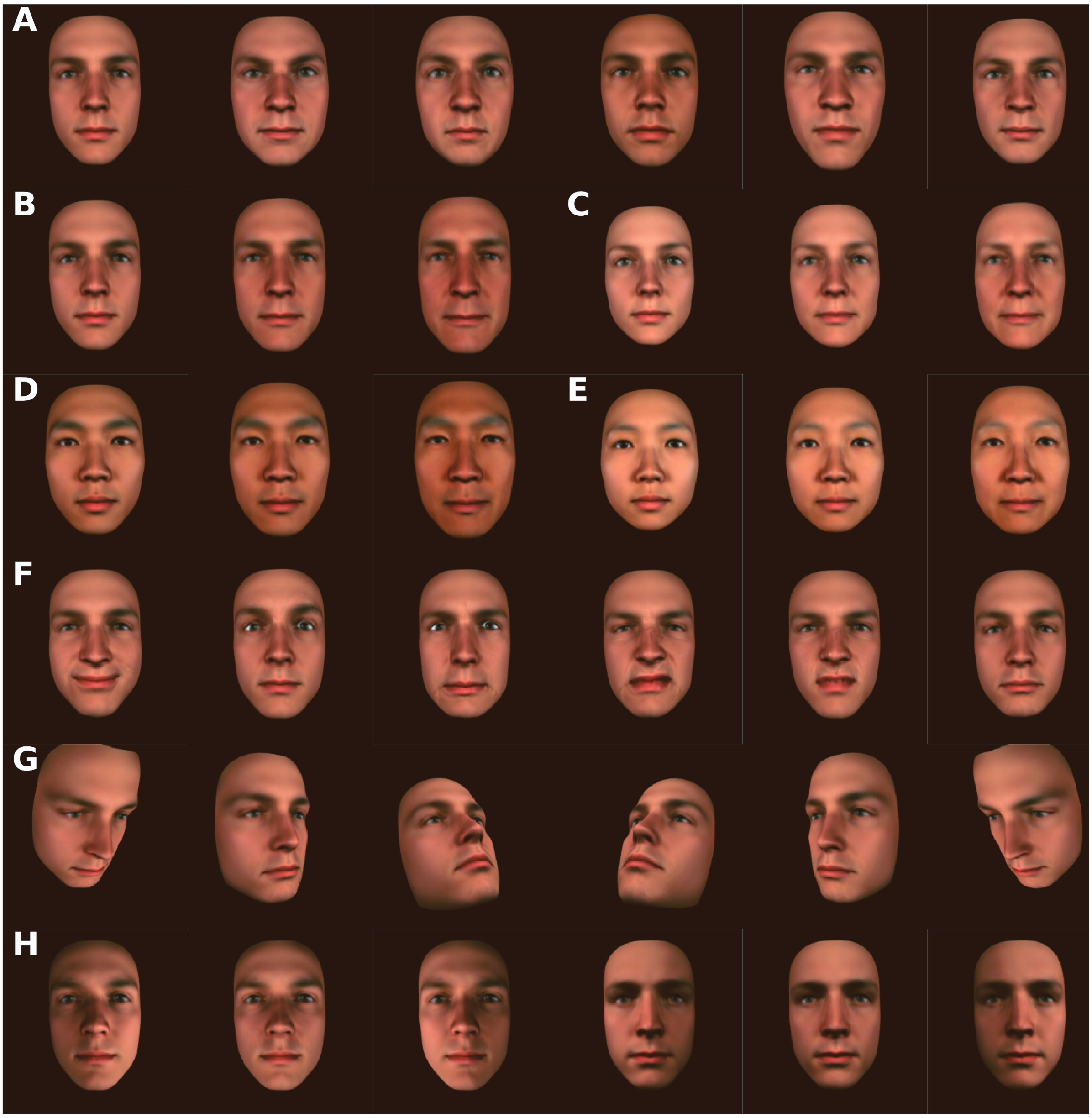

Figure S1: Demonstration of GMF variations used for training set of DNNs (related to Figure 2). **A** Six different example identities. **B** First identity from A rendered in three different ages. **C – E** Same as in B, but rendered with different sex and ethnicity. **F** First identity from A rendered with 6 additional expressions. **G** First identity from A rendered with different viewing angles. **H** First identity from A rendered with different lighting angles.

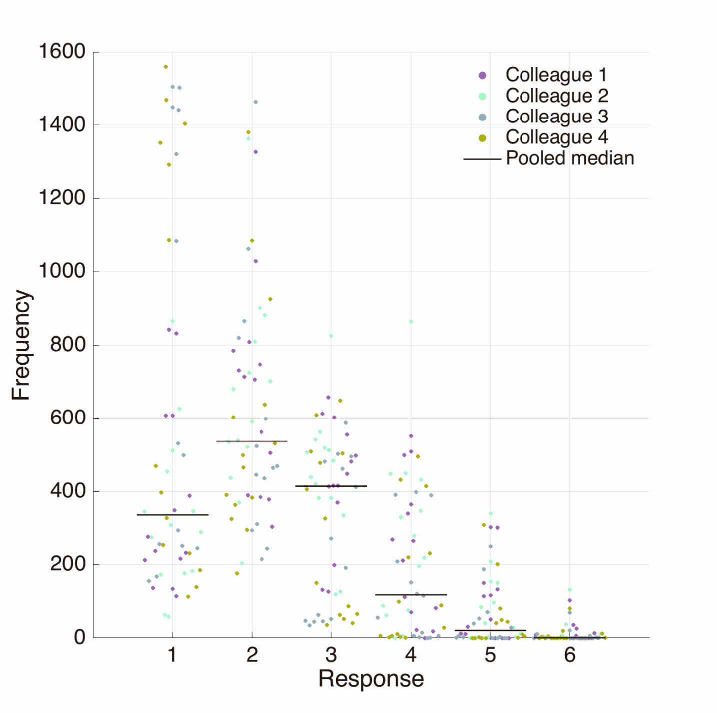

Figure S2: Distribution of rating responses in the human reverse correlation experiment (related to Figure 3).
1 codes for low similarity, 6 codes for highest similarity of stimulus to familiar target identity. Each data point represents the combination of one participant and one target familiar identity.

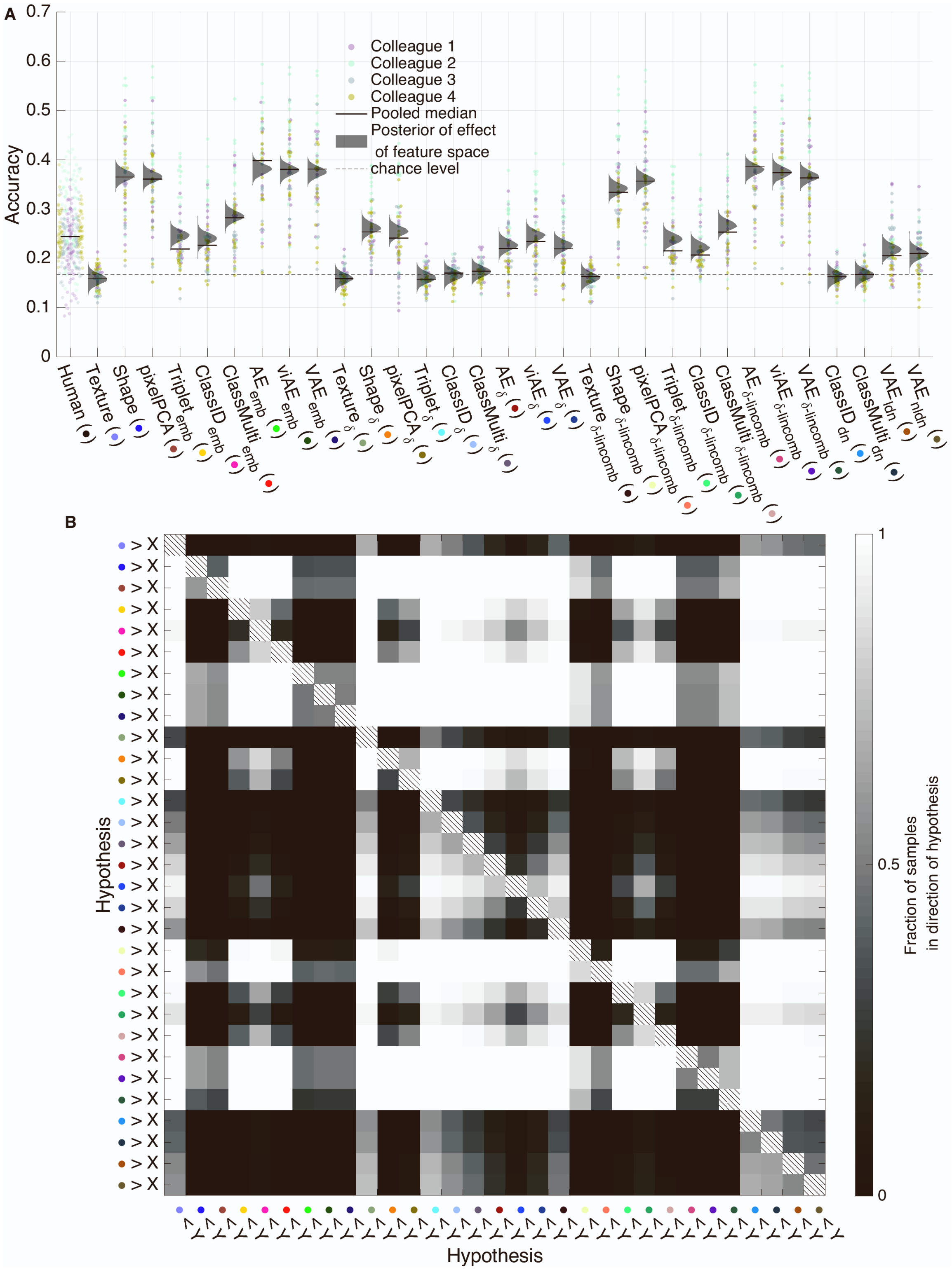

Figure S3: Accuracy of forward models in predicting choice behavior (related to Figure 3).
A Choice accuracy. On each trial, humans were presented with an array of 6 different random faces. They were asked to choose the one that most resembled the respective target colleague prior to reporting the perceived similarity on a 6-point rating scale. On each trial, the forward models "chose" the face of the array of 6 that had the highest rating among all faces of the array. The panel shows how well each model's choices matched the choices of the human participants. Pairwise matches of human participants with each other are displayed for reference. See figure S4 for explanation of the model shorthands. **B** Comparisons of the posterior distributions of the main effects for all forward models from Bayesian linear modeling of the raw performances. For each pair in the matrices, the color gradient reflects the fraction of samples of the system color coded on the y-axis that is larger than the system color coded on the x-axis. See x-axis labels for color legend.

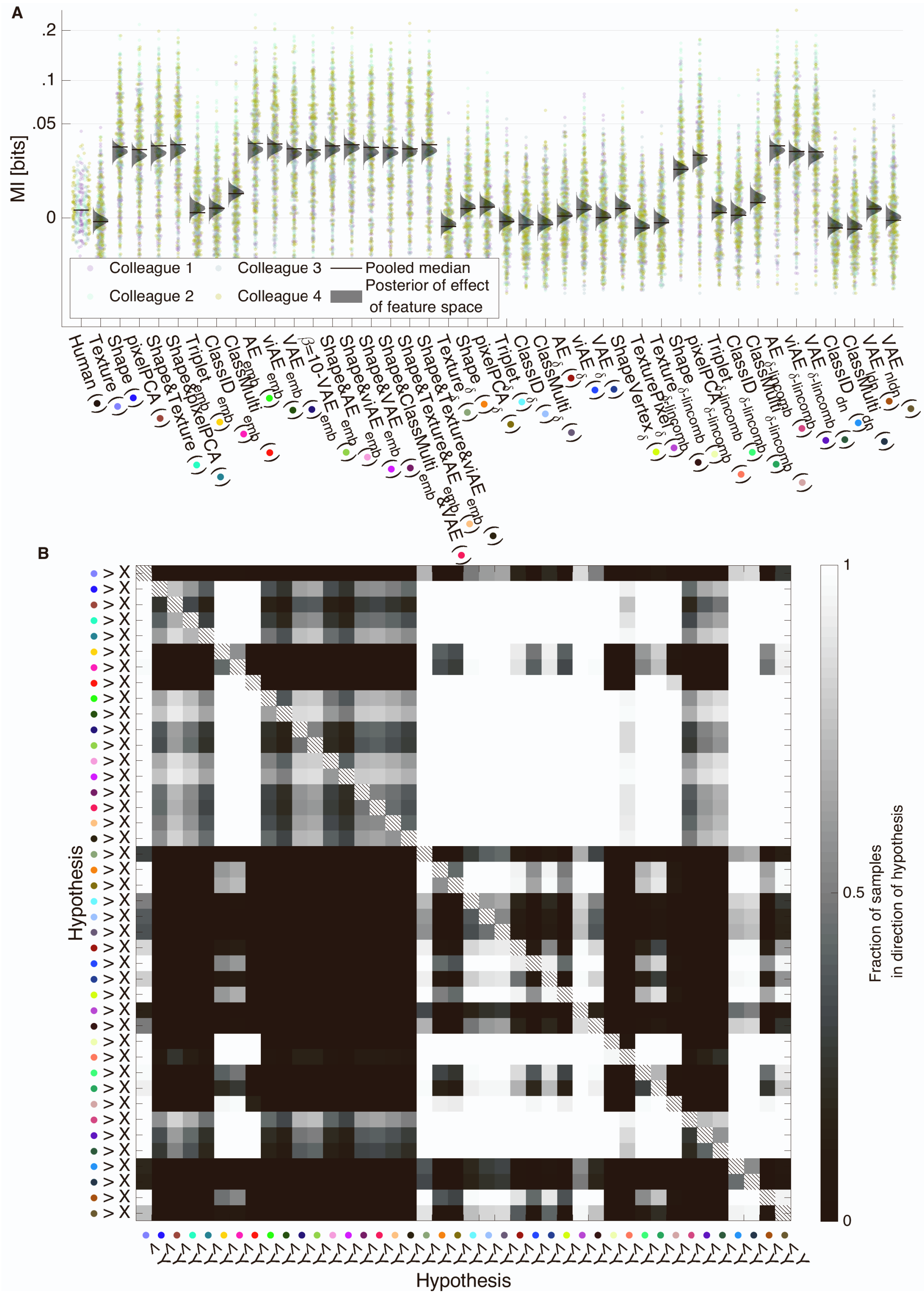

Figure S4: Bivariate evaluations of a larger set of encoding models (related to Figure 3).
A Mutual Information (MI) of observed behavior and test-set predictions from GMF features and various functions of DNN activations as well as human participants predicting other human participants (pairwise comparisons). Models include variational autoencoders ("VAE",[1]), VAEs with regularization ("β-VAE",[2]), euclidean distances of representations of the ground truth colleagues and the respective trials ("δ"), weighted euclidean distances ("δ-lincomb"), pre-softmax decision neuron activity ("logits") of the respective colleagues of ClassID and ClassMulti networks ("dn") as well as of ID classifiers trained on top of frozen VAE encoder networks (linear, "VAE$_{ldn}$", and with 2 rectified fully connected layers of 512 neurons ("VAE$_{nldn}$"). **B** Comparisons of the posterior distributions of the main effects for all systems from Bayesian linear modeling of the raw performances. For each pair in the matrices, the color gradient reflects the fraction of samples of the forward model color coded on the y-axis that is larger than the forward model color coded on the x-axis. See x-axis labels for color legend.

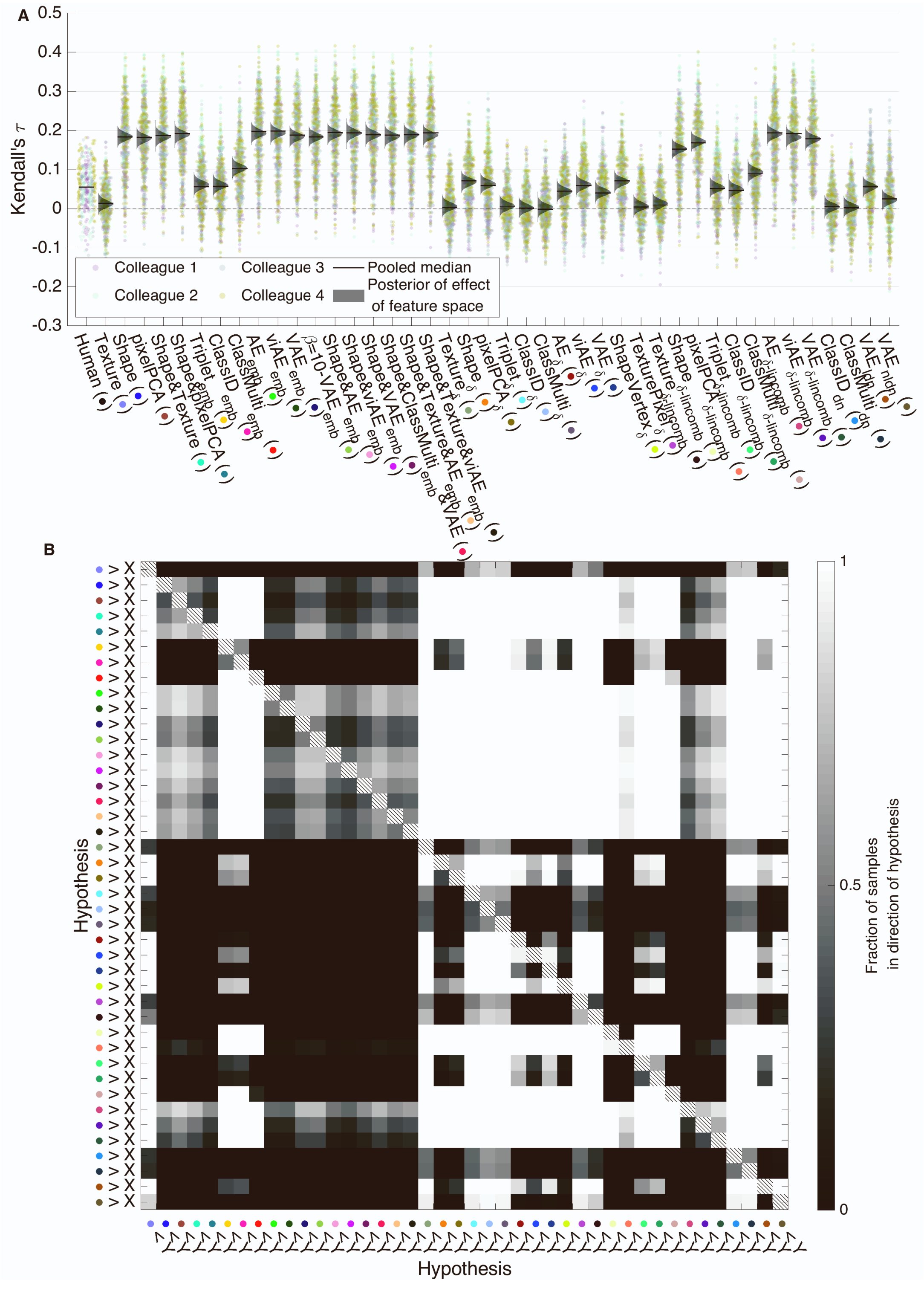

Figure S5: Bivariate evaluations of a larger set of encoding models (related to Figure 3).
A Kendall's τ of observed behavior and test-set predictions from GMF features and DNN activations as well as human participants predicting other human participants (pairwise comparisons). Except for the different metric, the analysis of this figure is identical to figure S4. See figure S4 for explanation of the model shorthands. **B** Comparisons of the posterior distributions of the main effects for all systems from Bayesian linear modeling of the raw performances. For each pair in the matrices, the color gradient reflects the fraction of samples of the forward model color coded on the y-axis that is larger than the forward model color coded on the x-axis. See x-axis labels for color legend.

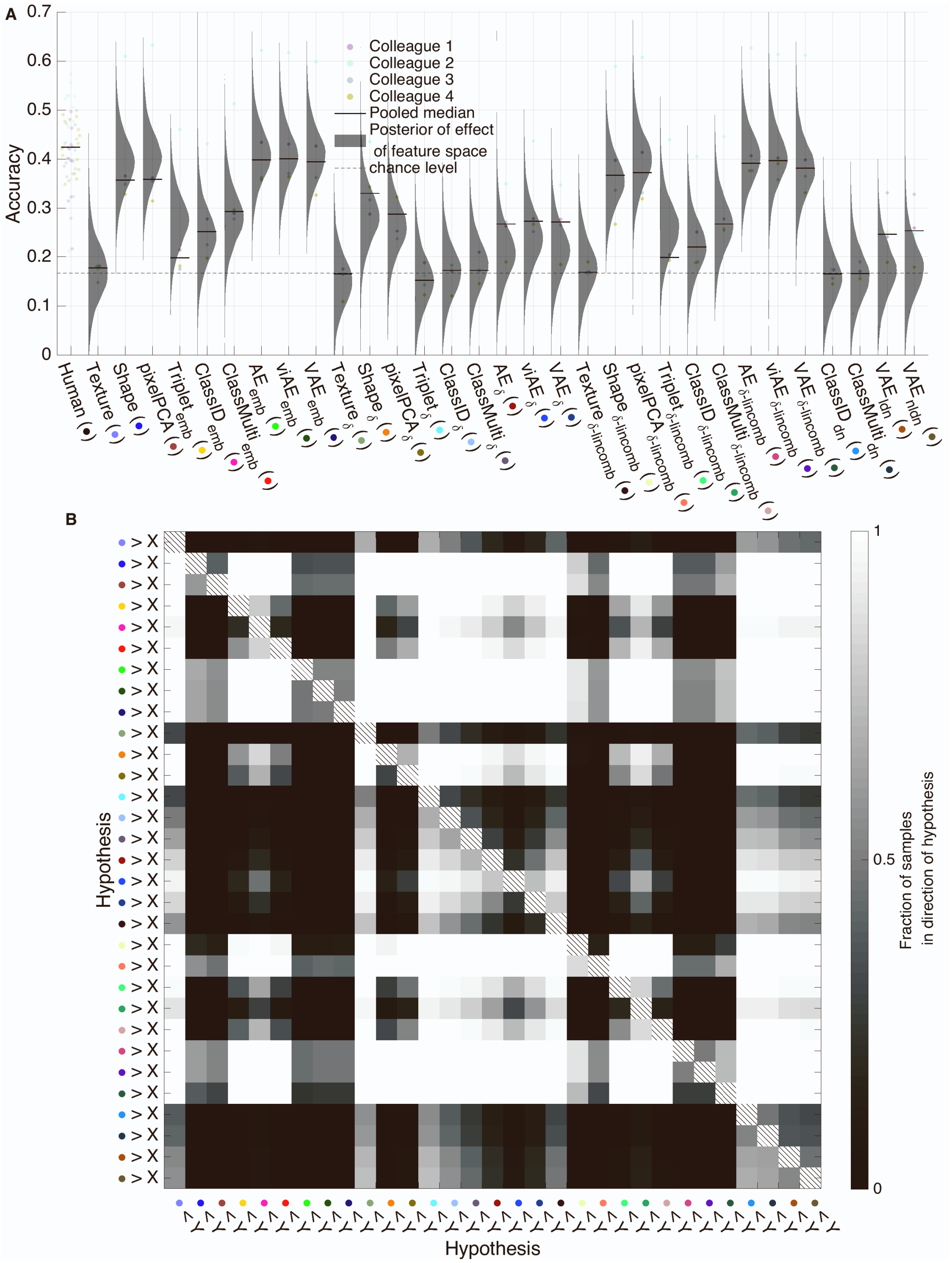

Figure S6: Accuracy of forward models in predicting choice behavior consensus across participants (related to Figure 3).
A Choice accuracy. Instead of predicting the behavior of individual human participants as in figure S3, here, for each panel of 6 faces per trial, the option chosen by the highest number of participants was used to represent the consensus across participants. See figure S4 for explanation of the model shorthands.
B Comparisons of the posterior distributions of the main effects for all systems from Bayesian linear modeling of the raw performances. For each pair in the matrices, the color gradient reflects the fraction of samples of the forward model color coded on the y-axis that is larger than the forward model color coded on the x-axis. See x-axis labels for color legend.

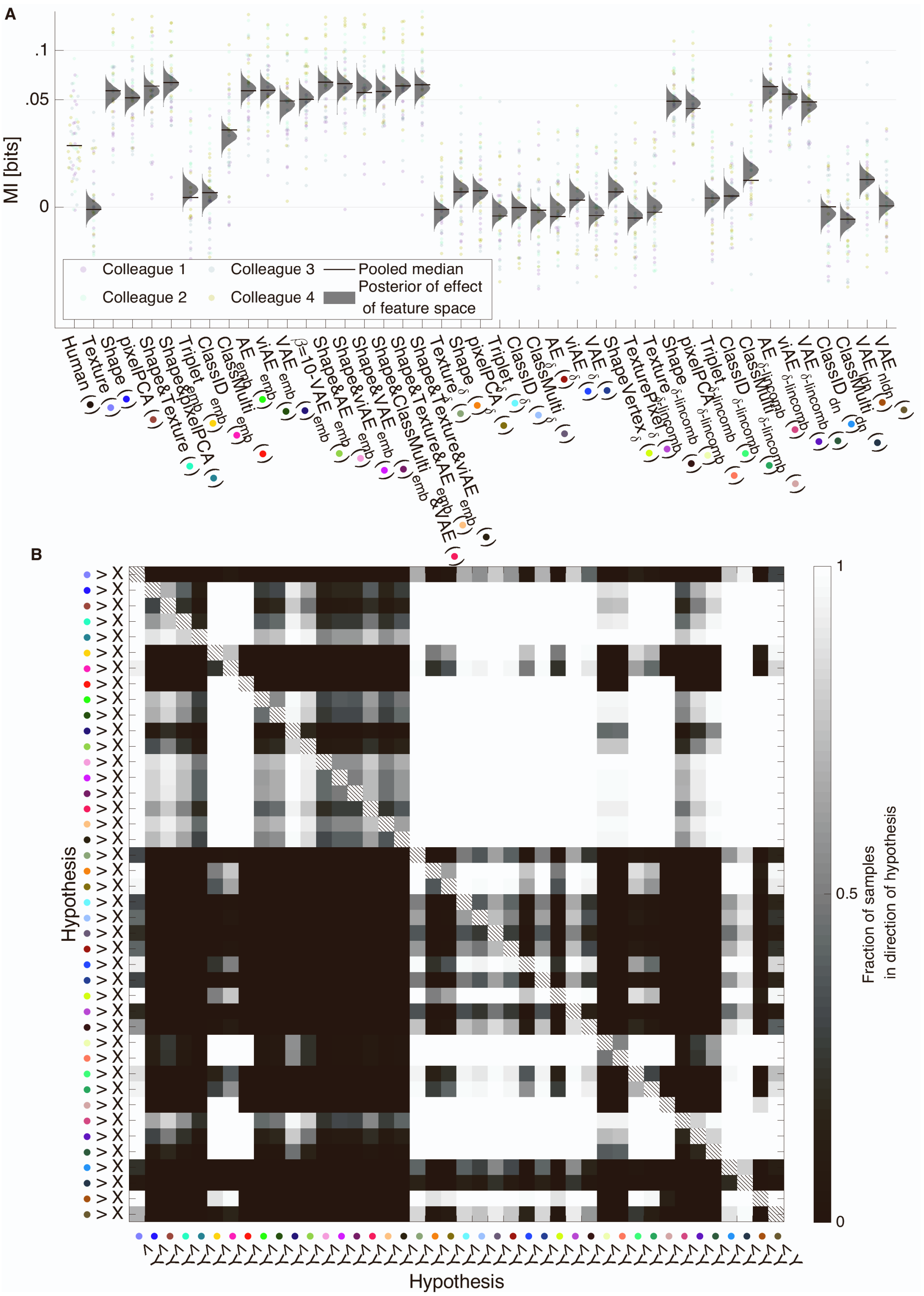

Figure S7: Bivariate evaluations of a larger set of encoding models on ratings averaged across participants (related to Figure 3).
A Mutual Information of averaged behavior and test-set predictions from GMF features and DNN activations as well as human participants predicting other human participants. Except for the different predictee, the analysis of this figure is identical to figure S4. See figure S4 for explanation of the model shorthands. **B** Comparisons of the posterior distributions of the main effects for all systems from Bayesian linear modeling of the raw performances. For each pair in the matrices, the color gradient reflects the fraction of samples of the forward model color coded on the y-axis that is larger than the forward model color coded on the x-axis. See x-axis labels for color legend.

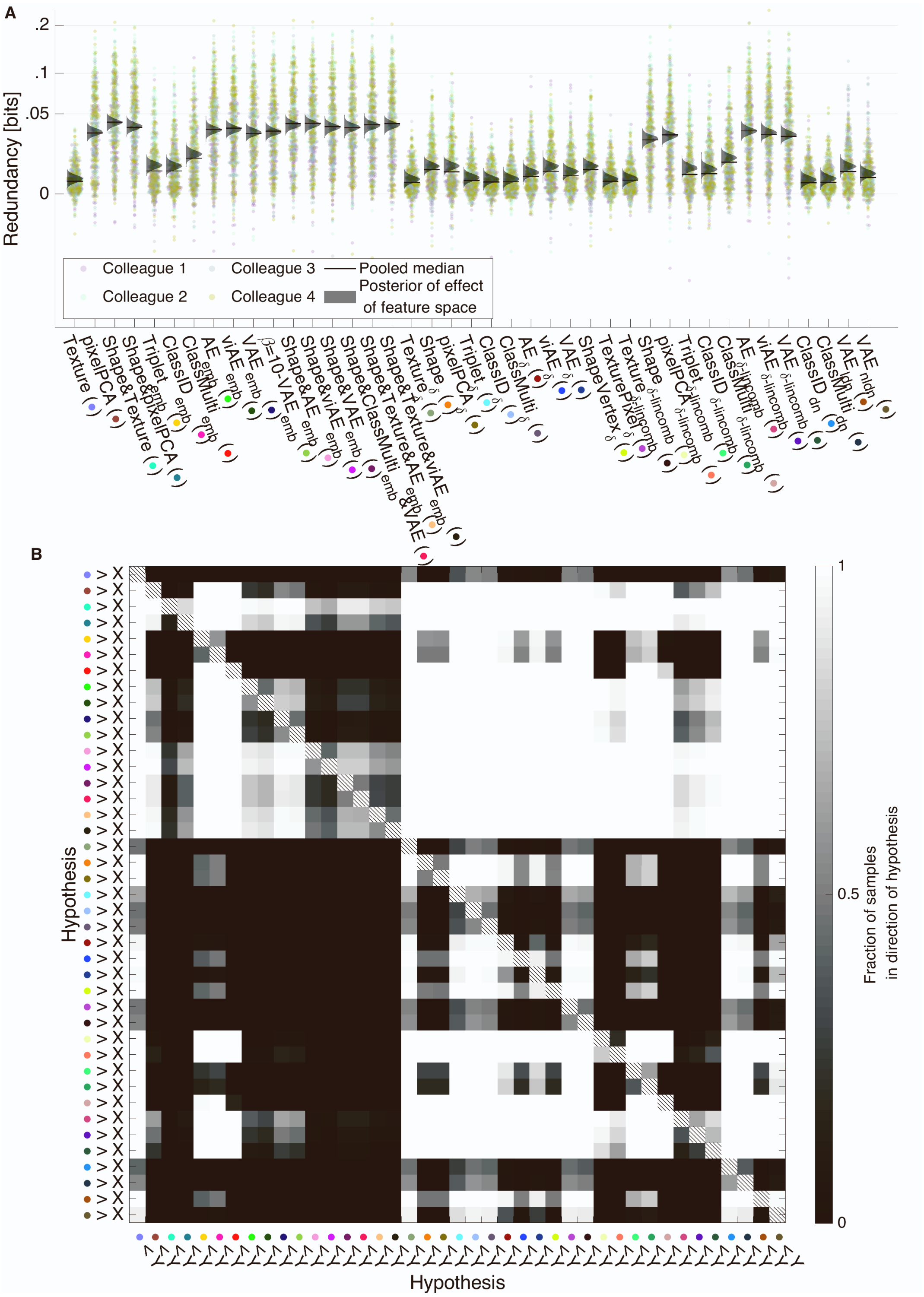

Figure S8: Redundancy with shape of a larger set of encoding models (related to Figure 3).
A Redundant information about human behavior that is shared between model predictions and GMF shape feature predictions. See figure S4 for explanation of the model shorthands. **B** Comparisons of the posterior distributions of the main effects for all systems from Bayesian linear modeling of the raw redundancies. For each pair in the matrices, the color gradient reflects the fraction of samples of the forward model color coded on the y-axis that is larger than the forward model color coded on the x-axis. See x-axis labels for color legend.

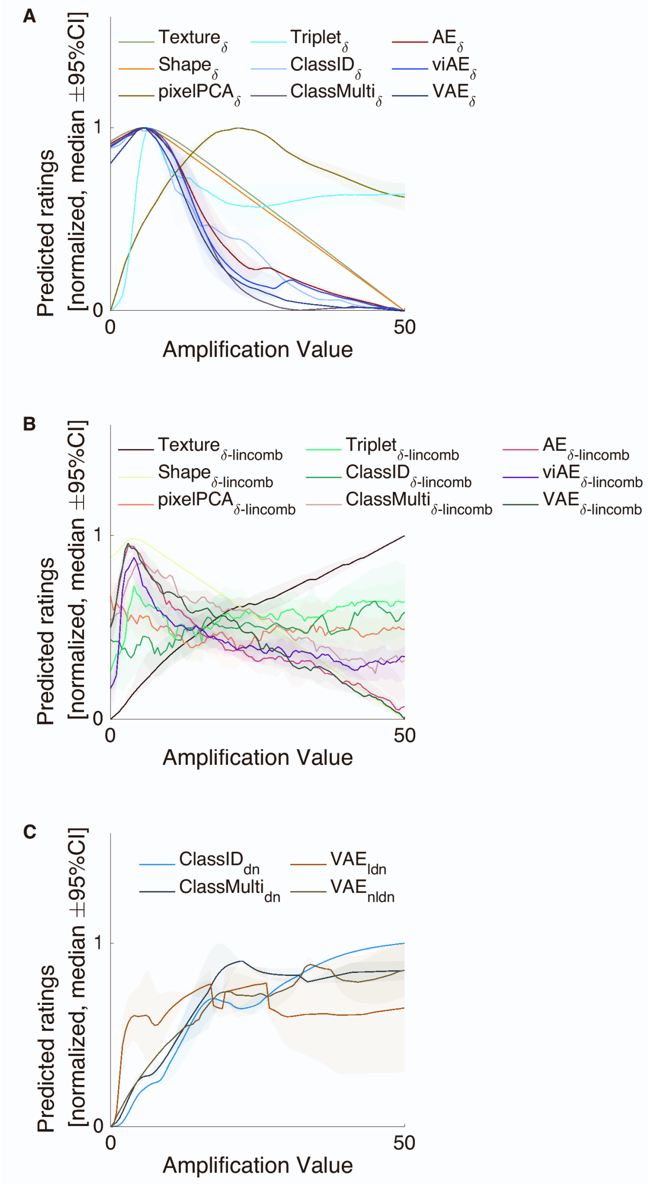

Figure S9: Amplification tuning responses of additional encoding models (related to Figure 4). **A** Amplification tuning responses of euclidean distances (“δ”) of templates amplified at different levels and ground truth representations of the target colleagues. Solid lines denote the pooled median across participants and target colleagues, shaded regions denote 95% (frequentist) confidence intervals bootstrapped using $10,000$ samples. **B** Same as in A, but showing amplification tuning responses of linearly weighted euclidean distances instead (“δ-lincomb”). **C** Same as in A, but showing amplification tuning responses of pre-softmax decision neuron activities (“logits”) of respective target colleagues instead (“dn”).

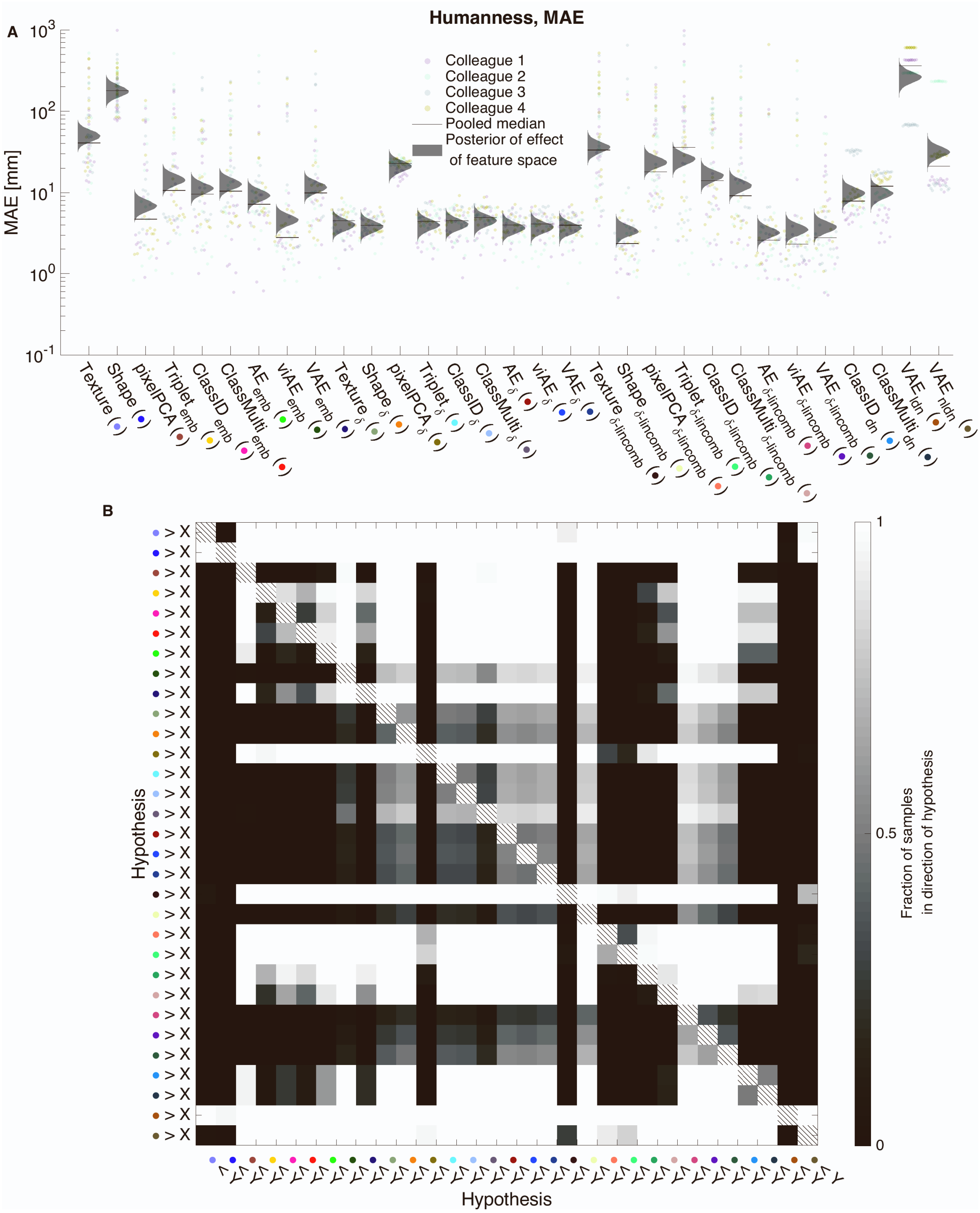

Figure S10: Evaluation of the mean absolute error between reverse-correlated faces of humans and reverse-correlated faces of models for a larger set of encoding models (related to Figure 4). **A** Mean absolute error (MAE, computed as the euclidean distances in 3D space averaged across vertices) of reverse correlated templates of the models and those of humans. See figure S4 for explanation of the model shorthands. **B** Comparisons of the posterior distributions of the main effects for all systems from Bayesian linear modeling of the raw performances. For each pair in the matrices, the color gradient reflects the fraction of samples of the forward model color coded on the y-axis that is larger than the forward model color coded on the x-axis. See x-axis labels for color legend.

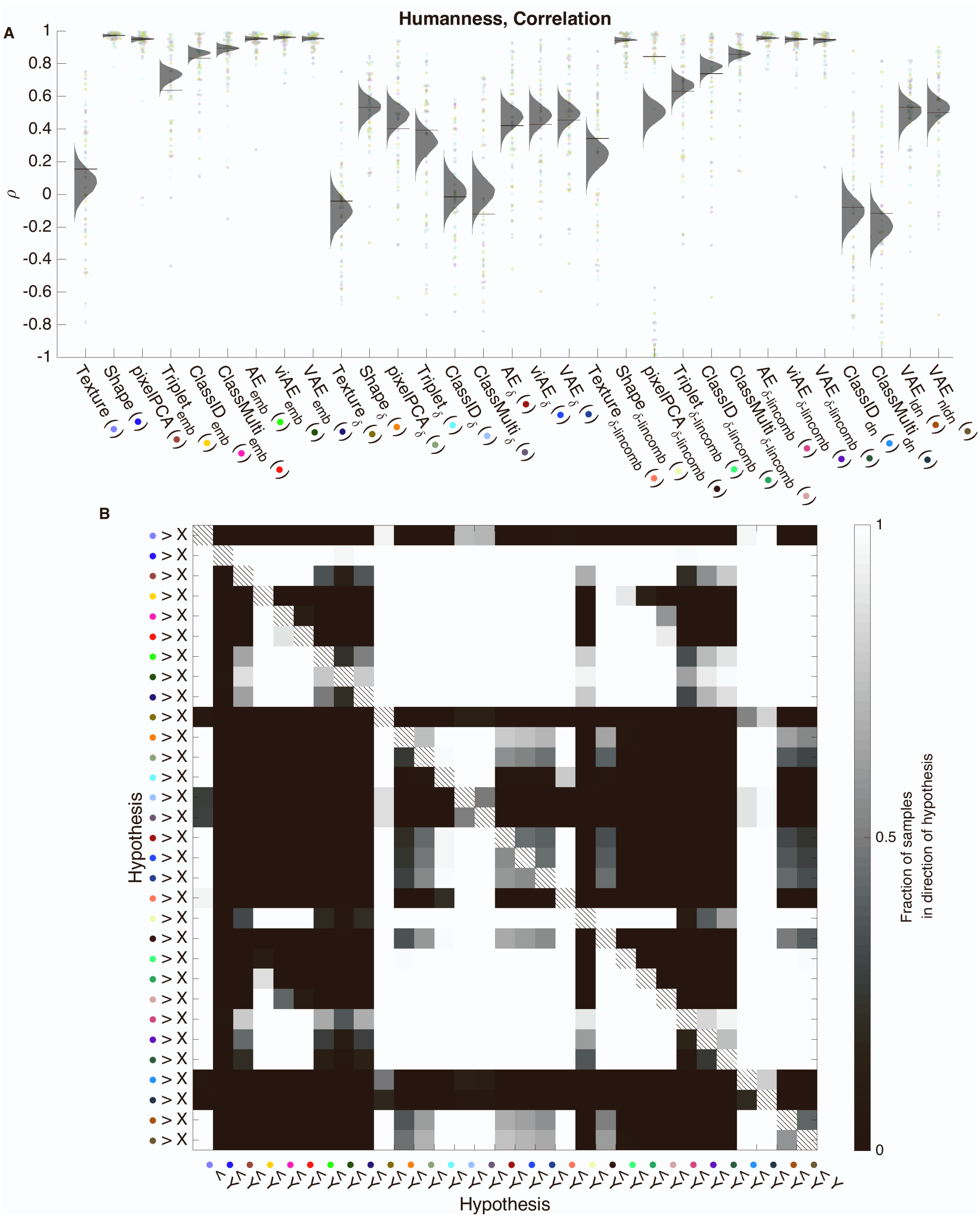

Figure S11: Evaluation of the Pearson correlation between reverse-correlated faces of humans and reverse-correlated faces of models for a larger set of encoding models (related to Figure 4). **A** Pearson correlation (computed with vectors of 3D vertices projected on a single inward-outward dimension) of reverse correlated templates of the models and those of humans. See figure S4 for explanation of the model shorthands. **B** Comparisons of the posterior distributions of the main effects for all systems from Bayesian linear modeling of the raw performances. For each pair in the matrices, the color gradient reflects the fraction of samples of the forward model color coded on the y-axis that is larger than the forward model color coded on the x-axis. See x-axis labels for color legend.

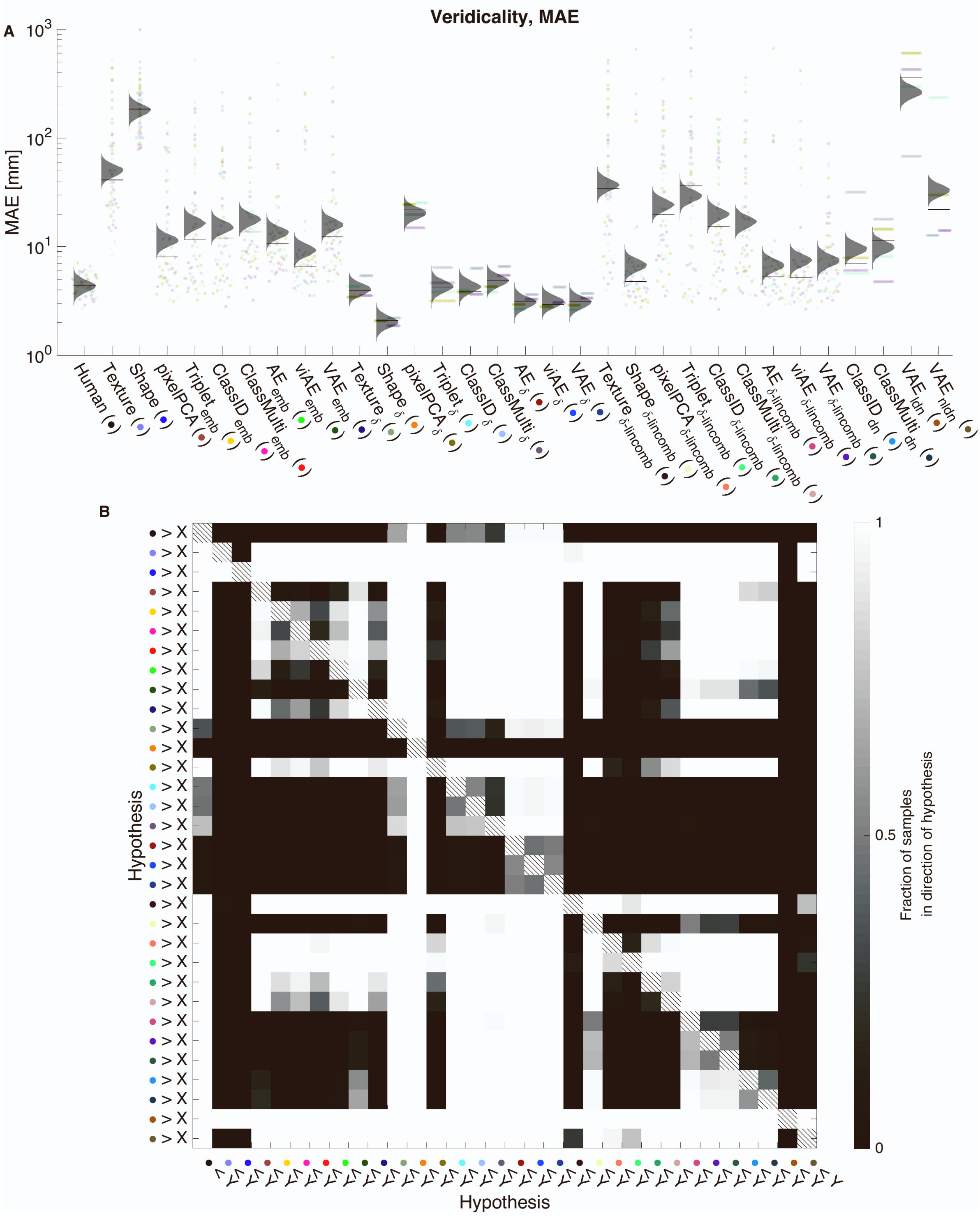

Figure S12: Evaluation of the mean absolute Error between reverse-correlated faces of humans and models and the ground truth face shapes for a larger set of encoding models (related to Figure 4).

A Mean absolute error (MAE, computed as the euclidean distances in 3D space averaged across vertices) of reverse correlated templates of the models and ground truth 3D shape of the target colleagues as captured with a 3D camera array. See figure S4 for explanation of the model shorthands. **B** Comparisons of the posterior distributions of the main effects for all systems from Bayesian linear modeling of the raw performances. For each pair in the matrices, the color gradient reflects the fraction of samples of the forward model color coded on the y-axis that is larger than the forward model color coded on the x-axis. See x-axis labels for color legend.

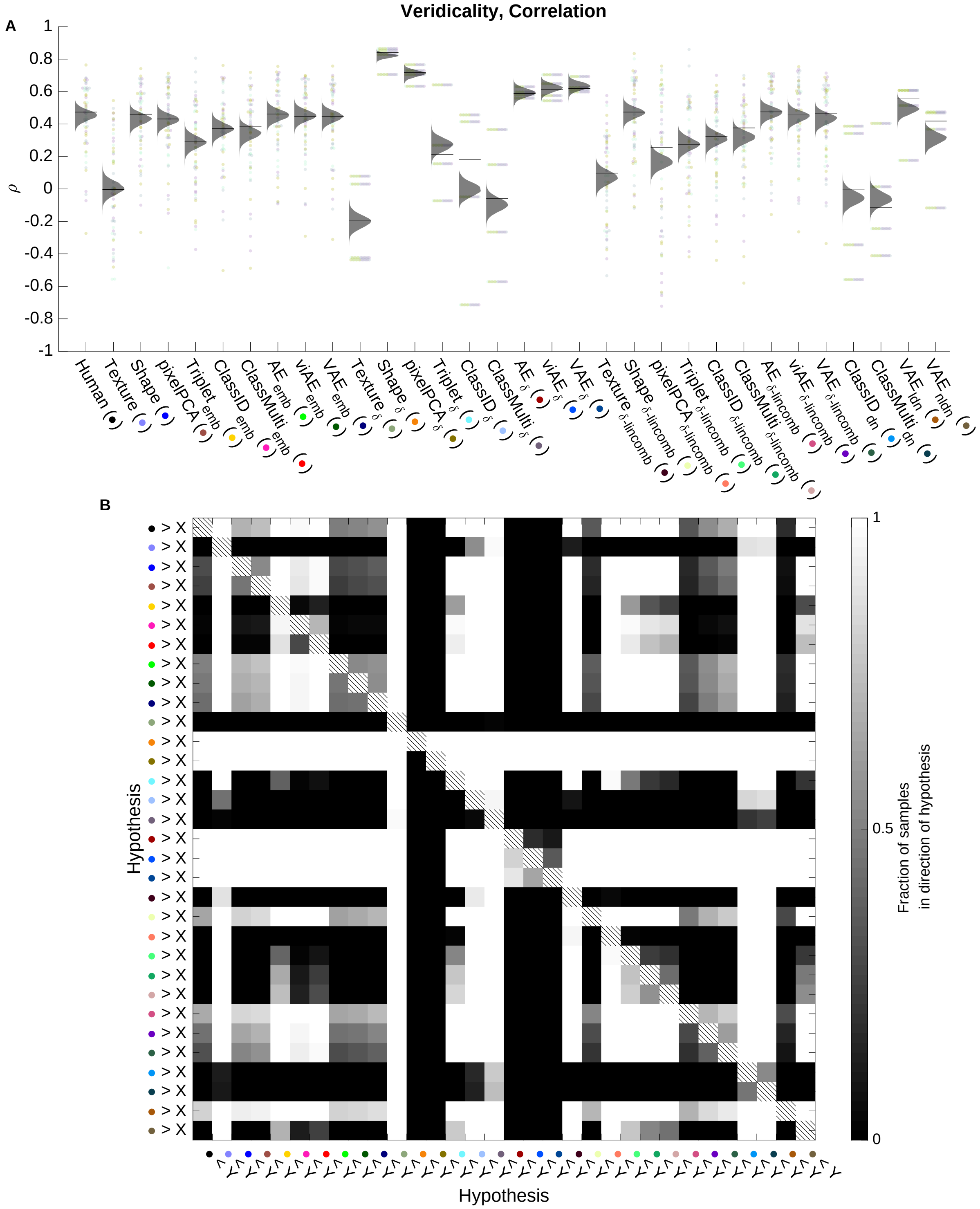

Figure S13: Evaluation of the Pearson correlation between reverse-correlated faces of humans and models and the ground truth face shapes for a larger set of encoding models (related to Figure 4).
A Pearson correlation (computed with vectors of 3D vertices projected on a single inward-outward dimension) of reverse correlated templates of the models and those of humans. See figure S4 for explanation of the model shorthands. **B** Comparisons of the posterior distributions of the main effects for all systems from Bayesian linear modeling of the raw performances. For each pair in the matrices, the color gradient reflects the fraction of samples of the forward model color coded on the y-axis that is larger than the forward model color coded on the x-axis. See x-axis labels for color legend.

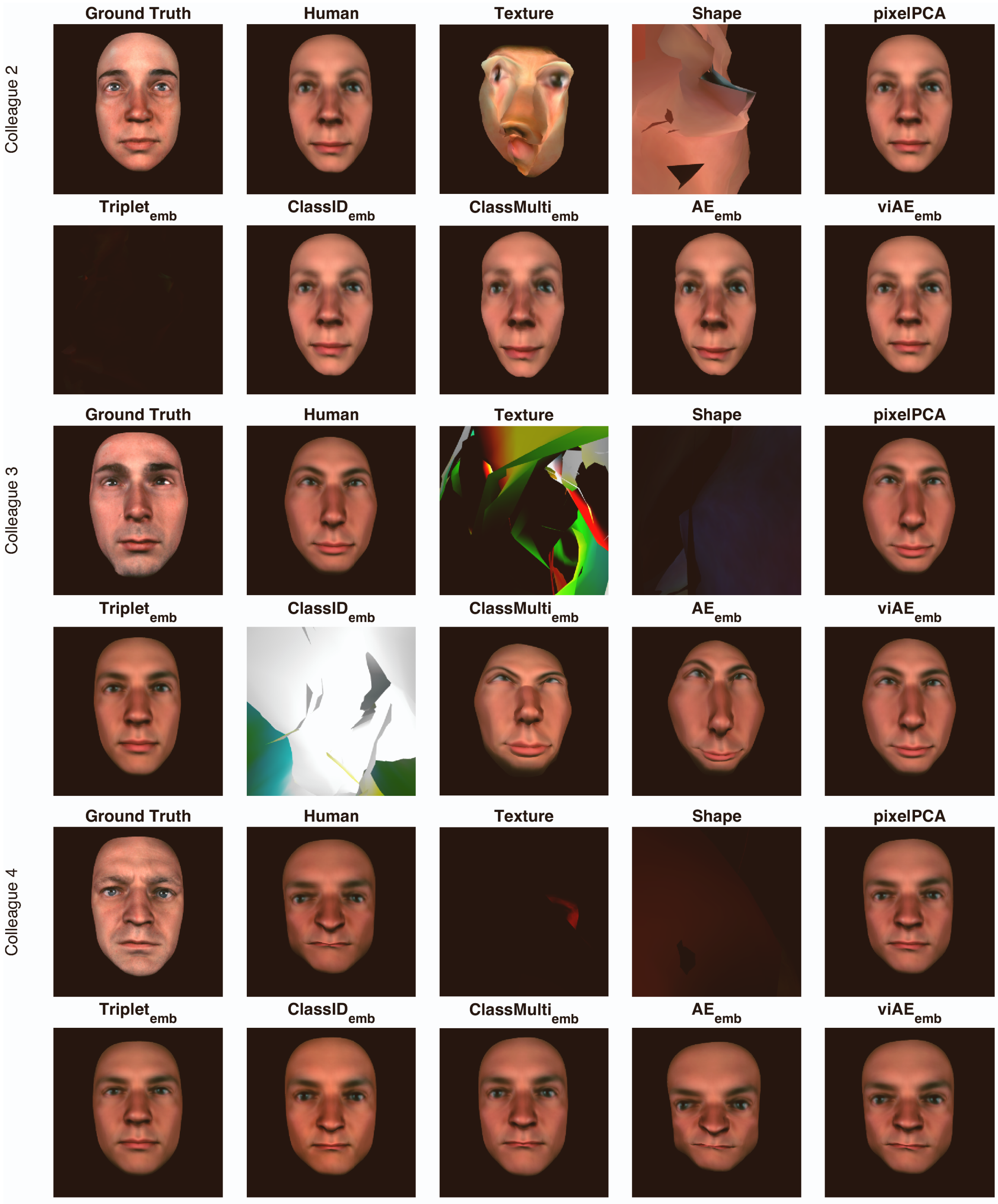

Figure S14: Renderings of reverse-correlated templates of the three remaining colleagues of exemplary participant (related to Figure 4).
Comparison of rendered faces for one exemplary target colleague. Top left panel in each block of two rows shows ground truth face of one target colleague as captured with a 3D camera array. Following panels show reconstructions of the face features from human observed and predicted behavior for one typical participant (i.e. closest to the pooled group medians shown in Figure 4D).

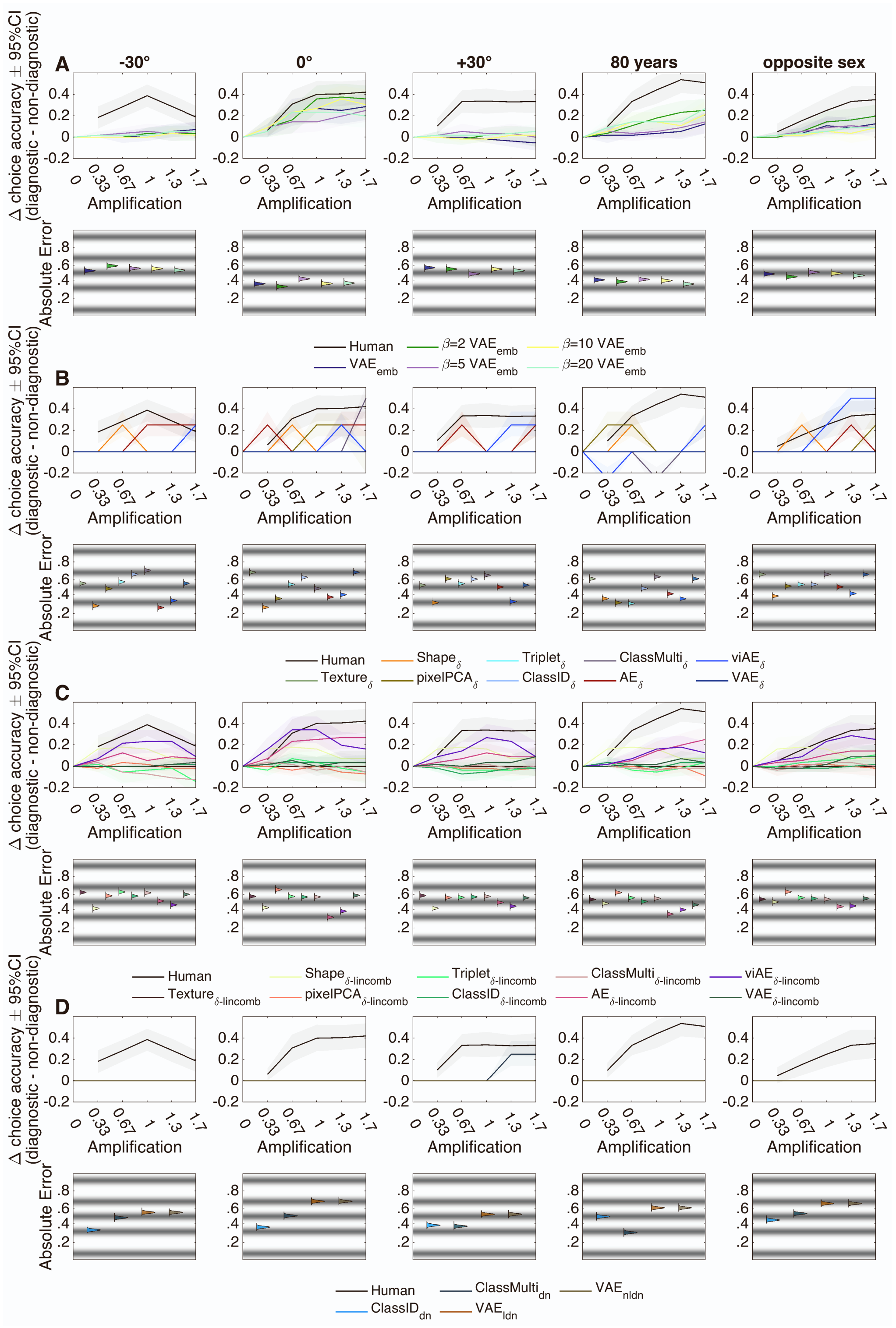

Figure S15: Generalization testing of a larger set of encoding models (related to Figure 5).
A Generalization testing for VAE models with various degrees of regularization. None yield a factorization of the latent spaces that disentangles viewing angle from other factors. Top row shows difference of choice accuracy between the diagnostic and non-diagnostic conditions. Positive values denote a higher accuracy when diagnostic features were amplified. Bottom row shows posterior distributions of main effects of feature spaces when modeling absolute error vs humans with Bayesian linear model. Grey bandings denote density estimates of thresholds separating the five different error values possible (human accuracies are averaged across five ratings of the same item). **B** – **D** show the same as in **A**, but for different forward models. See figure S4 for explanation of the model shorthands.

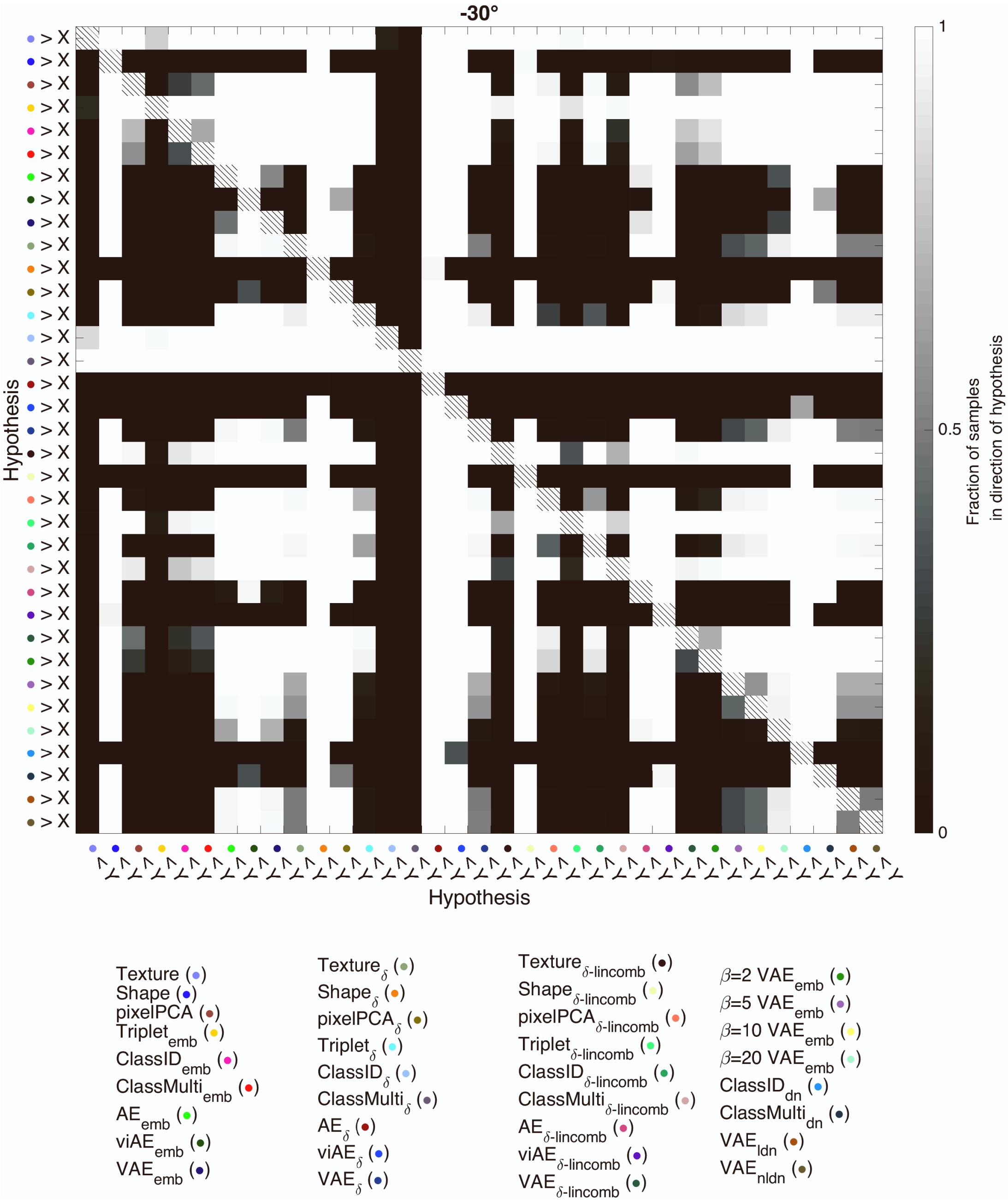

Figure S16: Comparison of posterior distributions for larger set of forward models in -30° viewing angle generalization (related to Figure 5).

Comparison of the posterior distributions of the leftmost column in figure S15. For each pair in the matrices, the color gradient reflects the fraction of samples of the feature space color coded on the y-axis that is larger than the predictor space color coded on the x-axis. See figure S4 for explanation of the model shorthands.

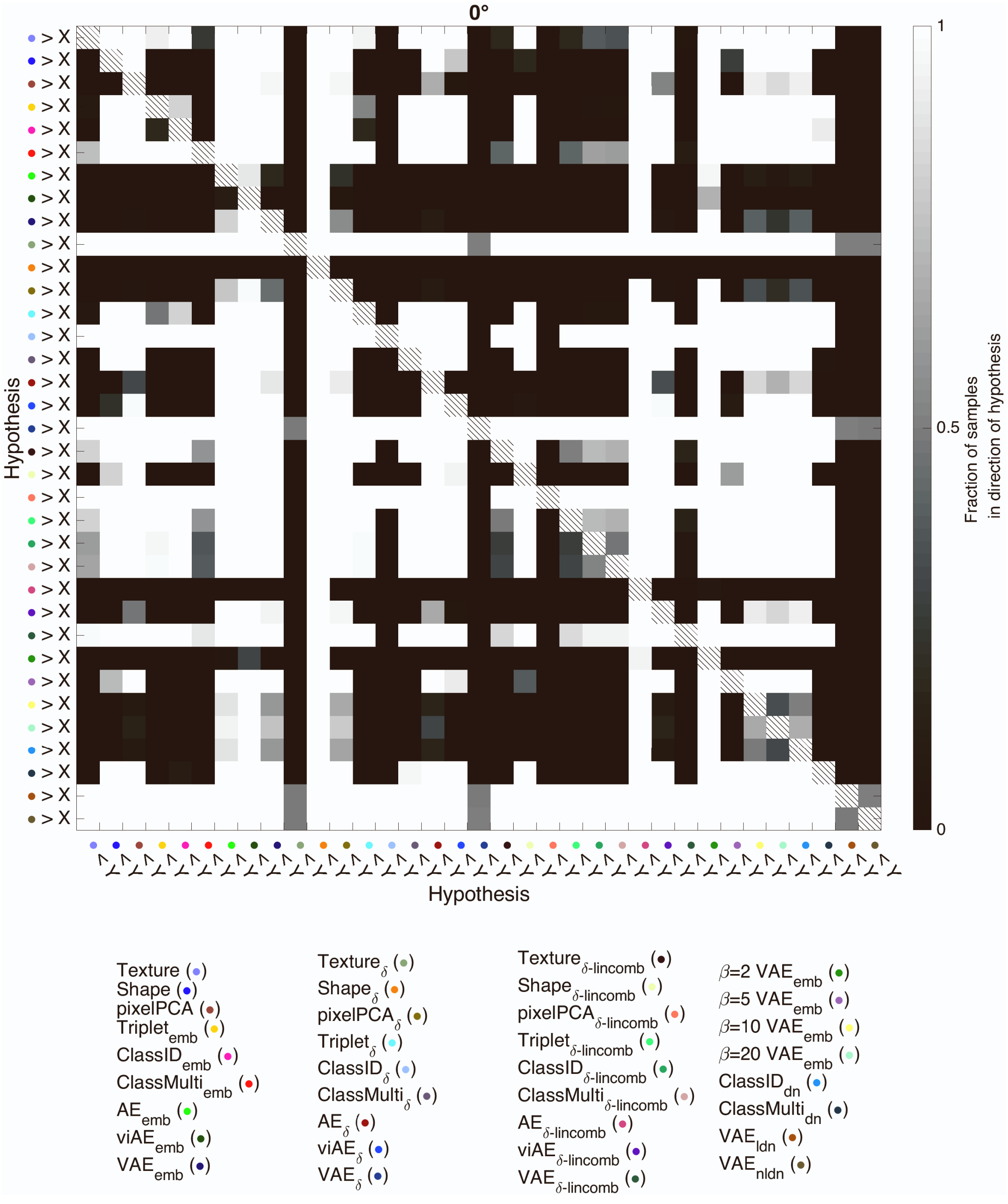

Figure S17: Comparison of posterior distributions for larger set of forward models in 0° viewing angle generalization (related to Figure 5).
Comparison of the posterior distributions of the second column in figure S15. For each pair in the matrices, the color gradient reflects the fraction of samples of the feature space color coded on the y-axis that is larger than the predictor space color coded on the x-axis. See figure S4 for explanation of the model shorthands.

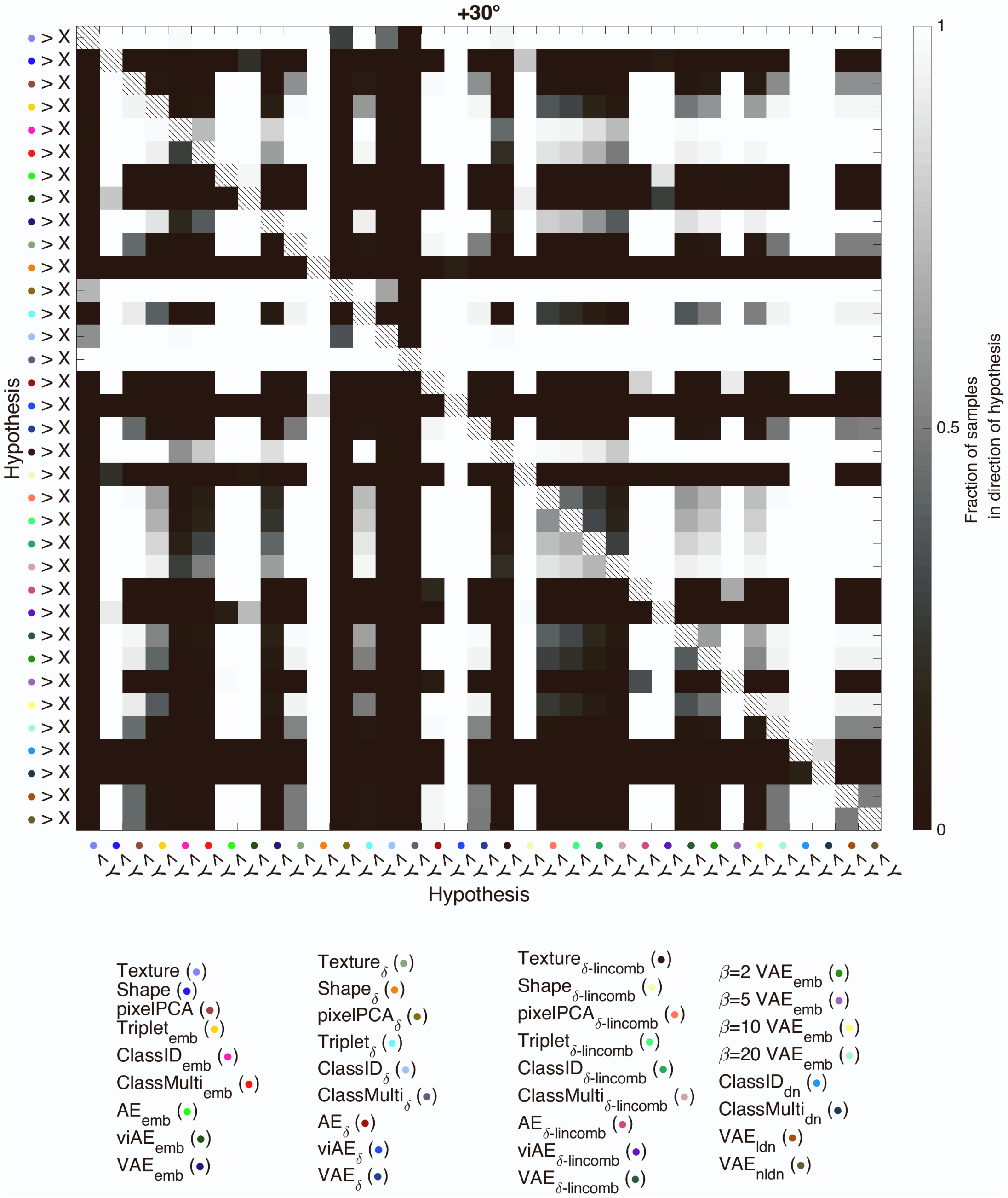

Figure S18: Comparison of posterior distributions for larger set of forward models in +30° viewing angle generalization (related to Figure 5).
Comparison of the posterior distributions of the middle column in figure S15. For each pair in the matrices, the color gradient reflects the fraction of samples of the feature space color coded on the y-axis that is larger than the predictor space color coded on the x-axis. See figure S4 for explanation of the model shorthands.

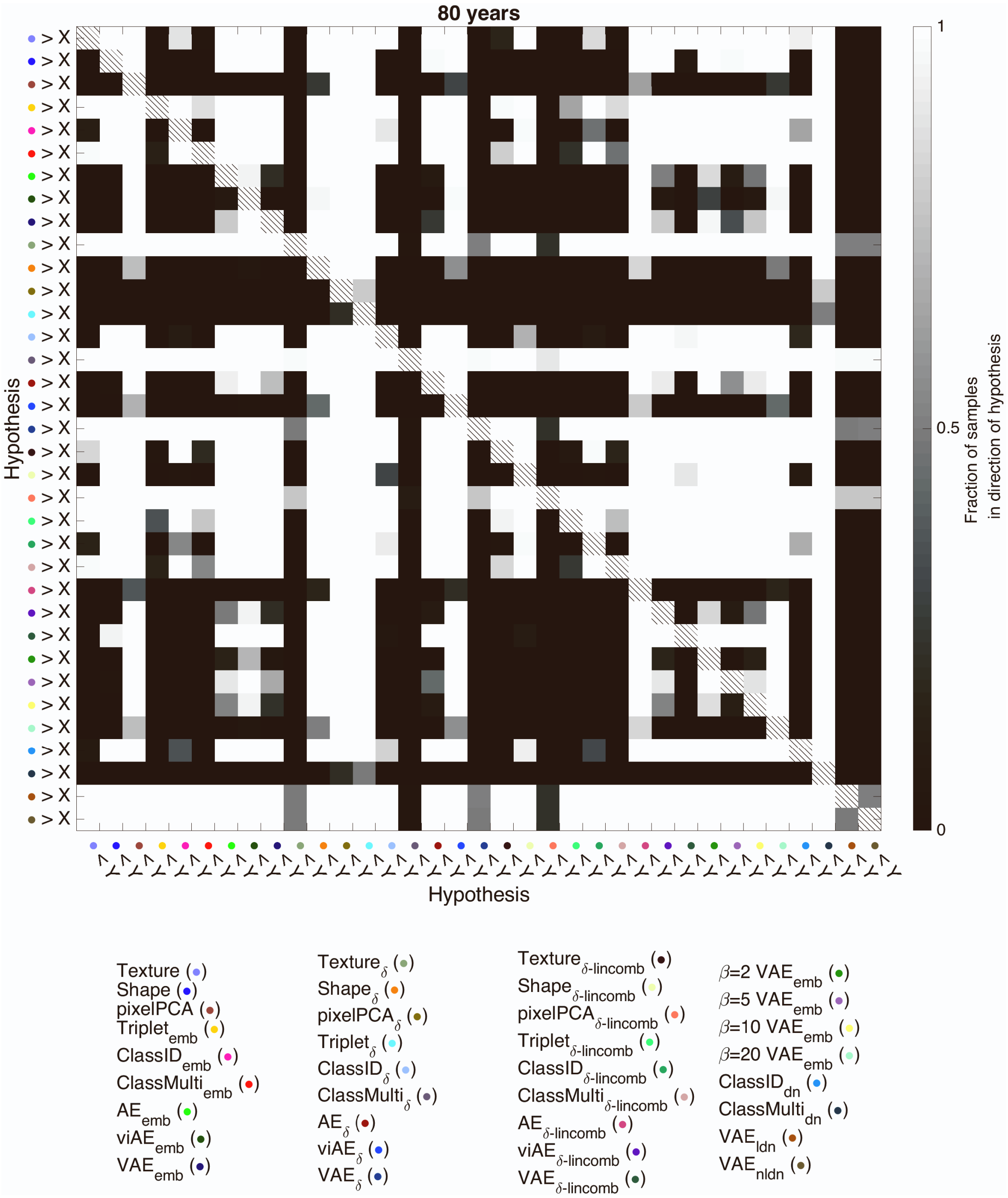

Figure S19: Comparison of posterior distributions for larger set of forward models in 80 years generalization (related to Figure 5).
Comparison of the posterior distributions of the fourth column in figure S15. For each pair in the matrices, the color gradient reflects the fraction of samples of the feature space color coded on the y-axis that is larger than the predictor space color coded on the x-axis. See figure S4 for explanation of the model shorthands.

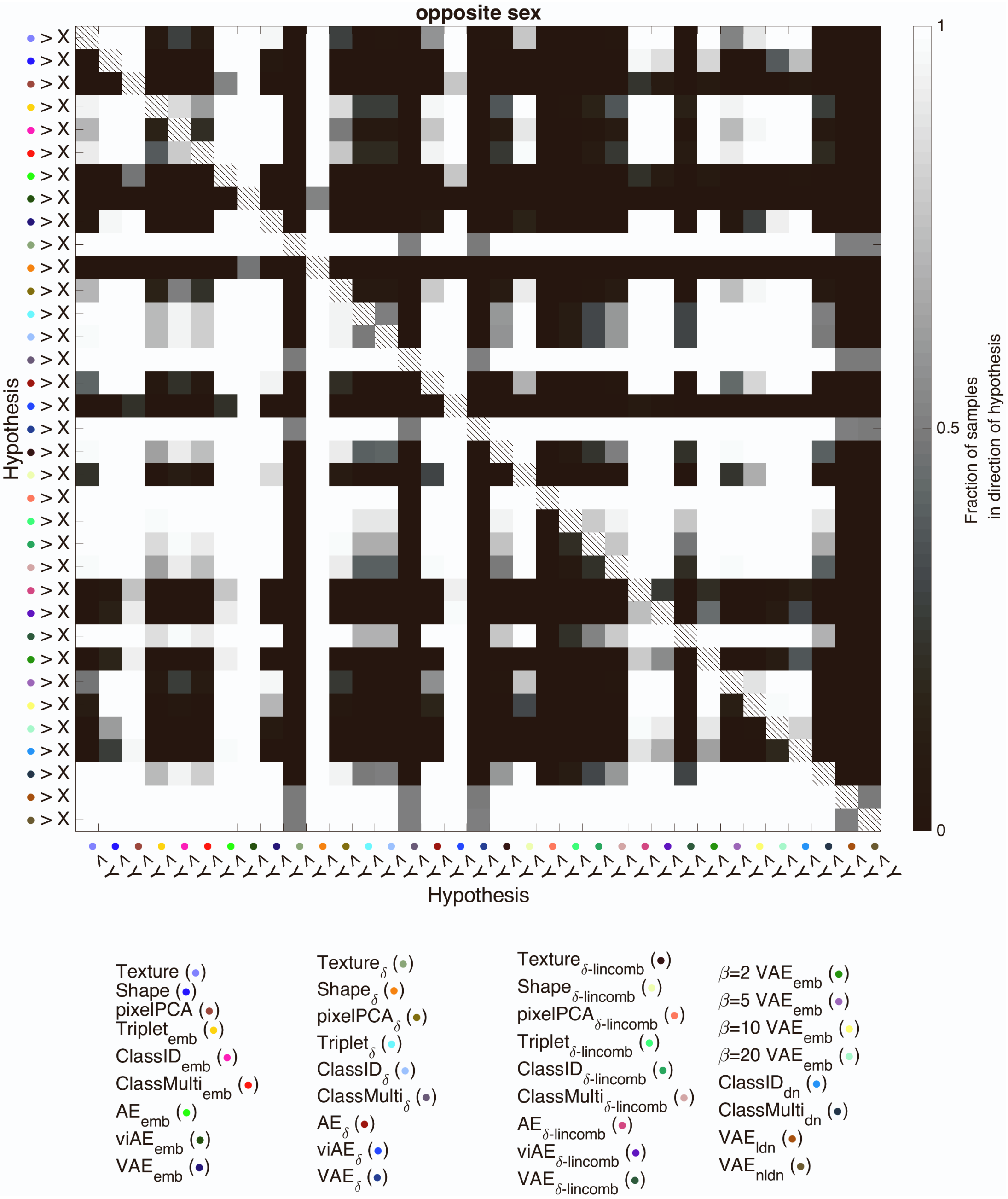

Figure S20: Comparison of posterior distributions for larger set of forward models in opposite sex generalization (related to Figure 5).
Comparison of the posterior distributions of the rightmost column in figure S15. For each pair in the matrices, the color gradient reflects the fraction of samples of the feature space color coded on the y-axis that is larger than the predictor space color coded on the x-axis. See figure S4 for explanation of the model shorthands.

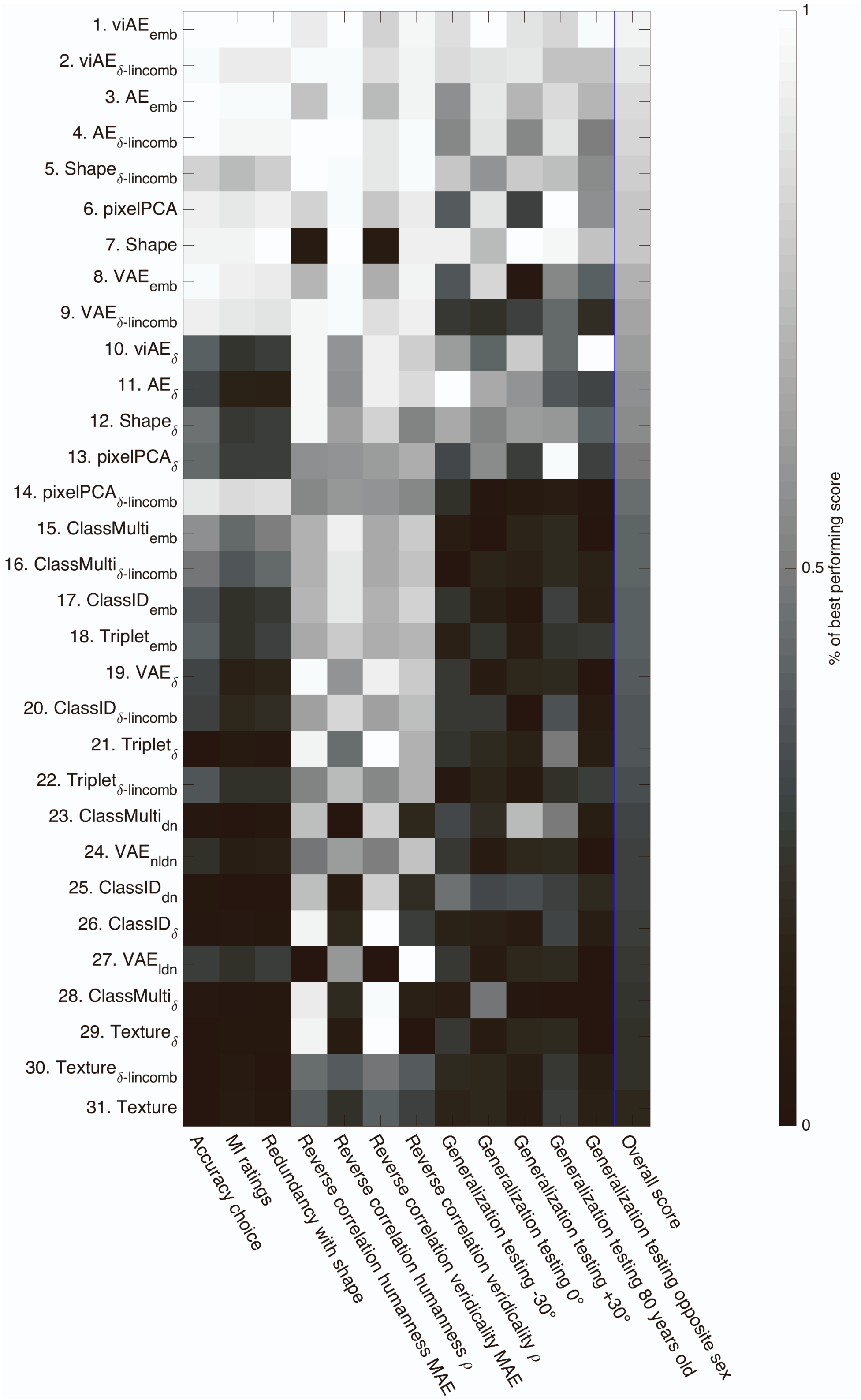

Figure S21: Ranking of extended set of models (related to Figures 3, 5, 6).
We integrated the results of the models in all comparisons except for the re-prediction analysis reported in Figure 4 (which is only applicable to linear combination forward models). Redundancy of the shape model with itself is not computable and was thus manually set to the best possible score. Scores in veridicality of reverse correlation were defined as the absolute difference to the veridicality achieved by humans. Scores in generalization testing (absolute error to human behavior) were additionally penalized for a low delta in accuracy of diagnostic and non-diagnostic stimuli. Performances of models (maxima a posteriori of Bayesian linear models) were normalized within comparisons to range from 0 (worst considered model) to 1 (best considered model). Scores were summed across comparisons and divided by the number of comparisons for the overall score. See figure S4 for explanation of the model shorthands.

Supplemental References

1. Kingma DP, Welling M. Auto-Encoding Variational Bayes. arXiv:13126114 [cs, stat]. 2014 May;ArXiv: 1312.6114. Available from: http://arxiv.org/abs/1312.6114.

2. Higgins I, Matthey L, Pal A, Burgess C, Glorot X, Botvinick M, et al. beta-VAE: Learning Basic Visual Concepts with a Constrained Variational Framework. ICLR. 2016 Nov;Available from: https://openreview.net/forum?id=Sy2fzU9gl.
