# Supplementary material for: Grounding deep neural network predictions of human categorization behavior in understandable functional features: The case of face identity
Source: Patterns (N Y). 2021 Sep 10;2(10):100348. doi: 10.1016/j.patter.2021.100348 (PMC8515012; doi:10.1016/j.patter.2021.100348)
Supplement: Document S2. Article plus supplemental information [file mmc2.pdf]

Patterns

Article

Grounding deep neural network predictions of human categorization behavior in understandable functional features: The case of face identity

Graphical abstract

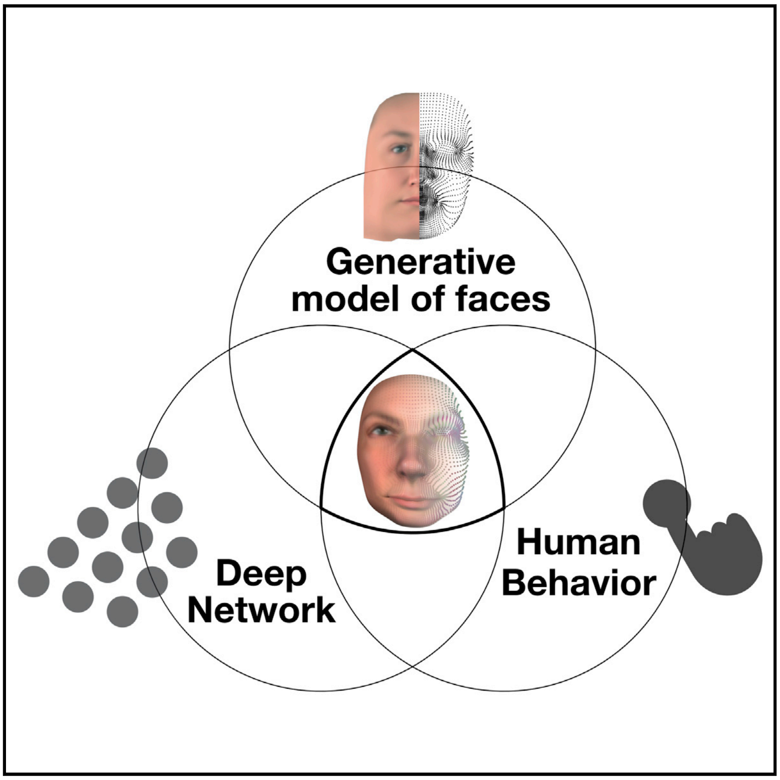

Highlights

- DNNs modeled how humans rate the similarity of familiar faces to random face stimuli
- A generative model controlled the shape and texture features of the face stimuli
- The best DNN predicted human behavior because it used similar face-shape features
- Explaining human behavior from causal features is difficult with naturalistic images

Authors

Christoph Daube, Tian Xu, Jiayu Zhan, Andrew Webb, Robin A.A. Ince, Oliver G.B. Garrod, Philippe G. Schyns

Correspondence

christoph.daube@gmail.com (C.D.), philippe.schyns@glasgow.ac.uk (P.G.S.)

In brief

With a set of five DNNs, Daube et al. model the behavior of n = 14 human participants and n = 12 human validators who identified familiar faces using a generative model of faces that controls the face features. They demonstrate that the better DNNs can predict human behavior because they use similar face shape features. They visualize the shape features that humans and DNNs jointly use and test whether these features enable generalization to new viewing angles, older age, and opposite sex conditions.

Daube et al., 2021, Patterns 2, 100348
October 8, 2021 © 2021 The Authors.
https://doi.org/10.1016/j.patter.2021.100348

CellPress

Patterns

CellPress
OPEN ACCESS

Article

Grounding deep neural network predictions of human categorization behavior in understandable functional features: The case of face identity

Christoph Daube,[1,*] Tian Xu,[2] Jiayu Zhan,[1] Andrew Webb,[1] Robin A.A. Ince,[1] Oliver G.B. Garrod,[1] and Philippe G. Schyns[1,3,*]
[1]Institute of Neuroscience and Psychology, University of Glasgow, 62 Hillhead Street, Glasgow G12 8QB, Scotland, UK
[2]Department of Computer Science and Technology, University of Cambridge, 15 JJ Thomson Avenue, Cambridge CB3 0FD, England, UK
[3]Lead contact
*Correspondence: christoph.daube@gmail.com (C.D.), philippe.schyns@glasgow.ac.uk (P.G.S.)
https://doi.org/10.1016/j.patter.2021.100348

THE BIGGER PICTURE Deep neural networks (DNNs) are often presented as "the best model" of human perception, achieving or even exceeding "human-level performance." However, it remains difficult to describe what information these DNNs process from their inputs to produce their decisions. In naturalistic images, multiple cues can lead to the same decision. For example, a DNN can identify Peter's face from his darker eyebrows or high cheekbones. However, a human knowing Peter could identify his same face with similar accuracy, but using different features (e.g. his chin or hairstyle). Decision accuracy thus only tells the visible part of the story. The hidden part is the specific information processed to decide. To address this, we compared DNNs that predicted human face identity decisions to varying faces generated with a computer graphics program. With such controlled stimuli, we revealed the hidden part of the specific face information that caused the same behavioral decisions in humans and DNNs.

1 2 3 4 5 **Proof-of-Concept:** Data science output has been formulated, implemented, and tested for one domain/problem

SUMMARY

Deep neural networks (DNNs) can resolve real-world categorization tasks with apparent human-level performance. However, true equivalence of behavioral performance between humans and their DNN models requires that their internal mechanisms process equivalent features of the stimulus. To develop such feature equivalence, our methodology leveraged an interpretable and experimentally controlled generative model of the stimuli (realistic three-dimensional textured faces). Humans rated the similarity of randomly generated faces to four familiar identities. We predicted these similarity ratings from the activations of five DNNs trained with different optimization objectives. Using information theoretic redundancy, reverse correlation, and the testing of generalization gradients, we show that DNN predictions of human behavior improve because their shape and texture features overlap with those that subsume human behavior. Thus, we must equate the functional features that subsume the behavioral performances of the brain and its models before comparing where, when, and how these features are processed.

INTRODUCTION

Visual categorization is the pervasive process that transforms retinal input into a representation that is used for higher-level cognition, such as for memory, language, reasoning, and decision. For example, to guide adaptive behaviors we routinely categorize faces as being relatively happy, aged, or familiar, using different visual features. A long-standing challenge in the field of cognitive science is therefore to understand the categorization function, which selectively uses stimulus features to enable flexible behavior.[1–3]

From a computational standpoint, this challenge is often framed as understanding the encoding function[4] that maps high-dimensional, highly variable input images to the

This is an open access article under the CC BY license (http://creativecommons.org/licenses/by/4.0/).

lower-dimensional representational space of features that serve behavior. Deep neural networks (DNNs) have recently become the model of choice to implement this encoding function. Two key properties justify popularity of DNNs: first, they can solve complex, end-to-end (e.g., image-to-behavior) tasks by gradually compressing real-world images over their hierarchical layers into highly informative lower-dimensional representations. Second, evidence suggests that the activations of DNN models share certain similarities with the sensory hierarchies in the brain, strengthening their plausibility.[5–10] Such findings underlie the surge of renewed research at the intersection between computational models, neuroscience, and cognitive science.[11]

However, there is ample and mounting evidence that DNNs do not yet categorize like humans. Arguably, the most striking evidence comes from adversarial examples, whereby a change in the stimulus imperceptible to humans can counter-intuitively change its categorization in a DNN[12] and vice versa.[13] Even deceptively simple visual discrimination tasks reveal clear inconsistencies in the comparison between humans and state-of-the-art models.[14] Furthermore, when tested with photos of everyday objects taken from unusual perspectives, DNNs trained on common databases of naturalistic images decrease in test-set performance in ways humans do not.[15] In sum, although DNNs can achieve human-like performance on some defined tasks, they often do so via different mechanisms that process stimulus features different from those of humans.[16,17]

These results suggest that successful predictions of human behavioral (or neural) responses with DNN models are not sufficient to fully evaluate their similarity, a classic argument on the shortcomings of similarity in cognitive science.[18,19] In fact, we already know that similar behaviors in a task can originate from two human participants processing different features.[20] Generalizing to the comparison of a human and their DNN model, consider the example whereby both categorize a given picture as a horse. Should we conclude that they processed the same features? Not if the DNN learned to use the incidental horse-specific watermarks from the image database.[21] This simple example illustrates both the general importance of attributing behavior to the processing of specific features, and the long-standing challenge of doing so, especially given the dense and unknown correlative structure of real-world stimuli.[22] From an information-processing standpoint, we should know what stimulus information (i.e., features) the brain and its DNN models process, before comparing where, when, and how they do so.[23,24] Otherwise, we risk studying the processing of different features without being aware of the problem (cf. watermark example above). Thus, to realize the potential of DNNs as information-processing models of human cognition,[25] we need to first take a step back and demonstrate that similar behavior in a task is grounded in the same stimulus features—i.e., more specifically, in similar functional features: those stimulus features that influence the behavioral output of the considered system.[1] When such functional feature equivalence is established, we can meaningfully compare where, when, and how the processing of these same functional features is reduced with equivalent (or different) algorithmic-implementation-level mechanisms in humans and their models.

To develop such equivalence of functional features, we explicitly modeled stimulus information with an interpretable generative model of faces (GMF).[26] The GMF allows parametric experimental control over complex realistic face stimuli in terms of their three-dimensional (3D) shape and two-dimensional (2D) RGB texture. As illustrated in Figure 1, a candidate DNN model is typically evaluated on how it predicts human responses, by computing the bivariate relationship between human responses and DNN predictions. Here, we further constrained this evaluation by relating human behavioral responses and their DNN predictions to the same set of experimentally controlled GMF features. Conceptually, this is represented as the triple intersection in Figure 1, where the pairwise intersections <GMF features; human> and <GMF features; DNN predictions> comprise the functional face features that subsume human responses and their DNN models. The triple intersection further tests whether the same responses in the two systems arise from the same face features, on the same trials. We then compared how each candidate DNN model represents these face features to predict human behavior and reconstructed the internal face representations of humans and their DNN models with reverse correlation.[27] Lastly, and importantly, we used our generative model to compare the generalization gradients of humans and DNNs to typical out-of-distribution stimuli (i.e., generalizations to changes of face pose, age, and sex to create siblings with family resemblance). With this approach, we ranked models not only according to their surface similarity of predicted human behavior but also according to the deeper similarity of the underlying functional features that subsume behavioral performance.

RESULTS

We used a generative model that parameterizes faces in terms of their 3D shape and 2D RGB texture (GMF; see "generative model of 3D faces" in experimental procedures) to control the synthesis of ~3 million 2D face images that varied in identity, sex, age, ethnicity, emotion, lighting, and viewing angles (see Figure S1 for a demonstration; see "networks, training set" in experimental procedures). We used these images to train five DNNs that shared a common ResNet[31] encoder architecture but differed in their optimization objectives. The five DNNs were as follows (see Figure 2 for their schematic architectures and performances): (1) a triplet loss network[32] that learned to place images of the same (versus different) identity at short (versus long) Euclidean distances on its final layer; (2) a classification network[33] that learned to classify 2,004 identities (2,000 random faces, plus four faces familiar to our participants as work colleagues, "ClassID"); (3) another classification network that learned to classify 2,004 identities plus six other factors of variation of the generative model ("ClassMulti"); (4) an autoencoder (AE)[34] that learned to reconstruct all input images; and (5) a view-invariant autoencoder (viAE)[35] that learned to reconstruct the frontal face image of each identity irrespective of the pose of the input.

We used these five DNNs to model the behavior of each of n = 14 individual human participants who resolved a face familiarity experiment (see "participants" in experimental procedures and Zhan et al.[26]) In this experiment, participants were asked to rate, from memory, the similarity of random face stimuli generated by the GMF (Figure 2A) to four familiar identities (see "experiments" in experimental procedures and Zhan et al.[26]) On

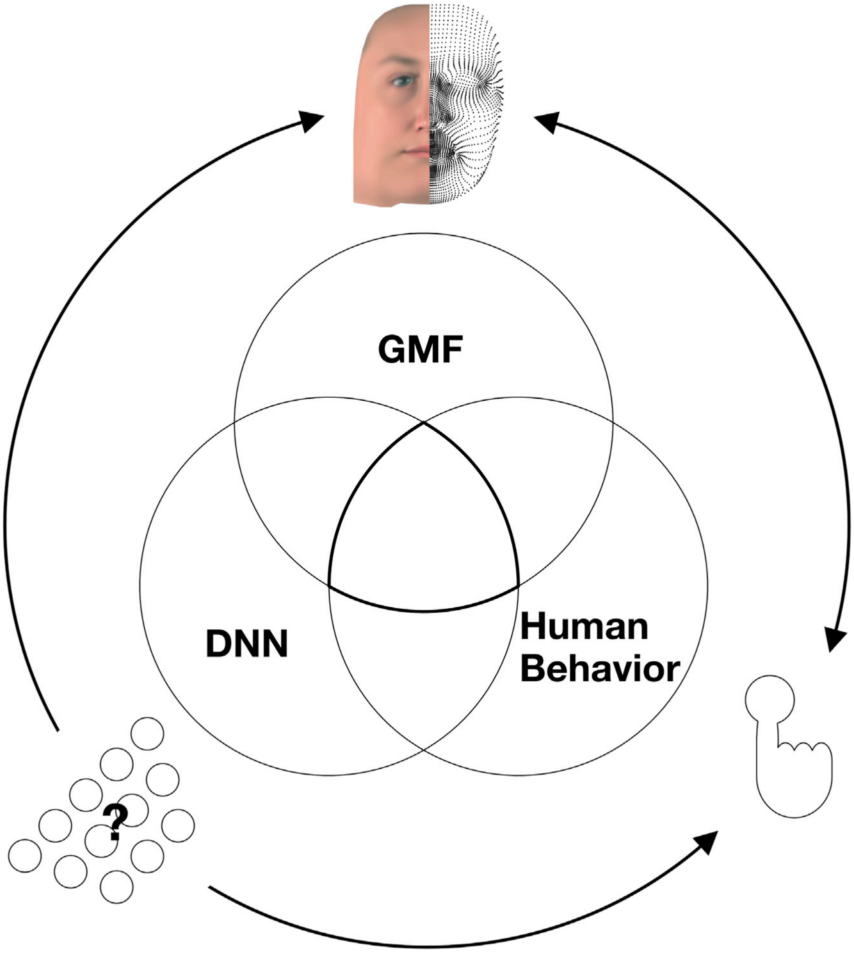

Figure 1. Trivariate relationship to understand the functional features of DNN models that predict human behavior

In general, complex visual inputs are processed in an unknown way in the brain and its DNN models to produce behavior. DNNs (schematized as layers of neurons) can predict human behavior and can in principle be used to facilitate our understanding of the inaccessible information-processing mechanisms of the brain. However, nonlinear transformations of information in DNNs complicate our understanding, in turn limiting our understanding of the mechanistic causes of DNN predictions (and human behavior). To address this issue of interpretability, we used a generative model of realistic faces (GMF) to control the high-level stimulus information (3D shape and RGB texture). The Venn diagram illustrates the logic of our approach. Human behavior and its DNN model predictions are both referred to in the same stimulus model: (1) the GMF features that underlie human behavior; (2) the GMF features that underlie DNN predictions of human behavior. The question then becomes: are these GMF features equivalent? That is, do the two intersections intersect?[28] We quantify GMF feature overlap with information theoretic redundancy[29,30]— i.e., as the information that GMF features and the activations of the embedded layers of DNN models provide about human behavior. In doing so, we assess the functional feature equivalence of individual human participants and their DNN models in relation to a specific model of the stimulus and behavioral task. See Figure 2 for a detailed overview of the analysis pipeline. Our results develop why such feature equivalence enhances our understanding of the information-processing mechanisms underlying behavior in the human brain and its DNN models.

each of 1,800 trials, each participant was presented six random faces. They were asked to first choose the face most similar to a target identity and then rate this similarity on a 6-point scale. Importantly for our modeling, we propagated these 2D images through the five DNNs and then used the activations of their respective layer of maximum compression (i.e., the "embedding layer") for the subsequent analyses detailed below.

To assess functional feature equivalence between human participants and the DNN models, we proceeded in four stages (see Figure 2 for an overview of our pipeline). First, we used the representations of the experimental stimuli on the DNNs' embedding layers to predict the corresponding behavior of humans in the experiment (Figures 2C and 2D). We did so using linear models to restrict the assessment to explicit representations.[4] We call this first stage of seeking to equate human and DNN behavior "forward modeling." In a second stage, we analyzed the face features represented on the DNN embedding layers that predict human behavior. In a third stage (Figures 2E and 2F), we used reverse correlation to reconstruct and compare these categorization features between humans and their DNN models. Lastly, in a fourth stage (Figure 2G), we compared the generalization performances of humans and DNNs under new testing conditions of face viewing angles, sex, or age that did not appear in the data used to fit the forward models.

On previewing the results of the DNN models tested, the viAE afforded the best predictions of human behavior. These could be attributed to the shape features of the GMF, which also subsumed human behavior. That is, the surface similarity of behavioral performance was grounded in a deeper similarity of functional face features. Of the DNN models tested, the viAE model was therefore the most functionally similar to humans.

Forward modeling of human behavior using DNN activations

To evaluate how accurately the compressed stimulus representations on the DNNs' embedding layers predicted the face similarity ratings (on a 6-point rating scale, see Figure S2) of human participants, we activated their embedding layers with the 1,800 2D face stimuli rated in terms of similarity to each target identity in the human experiment. We then used these activations to linearly predict the corresponding human ratings in a nested cross-validation[37] (see "forward models" in experimental procedures). We compared DNN performances with three additional benchmark models that also linearly predicted human behavior. The first model used on each trial the objective 3D shape parameters of the GMF that define the identity of each face stimulus (rather than the face image); the second one used instead the GMF texture parameters (cf. Figures 1 and 2, and 3D shape and 2D

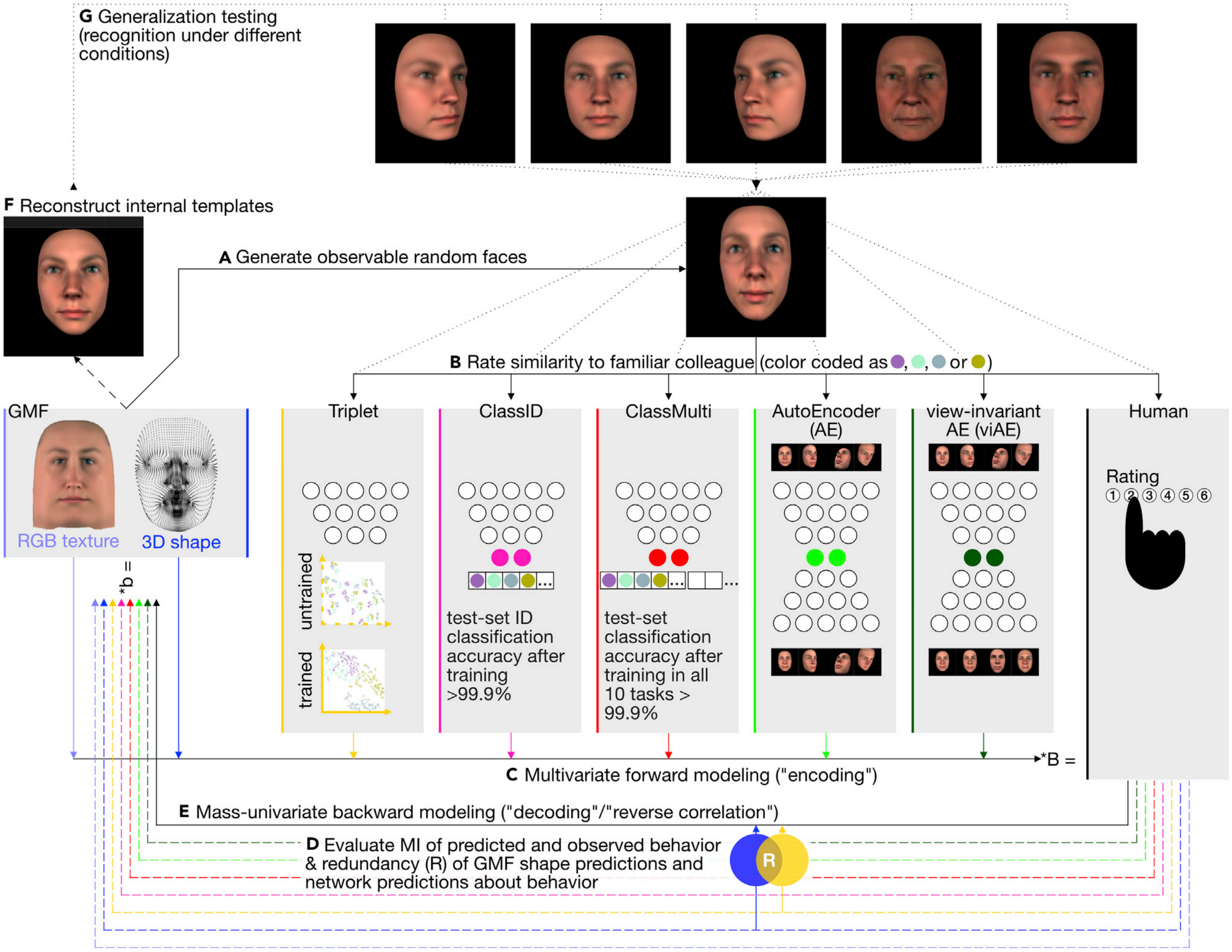

Figure 2. Study overview

We seek to establish the GMF feature equivalence between humans and their DNN models.

(A) We used the GMF to synthesize random faces (3D shape and RGB texture).

(B) We asked humans to rate the similarity of these synthesized faces to the faces of four familiar colleagues (symbolized by purple, light-blue, gray, and olive dots).

(C) Linear multivariate forward models predicted human responses (denoted by the multiplication with linear weights *B*) from GMF shape and texture features and DNN activations (DNN architectures are schematized with white circles symbolizing neurons, embedding layers are colored; scatterplots for Triplet network show two-dimensional t-stochastic neighborhood embeddings[36] of the embedding layer when activated with 81 different combinations of viewing and lighting angles per colleague). As a baseline model, we also included the first 512 components of a principal components analysis on the pixel images ("pixelPCA," not shown here).

(D) We then evaluated shared information between human behavior, DNN predictions from embedded activations, and GMF features using partial information decomposition.[36] Here, the Venn diagram shows the mutual information (MI) between human responses and their predictions based on the GMF shape features (blue circle) or based on the Triplet model (yellow circle). The overlapping region denotes redundancy (R).

(E–G) We performed reverse correlation (E) to reconstruct internal templates (F) of the familiar colleague faces from human and model predicted behavior. Lastly, we amplified either the task-relevant versus task-irrelevant features of the four colleagues (identified in E) and rendered these faces in five different generalization conditions (G) that humans and DNNs had to identify.

See also Figure S1.

RGB texture). Finally, the third model was a simpler architecture that linearly predicted human behavior from the first 512 components of a principal components analysis (PCA) of all stimulus images ("pixelPCA").

For each model, we evaluated predictions of human behavior with two information theoretic quantities (Figures 3A and 3B). With mutual information (MI), we quantified the strength of the relationship between the observed human and DNN predicted similarity ratings (Figures 3A and 3B, y axes). Importantly, we also used redundancy (from partial information decomposition)[29] to evaluate the triple set intersection of Figure 1, which quantifies the overlap between predictions from DNN models and predictions from GMF shape parameter models (Figure 3B, x axes). This overlap indicates the extent

CellPress
OPEN ACCESS

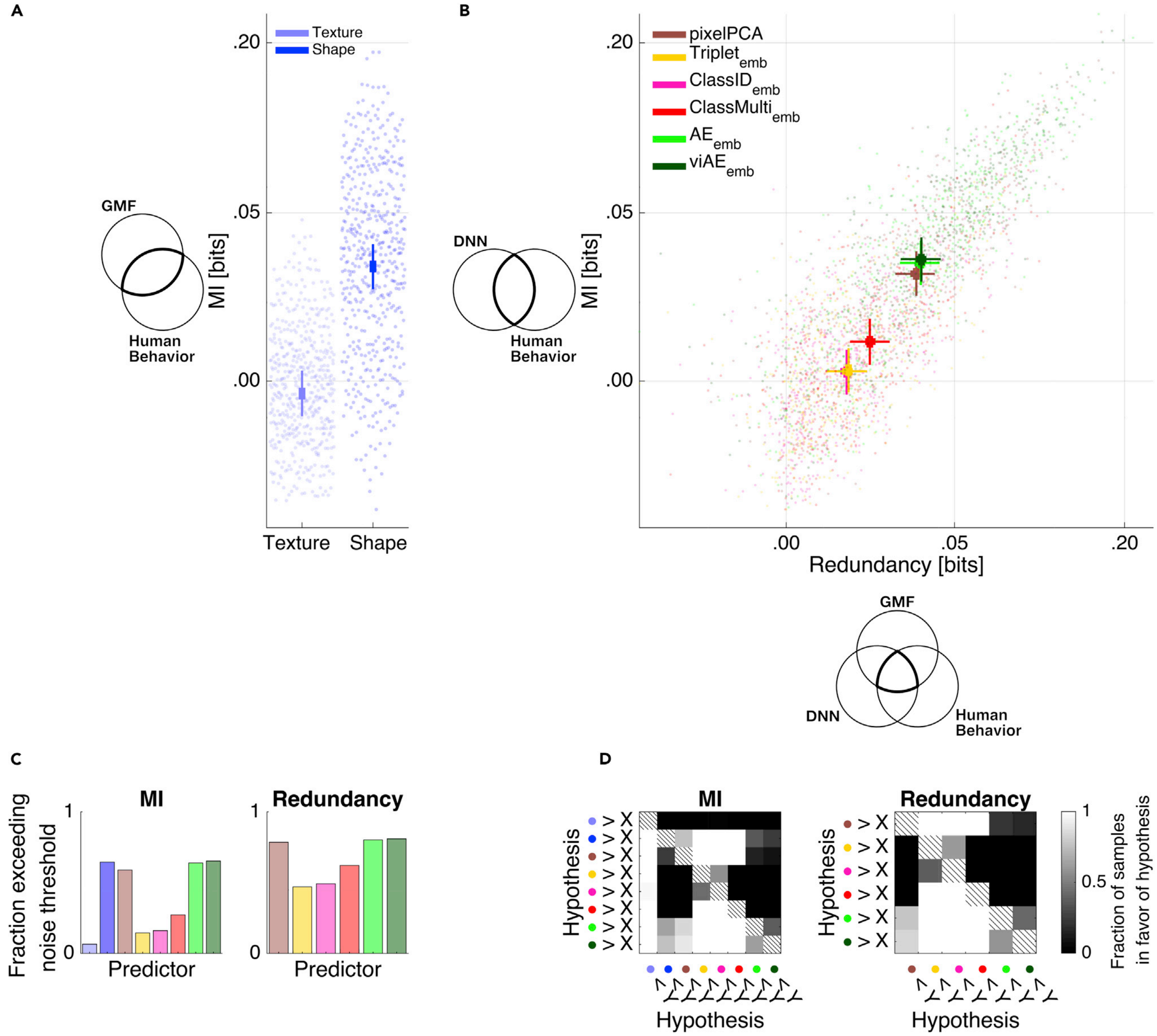

Figure 3. Relationship among GMF features, DNN activations, and observed behavior

(A) Mutual information (MI) between human behavior and test-set predictions from GMF features.

(B) y axis: MI between human behavior and test-set DNN predictions; x axis: redundant information about human behavior that is shared between DNN predictions and GMF shape feature predictions. These plots show that DNN prediction performance of human behavior increases on the y axis when the DNN embedding layers represent the same shape features as humans. Each data point in (A) and (B) represents the combination of one test set, one participant, and one familiar identity. Overlaid lines reflect the 95% (bold) and 50% (light) highest posterior density intervals (HPDIs) of the corresponding main effects of predictor spaces from Bayesian linear models fitted to the MI and redundancy values.

(C) Fractions of MI and redundancy data points exceeding noise threshold (95th percentile of MI and redundancy distributions obtained from trial-shuffled data).

(D) Comparisons of the posterior distributions of the main effects for all predictor spaces from Bayesian linear modeling of the raw performances. For each pair in the matrices, the grayscale color map shows the fraction of samples of the predictor space color coded on the y axis that is larger than the predictor space color coded on the x axis (testing a hypothesis).

Colors in (C) and (D) correspond to those in (A) and (B). See also Figures S2–S8 and S21.

to which the DNN embedding layers and the GMF shape parameters both predict the same human behaviors on the same trials. With Bayesian linear models,[38] we then statistically compared the bivariate relationships (i.e., MI) and overlaps (i.e., redundancy) of different GMF parameters and DNN embedding layers with each other.

Of all models, the viAE best predicted human behavior (see Figure 3B), closely followed by the AE, with a performance level similar to that of the GMF shape parameters (fraction of samples of posterior in favor of viAE over shape: $f_{h_1} = 0.7536$; AE > shape: $f_{h_1} = 0.6457$; $f_{h_1} = 0$ for all other networks versus shape). Surprisingly, the simple pixelPCA came close to the complex AEs (with

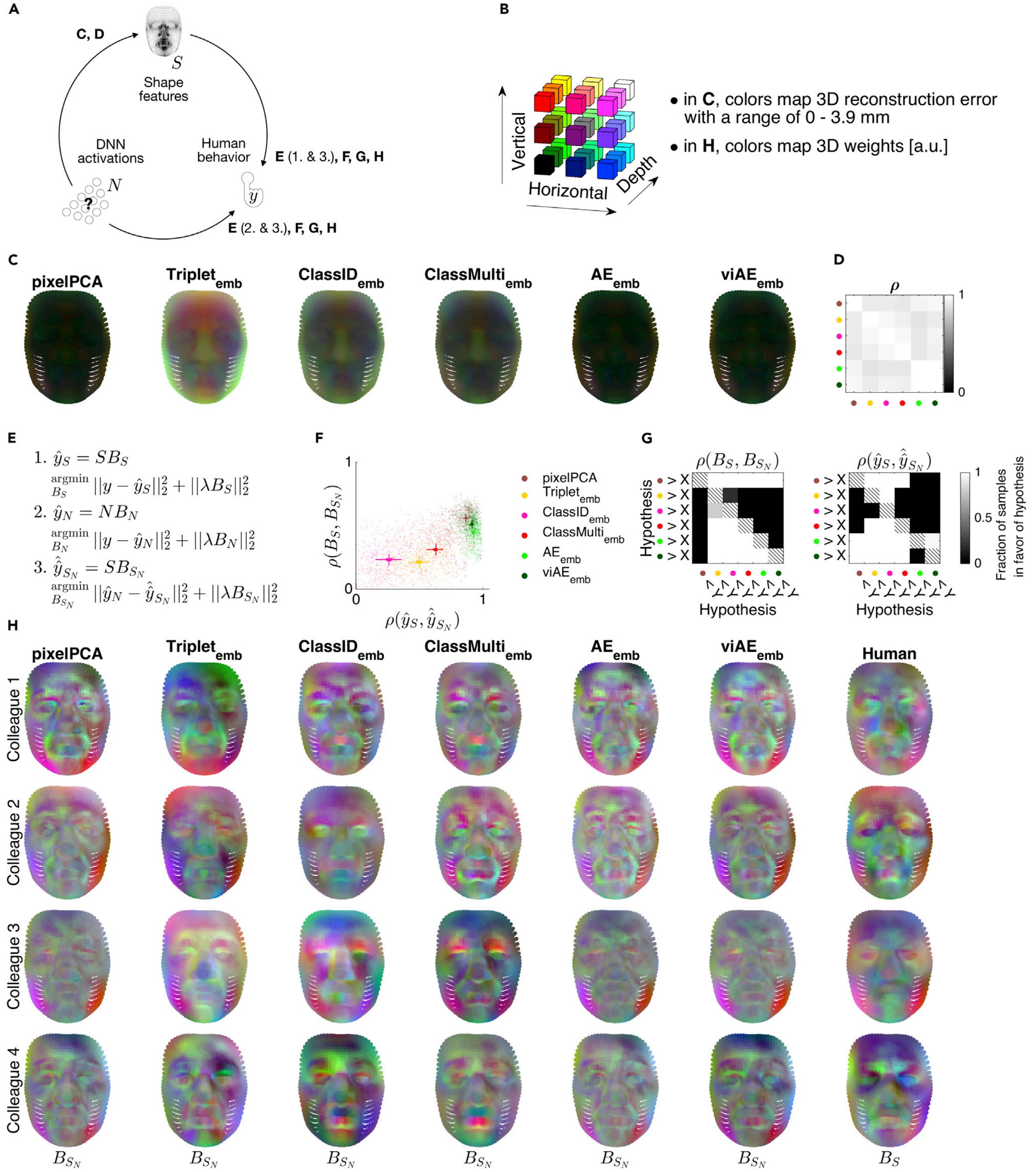

Figure 4. DNN representations of face-shape features for the forward linear models of human behavior

(A) Schema of the analyses.

(B) Legend for 3D color codes in (C) and (H).

(C) Linear readout of face-shape features from the embedding layers of the five DNNs, where readout fidelity of GMF parameters is plotted per face vertex as the mean absolute error (MAE, averaged across a large set of test faces). Higher fidelity (lower MAE) of (vi)AE activations (compared with other DNNs) shows they better represent GMF shape features.

(legend continued on next page)

the AE only narrowly beating pixelPCA, f_{h_1} = 0.8582, Figure 3B). Critically, as model predictions increased in accuracy, they also increased in overlap (i.e., redundancy) with the GMF shape parameters (Figure 3B), implying that single-trial behavior across systems (i.e., humans, viAE, and pixelPCA) could be attributed to these same specific parameters of 3D face shape—i.e., under these conditions they used the same functional face features to achieve the same behaviors.

Furthermore, we validated this overlap in shape parameters by showing that a model using jointly (vi)AE activations and GMF shape parameters (versus (vi)AE activations on their own) did not improve prediction of human behavior (see Figures S4 and S8 for additional candidate models, including combinations of the predictor spaces reported here, weighted and unweighted Euclidean distances, variational AEs, and decision neuron activities; see Figure S5 for the same comparison using Kendall's tau as an evaluation metric; see Figures S6 and S7 for a model comparison on the across-participant average). Note that the performances of these models could not be reached when predicting the behavior of participants with the behavior of other participants (see Figures S3–S5). This means that participants behaved in systematically idiosyncratic ways.

In sum, in our first stage to assess functional equivalence between humans and their DNN models, we built forward models that predicted human behavior from the DNNs' embedding layers. The embedding layer of the (vi)AE won. We further showed that better predictions of human behavior from the embedding layers of DNNs were caused by their increased representation of the 3D face features that predict human behavior. However, a simple PCA of the pixel images performed competitively. At this stage, we know that better predictions of human behavior are caused by better representations of the 3D shape features that humans use for behavior. Next, we characterized what these 3D features are.

Embedded face-shape features that predict human behavior

The viAE learned to represent on its embedding layer, from 2D images, the face-shape features that provide the best per-trial prediction of human behavior. Here, we establish: (1) how the DNNs represent these face-shape features on their embedding layers; and (2) how each feature impacts behavioral prediction in the forward models discussed in stage 1 above. We did not analyze the GMF texture features further because they could not predict human behavior (see Figure 3).

Face-shape features represented on the embedding layers of DNNs

To reveal these face-shape features, we built linear decoding models. These used the embedding layer activations to predict the positions of individual 3D vertices (see "decoding of shape information from embedding layers" in experimental procedures). We then evaluated the fidelity of their reconstructions with the Euclidean distance between the linearly predicted and the objective 3D face vertex positions. Fidelity increased from the Triplet to the two classifier networks, to the (vi)AE (which had the lowest error, see Figure 4C). The pixelPCA achieved a similarly low error, and all models shared a common type of reconstruction errors (Figure 4D) which misrepresented the depth of the peripheral and nasal face regions.

Patterns of face-shape features that predict behavior in the DNN forward models

To better understand the shape features that the aforementioned forward models used to predict human behavior, we examined their linear weights (see "forward models" in experimental procedures). The forward GMF shape model weights directly relate a 3D shape space to human behavior. Thus, their weights form an interpretable face-space pattern that modulates behavior—i.e., a "shape receptive field" (SRF), see Figure 4H (rightmost column). In contrast, the forward models based on the DNN relate (i.e., linearly weigh) DNN activations, not GMF shape parameters, to human behavior. Thus, we used an indirect approach to interpret these weights. We built auxiliary forward models that simulated (i.e., linearly re-predicted, Figure 4E) the DNN predictions of human behavior, but this time using the GMF shape parameters instead of the embedding layers. This produced interpretable SRFs (Figure 4H) with which we could therefore understand which shape features are (or are not) represented on the DNN embedding layers to predict human behavior. Specifically, we reasoned that DNN activations and GMF features would similarly predict behavior if: (1) both shared the same SRF; and (2) predictions from DNN activations were similar to their simulations based on GMF features. Our analyses revealed that the (vi)AE best satisfied these two conditions (Figures 4F and 4G). PixelPCA features were again close to the performance of the best DNN models (Figure 4F).

In this second stage to assess functional feature equivalence, we identified, at the level of individual 3D face vertices, the shape

(D) Correlation matrix of error patterns across DNNs. Colored dots on x and y axes represent each DNN model (see F for a legend). Correlating the MAE patterns from (C) across models reveals a high similarity of errors across models: vertices that are difficult to decode from Triplet activity are also difficult to decode from viAE activity.

(E) Simulating DNN predictions of observed human behavior with GMF shape features using re-predictions. First, we estimate B_S, the shape receptive fields (SRFs) that predict human behavior from GMF shape features. Second, we estimate B_N, the weights that predict human behavior from DNN activations. Third, we estimate B_{S_N}, the SRFs that predict DNN predictions of human behavior from GMF shape features.

(F) Aggregated SRF results from all participants and target familiar colleagues. x axis: correlations between original DNN predictions of human behavior and the simulated predictions; y axis: correlations between the human SRFs with DNN SRFs. The ideal DNN model should be located in the top right corner. The (vi)AE comes closest to this location. Each dot is one test set of one participant in one target familiar colleague condition. Overlaid crosses denote 95% (bold) and 50% (light) HPDIs of main effects of feature spaces from Bayesian linear models of the raw results.

(G) Comparisons of the posterior distributions of main effects of the models from Bayesian linear modeling of the results in (F).

(H) Weight profiles of forward models (SRFs) plotted on 3D scatter of vertices. From the left, simulated shape weights of each DNN forward model (see main text, schematic in A, and equations in E for explanations) and weights of the direct GMF shape forward model of human responses. Plots show results from a typical participant with the lowest average difference from the six pooled group medians in (F).

Color coding in (D), (F), and (G) is the same.

features that DNNs represent to predict (cf. "forward modeling of human behavior using DNN activations") human behavior. Of all five DNNs, we found that the (vi)AE represents face-shape vertices most faithfully, leading to the most accurate predictions of human behavior. However, the simpler pixelPCA used apparently very similar features.

Decoding the shape features with reverse correlation

So far, we have assessed the functional equivalence between human behavior and DNN-based forward models in two stages: we have quantified to what degree the DNN model predictions of human behavior are attributable to GMF face-shape parameters (in stage 1), and we have characterized how the DNN models used specific patterns of face-shape parameters to predict behavior (in stage 2). In this third stage, we use the behavior observed in humans and predicted by DNN models to reconstruct, visualize, and compare the actual 3D shape features of the target faces represented in both humans and their DNN models.

To run the human experiments[26] with the DNN models, we proceeded in three steps (see"reverse correlation" in experimental procedures). First, we used the forward models described in stage 1 to predict human behavior in response to all face stimuli of the human experiment (6 × 1,800 = 10,800 face stimuli per familiar target face).[26] On each trial, the forward models "chose" the face stimulus with the highest predicted rating from an array of 6 (see Figure S3). This resulted in 1,800 chosen faces and their corresponding similarity rating predictions. Second, for each model and participant, we regressed (mass univariately) the GMF parameters of the chosen faces on the corresponding ratings to derive a slope and intercept per GMF shape and texture parameter. Third, we multiplied these slopes by individual "amplification values" that maximized the behavioral responses (Figure 4B). The results were faces whose functional features elicited a high similarity rating in the DNN models (Figure 4C), analogous to faces that elicited high similarity ratings in each human participant, as in the original study.[26]

We then compared the functional face features of human participants and their DNN models (Figure 5D, left). We also computed how veridical these human and DNN features were to the ground truth faces of familiar colleagues (Figure 5D, right).

How human-like are DNN features?

The viAE had the most human-like features, with the lowest mean absolute error (MAE, Figure 5D, left, y axis; comparison with second best DNN model, AE > viAE: f_{h_1} = 0.9943) and a correlation with human features similar to that of the AE (Figure 5D, left, x axis; viAE > AE: f_{h_1} = 0.8489). All DNN models had a lower MAE than the simple pixelPCA model (all DNNs < pixelPCA: f_{h_1} > 0.9492), but only the (vi)AE had a better correlation with human features (AE and viAE > pixelPCA: both f_{h_1} > 0.9729).

How veridical are DNN and human features?

viAE features were closest to the veridicality of human features to the ground truth 3D faces, with the lowest MAE (Figure 5D, right, y axis; second best DNN model AE > viAE: f_{h_1} = 0.9558; viAE > human: f_{h_1} = 0.9996) and a correlation comparable with that of the AE. All DNN models had a lower MAE than the simple pixelPCA model (all DNNs < pixelPCA: all f_{h_1} > 0.9732), but only the (vi)AE had a better correlation with the ground truth face identity features (AE and viAE > pixelPCA: both f_{h_1} > 0.8842).

In sum, this analysis compared the internal representations of the target faces in human participants and their DNN models, and all with the ground truth 3D shapes of the target identities. These comparisons, supported by intuitive visualizations, revealed that the viAE had internal feature representations that best matched the internal representations of humans.

Generalization testing

A crucial test of models of human behavior is their generalization to conditions that differ from the distribution of the training data. We performed such out-of-distribution testing in five different tasks,[26] using the GMF to change the viewing angle, the age (to 80 years), and the sex (to the opposite sex) of the target familiar face (Figure 6C). Importantly, we did so while also selectively amplifying functional face features that were expected (Figure 6A) or not expected (Figure 6B) to cause the identification of each familiar face (based on reverse correlation, see "experiments—generalization testing"in experimental procedures; Zhan et al.[26]). Using these new stimuli, we compared the generalization performance of a new group of n =12 human validators and the DNN models. On each trial, validators responded by selecting the familiar identity that was most similar to the face stimulus (or used a fifth option when the stimulus was not similar to any familiar face). For each face stimulus, we predicted the human similarity ratings using the forward models fitted to each of the 14 participants and four familiar faces as described in stage 1 above, and chose the faces that yielded the highest predicted rating. We then compared the absolute error of the model choice accuracies with the human choice accuracies.

The viAE best matched human identification performance, which both increased when the functional features were amplified in the stimulus (Figures 6D–6F). The viAE had only a slightly smaller error compared with the AE for the frontal view (viAE < AE: f_{h_1} = 0.8958), but a better view invariance with a clearly smaller error for the -30° (viAE < AE: f_{h_1} = 0.9995) and $+30^\circ$ views (viAE < AE: f_{h_1} = 0.9696). Only the GMF shape feature model came close to the (vi)AE (and was better than both AEs at -30°, both f_{h_1} = 1, and $+30^\circ$, both f_{h_1} > 0.7656). However, recall that the GMF is a non-image-computable "ground truth" 3D model whose input is not affected by 2D image projection. Critically, the simple pixelPCA model did not generalize well to viewpoint changes (viAE and AE < pixelPCA: f_{h_1} = 1) except in the age generalization task, where it had a slightly lower error than the second best viAE (pixelPCA < viAE: f_{h_1} = 0.9940). In the opposite sex task, the viAE again had the lowest error (viAE < second best AE: f_{h_1} = 1).

Whereas previous analyses suggested that a model as simple as the pixelPCA could explain human responses, more comprehensive tests of the typical generalization gradients of face identity demonstrated that such a conclusion was unwarranted. Thus, rigorous comparative tests of typical generalization gradients are required to properly assess human visual categorization in relation to their DNN models.

DISCUSSION

In this study, we sought to address the long-standing problem of interpreting the information processing performed by DNN models so as to ground their predictions of human behavior in

CellPress
OPEN ACCESS

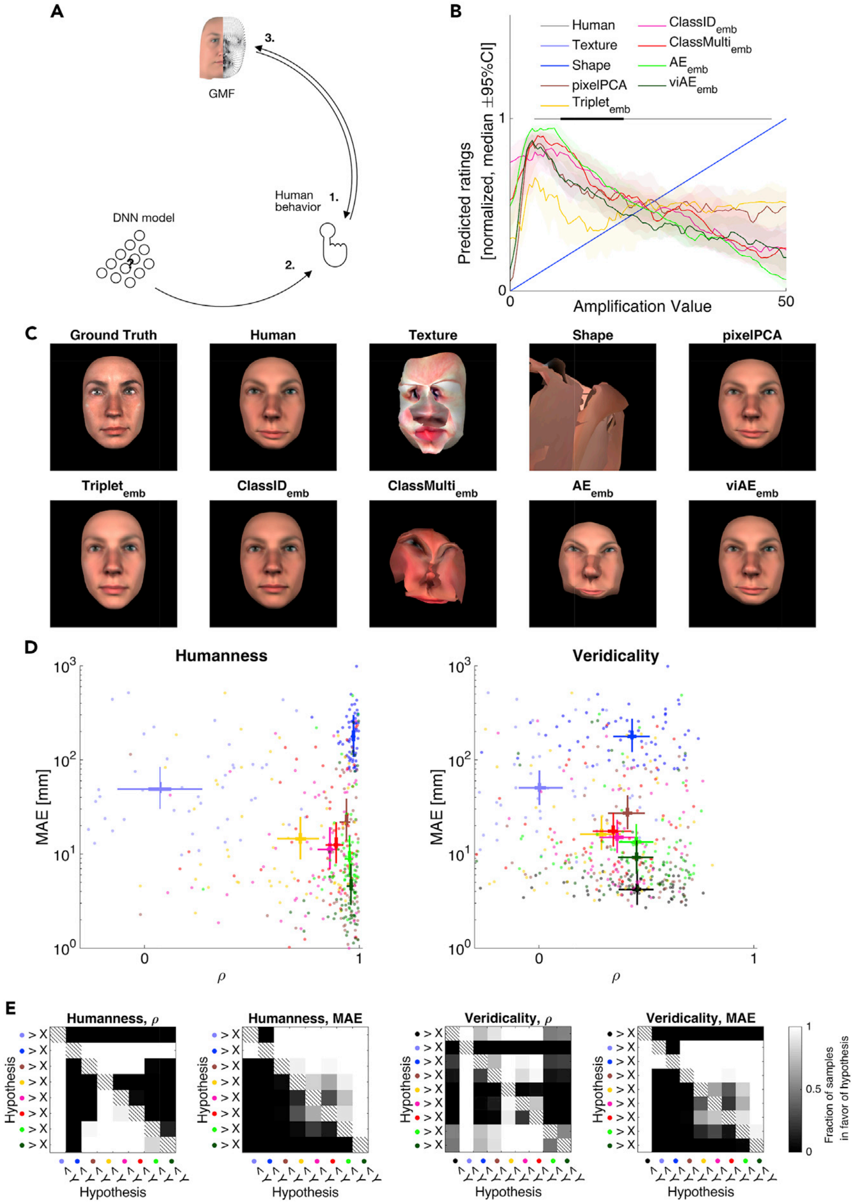

Figure 5. Internal templates reconstructed from human behavior and its model predictions

(A) Schema of analysis. We predicted human behavior from GMF features (1.) and DNN activations (2.). With mass-univariate regression, we predicted each individual GMF feature from human behavior and its DNN predictions (3.).

(B) Amplification tuning curves. We presented the reverse correlated templates amplified at different levels to each model. Solid lines denote pooled median across participants and colleagues, shaded regions denote 95% (frequentist) confidence intervals. Black lines at the top denote 95% (bold) and 50% (light) highest density estimates of human amplification values. The linear GMF shape and texture forward models predicted monotonically increasing responses for higher amplification levels. Other models peaked at a given amplification level. See Figure S9 for amplification tuning responses of a broader range of models.

(C) Comparison of rendered faces. Panels show ground truth face of one exemplary target familiar colleague captured with a face scanner (top left) and reconstructions of the face features from human behavior and its DNN predictions for one typical participant (i.e., closest to the pooled group medians shown in D). Figure S14 presents the three other familiar colleagues.

(D) Evaluation of correspondence of humans and model templates ("humanness," left) and the relation of templates to ground truth faces ("veridicality," right). The x axis shows Pearson correlation of the 3D features projected onto a single inward-outward direction; the y axis shows the mean absolute error (MAE) of the 3D features. Each dot corresponds to a single participant in a specific target familiar colleague condition. Crosses denote 95% (bold) and 50% (light) HPDIs for each system from Bayesian linear modeling of the results.

(E) Comparison of main effects of systems in Bayesian linear models of the results in (D).

See also Figures S9–S14 and S21.

interpretable functional stimulus features. Key to achieving this was our use of a generative model to control stimulus information (3D face shape and RGB texture). We trained five DNN models with different objectives, following which we activated the DNNs' embedding layers with the face stimuli of a human experiment (in which participants were asked, based on their memory, to assess the similarity of random faces to the faces of four familiar colleagues). We then used these activations to fit forward models that predicted human behavior. Of the tested models, (vi) AE embeddings best predicted human behavior, because these embeddings represented the human-relevant 3D shape of familiar faces with the highest fidelity. Next, we reconstructed the face features represented in the embeddings that impact the behavioral predictions. The 3D reconstructions demonstrated that the viAE models and humans used the most similar functional features for behavior. Lastly, we found that the viAE best matched human generalization performance in a range of five different out-of-distribution changes of the stimuli (testing several viewing angles, older age, and opposite sex versions of the four colleagues).

Together, our approach (cf. Figure 1) and analyses suggests a more stringent test of functional feature equivalence between human responses and their DNN models beyond the simple equivalence of responses to uncontrolled naturalistic stimuli. Such deeper functional features equivalence enables the mechanistic interpretations of the processing of these same features across the layers of the human brain and its DNN models. However, as shown in psychophysics, exhaustively testing the generalization gradients of human visual categorization is difficult because it requires not only modeling behavioral (or neuronal) responses but also the real-world (and artificial) dimensions of variations of the stimulus categories under consideration.

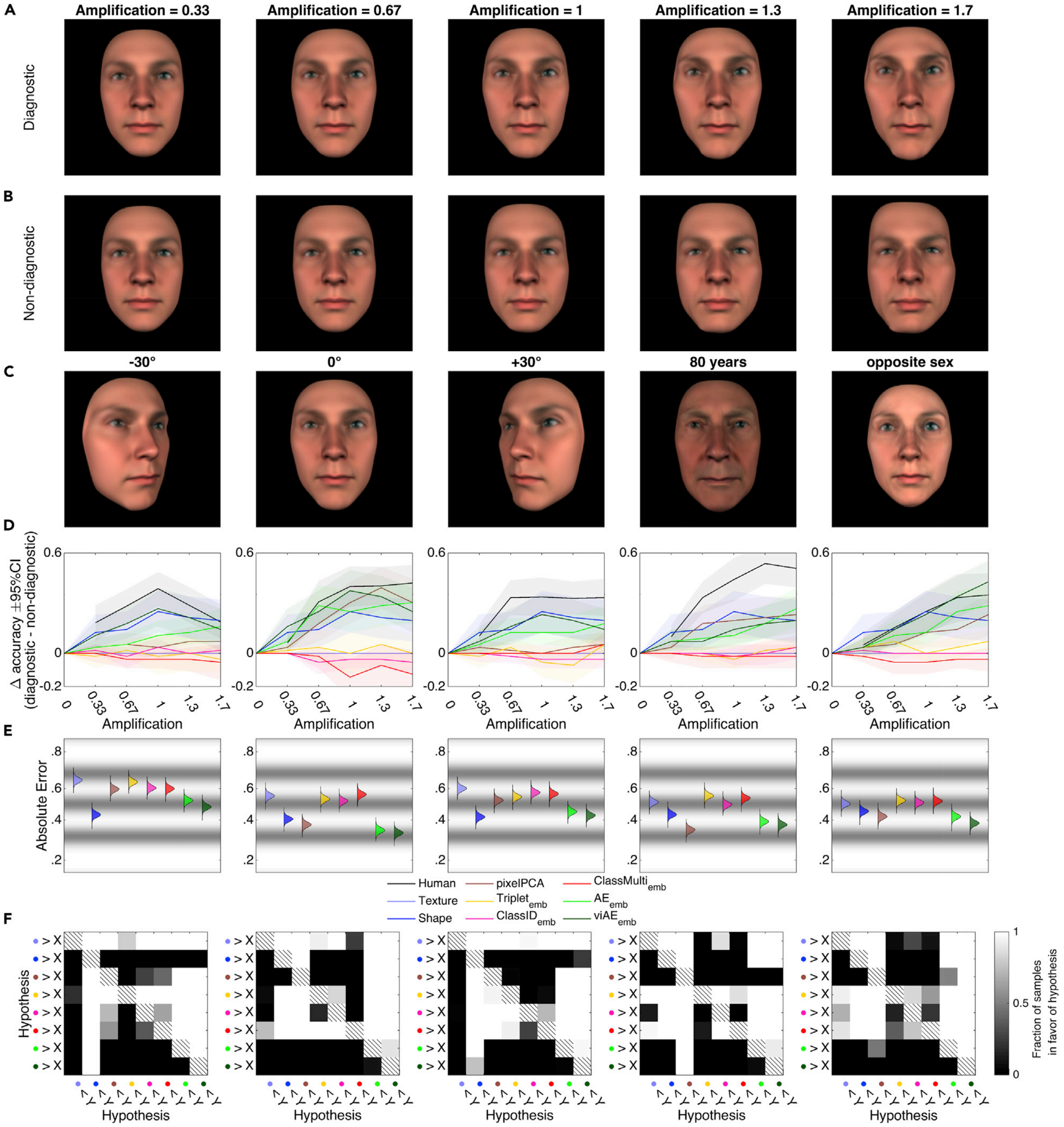

Figure 6. Generalization testing

(A) Example stimuli for the task-relevant condition in the 0° viewing angle condition of one familiar colleague. Using a group model, each face feature of each familiar identity was classified as being either task relevant or task irrelevant for human identification. Versions of each colleague were then created whereby the task-relevant (versus -irrelevant) features were amplified at different levels, while the remaining features were defined as those of the average face.

(B) Example stimuli for the task-irrelevant condition in the 0° viewing angle condition of the same target familiar identity as in (A).

(C) Renderings of the task-relevant face amplified at a level of 1.3 for five different generalization conditions.

(D) Difference of choice accuracy between the task-relevant and -irrelevant conditions. Positive values denote a higher accuracy when task-relevant features were amplified.

(E) Posterior distributions of main effects of feature spaces when modeling absolute error (relative to human behavior) with Bayesian linear models. Gray bandings denote density estimates of thresholds separating the five possible different error values (human accuracies are averaged across five ratings of the same item).

(F) Comparison of the posterior distributions in (E). For each pair in the matrices, the color gradient reflects the fraction of samples of the feature space color coded on the y axis > the predictor space color coded on the x axis.

See also Figures S15–S21.

Why focus on functional equivalence?

A key finding that motivates usage of DNNs as models of the human brain is that their activations predict behavioral and neural responses to novel real-world stimuli better than any other model. However, it remains unclear whether these surface similarities between humans and DNNs imply a deeper similarity of the underlying information-processing mechanisms.[39] Real-world stimuli comprise multiple unknown correlations of undefined features. It is generally unknown which of these features DNNs use, leading to unpredictable out-of-distribution generalizations. Consequently, it is difficult to assess the featural competence of the model that predicts the behavioral or neural responses. Surprisingly simple feature alternatives ("feature fallacy")[40,41] could explain such surface similarities.[21] Relatedly, extensive testing of the generalization gradients of humans and DNNs is required to reveal algorithmic intricacies that would otherwise remain hidden, leading to failure with out-of-distribution exemplars.

Marr's framework offers a solution to these problems:[23] we should constrain the similarity of complex information-processing mechanisms at the abstract computational level of their functional goals of seeking specific information to resolve a task. Our methodology sought to assess whether the human participants and their DNN models processed similar functional face features in a face identity task where features are defined within a generative model of the stimulus. Once functional equivalence is established, we can turn to the algorithmic-implementation levels of Marr's analysis. That is, we can seek to understand where, when, and how detailed mechanisms of the occipitoventral hierarchy, and suitably constrained DNN architectures (e.g., with two communicating hemispheres, properties of contralateral followed by bilateral representations, and so forth) process the same functional features of face identity, using a model of the stimulus.[42] Such algorithmic-implementation-level explorations could then consider estimates of the algorithmic complexity of the task[43] to regularize explanations of model predictions to be as simple as possible.[16,44–46] We see the deeper functional equivalence of the information processed as a necessary prerequisite to surface comparisons of network activations or behaviors in a task.

Hypothesis-driven research using generative models

The idea of using generative models in psychophysics and vision research is not new.[47–50] It arose from the recognition by synthesis framework,[51,52] itself an offspring of Chomsky's generative grammars. Explicit experimental hypotheses are directly tied to the parameterization of stimuli by generative models and vice versa. For example, we explicitly tested that a parameterization of faces in terms of their 3D shape and RGB texture could mediate human and DNN behavior in the task.[26,53] Our study thereby contributes to the debate about the degree to which convolutional DNNs can make use of shape information in images.[33,54–59] In this context, the exact structure of the information represented in the human brain remains an empirical question. The veridical representation implied by computer graphics models[53,60,61] is one hypothesis. Other specific ideas about face, object, and scene representations must and will be tested with different designs of generative models, including DNNs (e.g., VanRullen and Reddy,[62–64] Bashivan et al.,[62–64] Ponce et al.[62–64]). The ideal generative model for the encoding function of visual categorization would "simply" be the inverse of the function implemented by the biological networks of the brain. Such an inverse would provide the control to experiment with each stage of the brain's algorithm of the stimulus processing for visual categorizations. In the absence of such an ideal, we must develop alternative generative models to test alternative hypotheses of the brain's encoding function for categorization. Modern systems such as generative adversarial networks[65] and derivatives of the classical variational autoencoders (VAEs) such as vector-quantized VAEs[66,67] and nouveau VAEs,[68] which can be trained on large, naturalistic face databases, can synthesize tantalizingly realistic faces, complete with hair, opening up an interesting avenue for future research and applications.[69–73] However, understanding and disentangling their latent spaces remains challenging.[74,75]

viAE wins

Among the tested DNNs and across the multiple tests, the viAE provided the best face-shape representations to predict human behavior. With the notable exception of the generalization testing, the simple nonlinear pixelPCA model came close to this performance. This speaks to a model of human familiar face perception whereby the goal of feedforward processing is a view-invariant but holistic representation of the visual input. Interestingly, the Triplet, ClassID, and ClassMulti built up to this performance level (cf. Figures 3, 4, and 5). This suggests that the latent space learned to reconstruct an entire image of the input ((vi)AE) is approximated by the latent space learned when performing multiple stimulus categorizations (recall that ClassMulti learned all the categorical factors of the GMF), whereas simpler cost functions (Triplet and ClassID) yielded less informative latent spaces. Their discriminative goals can be solved with shortcuts[16] relying on a few isolated features, which are not sufficient to generalize as humans do.[76] This aligns with previous findings that multi-task learning[77–79] and generative models[80] enhance robustness against adversarial attacks and best predict behavior under severe testing.[17] In relation to faces as a broad category, future research could systematically study the number and combinatorics of categorizations (e.g., identity, sex, age, ethnicity, facial expressions) and rendering factors (e.g., illumination, pose, occlusions) that would be required to enhance the latent spaces to match (or surpass) the predictiveness of behavior of the latent space of the viAE, also across varying levels of familiarity.[81]

Note that our specific viAE model remained imperfect in its prediction of human behavior and functional similarity of features. Its architecture did not incorporate many well-known characteristics of the human visual hierarchy, including temporal, recurrent[9] processing (e.g., with multiple fixations[82] due to foveated and para-foveated image resolution),[83] contralateral, hemispherically split representations of the input, transfer of visual features across hemispheres,[84] and integration in the ventral pathway,[85] among others. An algorithmic-implementation-level explanation of the functional features learned by the viAE should be part of future research.

Constraints on the comparison of models with human behavior

Our modeling explicitly fitted regressions of multivariate features on unidimensional behavior.[4] Our attempts to directly (parameter-free) extract one-dimensional predictions of human

behavior from DNNs failed (Figure S4). Whereas models might exist to solve this problem more efficiently,[17,80] an obstacle remains in that the human task is subjective: we do not expect the behavior of a given participant to perfectly predict that of another (see Figures S3 and S4, although representations tend to converge across participants).[26,86] Participants can have their own internal representations of each target colleague,[1,86] which is impossible to predict without considering data from individual participants. From that perspective, learning an abstracted feature representation that still allows prediction of individual behavior is an attractive compromise. We implemented such a weighting, either directly as a linear combination of GMF features and DNN activations, or as a linear combination of feature- or activation-wise distances of stimuli to model representations of the target identities. For the image-computable models, these approaches did not lead to strong differences. Arbitrating between such computational accounts of human categorization behavior thus remains a question for future research.[87–89]

The interpretability of DNNs is now an important research topic. Sophisticated methods exist to visualize the stimulus features that cause the activation of a network node, such as deconvolution,[90] class activation maps,[91] activation maximization,[92–96] locally linear receptive fields,[97] or layer-wise relevance propagation.[21,98,99] These methods usually rely on the noise-free accessibility of the activations, which is not possible with humans, making these methods unsuitable to compare humans with their DNN models. This is a significant hindrance to developing a human-like artificial intelligence, which requires resolving the challenge of designing experiments and analyses that enable inferences about the hidden representations of both humans and models.[100,101]

Conclusion

We have developed an example of how we can extend mechanistic interpretations of DNN predictions of human responses, in which we progress beyond surface predictions to a functional equivalence of the features that affect behavior. We did so by controlling complex stimulus features via an interpretable generative model. The limits of what we can predict about human behavior may be defined by the limits of current computer vision models. However, within these limits, the proportion that we can meaningfully understand is defined by the ever increasing capacities of interpretable generative models of stimulus material.[102] Databases of natural images will only take us so far. Hence, we believe that future research attention should be distributed on the gamut between discriminative models to do the tasks, and generative models of the stimulus to understand what these models do.

EXPERIMENTAL PROCEDURES

Resource availability

Lead contact

Philippe G. Schyns, Philippe.Schyns@glasgow.ac.uk.

Materials availability

This study did not generate new unique reagents.

Data and code availability

Data are available in the following repository: https://osf.io/7yx28/

Code can be found in the following github repository: https://github.com/cdaube/sharedFunctionalFeatures.

Generative model of 3D faces

The generative model of 3D faces decomposes the shape and texture components of a database of 357 faces, captured with a 3D face-capture system,[103] to enable their controlled recombination. For this study, two variations of the database were created: one excluding the faces of two female target colleagues and another excluding the faces of two male target colleagues. Each of the two database subsets then consists of a [355 × 4,735 * 3] (N × vertices * XYZ) shape matrix S and 5 [355 × $800/2^i$ * $600/2^i$ * 3] (N × $X/2^{band}$ * $Y/2^{band}$ * RGB) texture matrices T_i for bands $i = 0, \ldots, 4$ of a Gaussian pyramid model.

For each of the two database subsets, the modeling is achieved in two steps. In the first step, two separate general linear models are used to estimate the linear parameters of a constant term as well as sex, age, ethnicity (coded using two dummy variables), and their two- and three-way interactions. This is done with a [355 × 12] design matrix X describing the predictor values, a [12 × 4,735 * 3] matrix A_S describing the shape coefficients, and [12 × $800/2^i$ * $600/2^i$ * 3] matrices A_{T_i} describing the texture coefficients:

$$S = XA_S + E_S, \quad \text{(Equation 1)}$$

$$T_i = XA_{T_i} + E_{T_i}. \quad \text{(Equation 2)}$$

Here, E_S [355 × 4,735 * 3] and E_{T_i} [355 × 800 * 600 * 3] are the model residuals for shape and texture, respectively. A_S and A_{T_i} are estimated using least-squares linear regression.

In the second step, the residual components E_S and E_{T_i} are then isolated by removing the linear effects of ethnicity, sex, and age as well as their interactions from S and T_i. Next, singular value decomposition (SVD, using MATLAB's economy-sized decomposition) is performed to orthogonally decompose the shape and texture residuals:

$$U_S S_S V_S^T = E_S, \quad \text{(Equation 3)}$$

$$U_{T_i} S_{T_i} V_{T_i}^T = E_{T_i}. \quad \text{(Equation 4)}$$

The matrices U_S [4,735 * 3 × 355] and U_{T_i} [$800/2^i$ * $600/2^i$ * 3 × 355 for each of $i = 0, \ldots, 4$ spatial frequency bands] can thus be used to project randomly sampled shape or texture identity vectors into vertex or pixel space, respectively.

Any single face can then be considered as a linear combination of two parts: a basic "prototype face" defined by its factors of sex, age, and ethnicity and a specific individual variation on that prototype defined by its unique component weights. Once we know these two parts of the individual face, e.g., by random sampling, we are free to change one or the other, producing for example the same individual at a variety of different ages. This can then be rendered to an observable image with a desired viewing and lighting angle.

Participants

Ratings of random faces

To obtain behavioral data from humans, we recruited seven male and seven female white Caucasian participants aged 25.86 ± 2.26 years (mean ± SD).

Generalization testing

For a second validation experiment, 12 separate participants (7 white Caucasian female and 1 East Asian females, 5 white Caucasian males aged 28.25 ± 4.11 years [mean ± SD]) were recruited.

In both experiments, all participants had been working at the Institute of Neuroscience and Psychology at the University of Glasgow for at least 6 months and were thus familiar with the target faces. All participants had normal or corrected-to-normal vision, without a self-reported history or symptoms of synesthesia, and/or any psychological, psychiatric, or neurological condition that affects face processing (e.g., depression, autism spectrum disorder, or prosopagnosia). They gave written informed consent and received UK£6 per hour for their participation. The University of Glasgow College of Science and Engineering Ethics Committee provided ethical approval for both experiments.

Experiments

Ratings of random faces

Four sets of 10,800 random faces were generated, one for each of the four target colleagues. Two sets of random faces were created using the GMF

that was built with the database that excluded the two female target colleagues. The other two sets of random faces were created using the GMF built with the database that excluded the two male target colleagues. The demographic variables were fixed (sex, age, and ethnicity) to those of the target colleagues. The resulting faces were rendered at frontal viewing and lighting angles. For each participant and target colleague, the generated faces were randomly gathered into 1,800 groups of 2 × 3 arrays, which were superimposed on a black background. In a given trial, these face arrays were shown on a computer screen in a dimly lit room while the participant's head was placed on a chin rest at a 76 cm viewing distance from the image, such that each face subtended an average of 9.5° × 6.4° of visual angle. Participants were instructed to choose the face of the array that most resembled that of the target colleague by pressing the corresponding button on a keyboard. The screen then changed to display the instruction to rank the chosen face with respect to its similarity to the target colleague on a 6-point rating scale, ranging from 1 ("not similar") to 6 ("highly similar").

These trials were split into four sets of 90 blocks of 20 trials each, resulting in a total of 7,200 trials that all participants completed over several days.

Generalization testing

For each target colleague, 50 new 3D face stimuli were generated. These comprised the combinations of two levels of diagnosticity at five levels of amplification, which were each rendered in five different generalization conditions. Each of these factors will be explained in the following.

In the original analysis,[26] the mass-univariate reconstructions from observed human behavior (see "reverse correlation" below) had been referenced to reconstructions from 1,000 permuted versions of the responses (using the same amplification values). For each vertex, the Euclidean distance of the chance reconstruction to the categorical average had been signed according to whether it was located inside or outside of the categorical average and averaged across permutations ("chance distance"). This was repeated using the ground truth target colleague shape ("ground truth distance") as well as the human-reconstructed shape ("human-reconstructed distance"). If the absolute difference of the chance distance and the ground truth distance was larger than the absolute difference of the human-reconstructed distance and the ground truth distance, the vertex was classified as "faithful." This had resulted in a 4,735 × 14 * 4 binary matrix which had then been decomposed into matrices W [4,735 × 8] and H [8 × 56] (each column corresponding to a combination of a participant and a target colleague) using non-negative matrix factorization. Any of the eight component columns in W had been classified as contributing to a group representation of the target colleagues if the median of the loadings H across participants surpassed a threshold value of 0.1. The "diagnostic component" C_D of each target colleague had then been defined as the maximum value on that vertex across components considered to load on the respective target colleague representation. After construction, C_D had then been normalized by its maximum value. Its "non-diagnostic" complement C_N was then defined as $C_N = 1 - C_D$. Taken together, the vectors C_D and C_N could now be interpreted as reflecting to what degree each vertex contributed to the faithful representation of each target colleague across the group of participants.

These diagnostic and non-diagnostic components could then be used to construct 3D faces containing varying levels of either diagnostic (F_D) or non-diagnostic (F_N) shape information:

$$F_D = G * C_D * \alpha + XA_S\,(1 - C_D * \alpha), \quad \text{(Equation 5)}$$

$$F_N = G * C_N * \alpha + XA_S\,(1 - C_N * \alpha). \quad \text{(Equation 6)}$$

Here, G reflects the ground truth representation of the respective colleagues recorded with the 3D camera array, α reflects an amplification value that was set to one of five levels (0.33, 0.67, 1, 1.33, 1.67), and X describes the sex, ethnicity, age, and interaction values that describe the respective colleague such that XA_S represents the categorical average (see "generative model of 3D faces").

Each of these ten faces per target colleague were rendered at the viewing angles −30°, 0°, and +30° as well as with their age factor set to 80 years and a swapped sex factor.

The 12 validation participants completed three sessions (3 viewpoints, age, and sex) in a random order, with one session per day. On a given trial, the validators saw a central fixation cross for 1 s, followed by a face stimulus on a black background for 500 ms. They were then asked to classify the seen face as showing one of the four target colleagues (or their siblings or parents in the age and sex conditions) or "other" if they could not identify the face as accurately and quickly as possible. Between each trial, a fixation cross was shown for a duration of 2 s. Each stimulus was shown five times in a randomized order. In the viewpoint session, validators completed 15 blocks of 41 trials; in the age and sex sessions, validators completed 5 blocks of 44 trials. This yielded accuracies of either 0, 0.2, 0.4, 0.6, 0.8, or 1 for each of the 10 stimuli per target colleague.

Networks

Training and testing of the networks was performed in Python 3.6.8 using keras 2.2.4[104] with a tensorflow 1.14.0 backend.[105] All networks shared the same training and testing sets and were constructed using the same encoder module. All models were trained using three data augmentation methods (random shifts in width and range by 5% as well as random zooms with a range of 10%).

Training and testing sets

The networks were trained on observable images generated by the GMF. We created 500 random identity residuals and combined them with the four combinations of two sexes (male and female) and two types of ethnicity (Western Caucasian and East Asian). To these, we added the four target colleagues, resulting in a total of 2,004 identities. We rendered these at three different ages (25, 45, and 65 years), seven different kinds of emotion (happy, surprise, fear, disgust, anger, sadness, neutral), and three different horizontal and vertical viewing and lighting angles (−30°, 0°, and 30°), resulting in 3,408,804 images at a resolution of 224 × 224 RGB pixels. The four colleagues were rendered with two versions of the GMF built on face database subsets that excluded the two target colleagues of the same sex. Fifty percent of the 2,000 random identities were rendered with one of these two GMFs. This dataset had first been generated for experiments not including the data from the human experiment. The version of the GMF that had been used to generate the stimuli for the human experiment had slight differences (rescaling of the data from the face database and different range of random coefficients). To allow for effortless generalization to the slightly different statistics of the stimuli that had been generated for the human experiment, we rendered all 3,408,804 images twice, once with each of the two versions, effectively resulting in a further data augmentation. For the purpose of training, development, and testing, the dataset of 6,817,608 images was split into a training set containing 80% of the images, and into a development and test set each containing 10% of the images.

Encoder module

We used a ResNet architecture to encode the pixel space images into a low-dimensional feature space.[31] The 224 × 224 RGB images were first padded with three rows and columns of zeros, then convolved with 64 7 × 7 filters with a stride of 2, batch normalized, subjected to a rectifying linear unit (ReLU) nonlinearity, max-pooled in groups of 3 × 3, and propagated through four blocks with skip connections, across which an increasing number of 3 × 3 filters was used (64, 128, 256, and 512), with a default stride of 1 in the first block and a stride of 2 in the remaining three blocks. In each skip block, the input was first convolved with the corresponding filters and default stride, then batch normalized and subjected to a ReLU function, then convolved with filters corresponding to the current block, however with a stride of 1, batch normalized and then added to a branch of the input that was only convolved with a 1 × 1 filter with default stride and batch normalized. The resulting activation was again subjected to an ReLU nonlinearity. After four of these blocks, an average pooling on groups of 7 × 7 was applied.

Triplet

We used SymTriplet loss,[106,107] a version of the triplet loss function ("FaceNet").[32] To do so, we connected the encoder module to a dense mapping from the encoder output to a layer of 64 neurons. We then fed triplets of images to this encoder, consisting of an "anchor," a "positive," and a "negative," where the anchor and positive were random images constrained to be of the same identity while the negative was an image constrained to be of a different identity. The loss function then relates these three images in the 64-dimensional embedding space such that large Euclidean distances between anchor and positive, and short distances between anchor and negative, are penalized, as are short distances between positive and negative images. When training the parameters of this network, this yields a function that places samples of

the same identity close to each other in the embedding space. The triplet loss network was trained with stochastic gradient descent with an initial learning rate of 10^{-3} until no more improvements were observed, and fine-tuned with a learning rate of 10^{-5} until no more improvements were observed.

ClassID

Here, we connected the encoder module to a flattening operation and performed a dense mapping to 2,004 identity classes. We performed a softmax activation and applied a cross-entropy loss to train this classifier.[33] We trained the ClassID network with a cyclical learning rate[108] that cycled between a learning rate of 10^{-6} and 0.3.

ClassMulti

This network was the same as the ClassID network; however, it classified not only the 2,004 identity classes but also all other factors of variation that were part of the generation: the 500 identity residuals, the two sexes, the two ethnicities, the three ages, and the seven emotional expressions, as well as the three vertical and horizontal viewing and lightning angles. For each of these extra classification tasks, a separate dense mapping from the shared flattened encoder output was added to the architecture.[33] We trained the ClassMulti network with a cyclical learning rate[108] that cycled between a learning rate of 10^{-6} and 0.3.

Autoencoder

For this architecture, we connected the encoder module to two branches, each consisting of a convolution with 512 1 × 1 filters and a global average pooling operation. This was then connected to a decoder module, which upsampled the 512-D vector back into the original 224 × 224 RGB image space. To do so, we used an existing decoder ("Darknet" decoder).[109] In brief, this decoder upsamples the spatial dimension gradually from a size of 1–7 and then in five steps that each double the spatial resolution to reach the resolution of the final image. Between these upsampling steps, the sample is fed through sets of blocks of convolution, batch normalization, and ReLU with the number of filters alternating between 1,024 and 512 in the first set of five blocks, between 256 and 512 in the second set of five blocks, between 256 and 128 in the third set of three blocks, between 128 and 64 in the fourth set of three blocks, staying at 64 in the fifth set of one block, and alternating between 32 and 64 in the last set of two blocks. The filter size in all of these blocks alternated between 3 × 3 and 1 × 1. Finally, the 224 × 224 × 64 tensor was convolved with three filters of size 1 × 1 and passed through a tanh nonlinearity.

The loss function used to optimize the parameters of this network is the classic reconstruction loss of an AE, operationalized as the MAE of the input image and the reconstruction in pixel space. We trained the AE using the Adam optimizer[110] with an initial learning rate of 10^{-3} until no further improvements were observed.

View-invariant autoencoder

This network shared its architecture and training regime with the AE; however, we changed the input-output pairing during training. Instead of optimizing the parameters to reconstruct the unchanged input, the goal of the viAE was to reconstruct a frontalized view, independent of the pose of the input, while keeping all other factors of variation constant. This resulted in a more view-invariant representation in the bottleneck layer compared with the AE.[35]

Variational autoencoder

For this architecture,[111] we connected the encoder module to two branches, each consisting of a convolution with 512 1 × 1 filters and a global average pooling operation. These were fed into a sampling layer as mean and variance inputs, transforming an input into a sample from a 512-D Gaussian with specified mean and diagonal covariance matrix.

This sample was then fed into the same decoder module as described for the AE and viAE above.

The loss function used to optimize the parameters of this network is the sum of two parts: The first is the reconstruction loss of a classic autoencoder, for which we used the MAE between the reconstruction and the original image. The second part is the Kullback-Leibler divergence measured between the multivariate normal distribution characterized by the mean and variance vectors passed into the sampling layer and the prior, a centered, uncorrelated, and isotropic multivariate normal distribution. The second part can be seen as a regularization that effectively leads to a continuous latent space. As it has been reported that weighing the second part of the loss function stronger than the first part can improve the disentanglement of the resulting latent space ("beta-VAE"),[112] we also repeated the training with several values of the regularization parameter beta. However, this did not substantially change the latent space that we obtained.

We also trained two additional identity classifiers that used the frozen weights of the (beta = 1)-VAE. The first directly connected the VAE encoder to a dense linear mapping to 2,004 identity classes. The second first passed through two blocks of fully connected layers of 512 neurons that were batch normalized and passed through an ReLU nonlinearity before the dense linear mapping to identity. In both cases, a softmax activation function was applied and the resulting networks were trained with a cross-entropy loss function. All models shared the training regime of the AE and viAE models as described above.

Forward models

We were interested in comparing the degree to which various sets or "spaces" of predictors describing the rated stimuli were linearly relatable to the human behavioral responses. To do so in a way that minimizes the quantification of just overfitting, we linearly regressed the ratings on a range of different descriptors extracted from the random faces presented on each trial in a cross-validation framework.

The predictor spaces we used for this (each consisting of multiple predictor channels) were the texture and shape components of the single trials, as provided by the GMF, as well as the activations of the networks on their "embedding layers," as obtained from forward passes of the stimuli through the networks. Specifically, we used the 512-dimensional, pre-decision layers of the classifiers (ClassID and ClassMulti), the 64-dimensional final layer of the triplet loss network, and the 512-dimensional bottleneck layer of the AE, viAE, and VAE. We then also propagated images of the four target colleagues as recorded with the 3D capture system, fit by the GMF, and rendered with frontal viewing and lighting angles through the four networks, and computed the Euclidean distances on the embedding layers between the random faces of each trial and these ground truth images. We extended this by computing the channel-wise distances of each feature space and using them as an input to the regression described below to obtained weighted Euclidean distances. Additionally, we extracted the pre-softmax activity ("logits") of the decision neurons trained to provide the logits for the four target colleagues in the final layer of the classifier networks (ClassID and ClassMulti, as well as the linear and nonlinear VAE classifiers). Since we were interested in assessing to what degree the GMF shape and texture features and various embedding layer activations provided the same or different information about the behavioral responses, we also considered models with joint predictor spaces consisting of the two subspaces of shape features and AE, viAE, or VAE activations as well as the three subspaces of shape features, texture features, and AE, viAE, or VAE activations. Lastly, to assess the extent to which a simple linear PCA could extract useful predictors from the images, we performed an SVD on the nonzero channels, a subset of the training images used for the DNNs. Performing SVD on the entire set of training images used for the DNNs would have been computationally infeasible. The subset we used consisted of 18,000 RGB images of all 2,000 identities rendered at nine different viewing angles, limiting emotion expression to the neutral condition and lighting angles to frontal angles. The first 512 dimensions could account for 99.5976% of variance in the training set. We projected the experimental stimuli onto these for further analyses.

We performed the regression separately for each participant and target colleague in a nested cross-validation procedure.[37] This allowed us to continuously tune the amount of L2 regularization necessary to account for correlated predictor channels and avoid excessive overfitting using Bayesian adaptive direct search (BADS),[113] a black-box optimization tool (see Daube et al.[41] for a comparable approach). Specifically, we divided the 1,800 trials per participant into folds of 200 consecutive trials each and, in each of nine outer folds, assigned one of the resulting blocks to the testing set and eight to the development set. Then, within each of the nine outer folds, we performed eight inner folds, where one of the eight blocks of the development set was assigned to be the validation set and seven were assigned to the training set. In each of the eight inner folds, we fitted an L2 regularized linear regression ("ridge regression") using the closed form solution

$$B = (X'X + R)^{-1} X'y, \qquad \text{(Equation 7)}$$

where B denotes the weights, y denotes the $n \times 1$ vector of corresponding human responses, R describes a regularization matrix, and X denotes the matrix of trials $n \times$ predictors M, where

$$M = \sum_{s=1}^{o} m_s, \quad \text{(Equation 8)}$$

such that o denotes the number of combined predictor subspaces and m_s describes the number of predictor channels in the sth subspace. In the cases where the features were combinations of multiple feature subspaces, i.e., where o>1, we used a dedicated amount of L2 regularization for each subspace. This avoids using a common regularization term for all subspaces, which can result in solutions that compromise the need for high and low regularization in different subspaces, which fails to optimally extract the predictive power of the joint space. The regularization matrix R can then be described as

$$R = diag(\lambda_{1_1}, ..., \lambda_{m_1}, \lambda_{1_2}, ..., \lambda_{m_2}, ..., \lambda_{1_o}, ..., \lambda_{m_o}), \quad \text{(Equation 9)}$$

where λ_{c_s} describes the amount of L2 regularization for channel c of predictor subspace s, which is constant for all c in one s. For each predictor subspace, λ_{c_s} thus was one hyperparameter that we initialized at a value of 2^{17} and optimized in BADS with a maximum of 200 iterations, where the search space was constrained within the interval [2^{-30}, 2^{30}]. The objective function that this optimization maximized was Kendall's tau, as measured between predicted and observed responses of the inner fold validation set. We used the median of the optimal λ_{c_s} across all inner folds and retrained a model on the entire development set to then evaluate it on the unseen outer fold.

This yielded sets of 200 predicted responses for each test set of the nine outer folds. We evaluated them using two information theoretic measures: MI and redundancy, both computed using binning with three equipopulated bins.[114] We computed bivariate MI with Miller-Madow bias correction between the predictions of each forward model and the observed human responses. We also computed redundancy, using a recent implementation of partial information decomposition (PID), I_{ccs}.[29] When there are two source variables and one target variable, PID aims to disentangle the amount of information the two sources share about the target (redundancy), the amount of information each source has on its own (unique information), and the amount of information that is only available when considering both sources. In our case, we were interested in quantifying how much information the predictions derived from DNN-based forward models shared with the predictions derived from GMF shape features about observed human behavior. To assess whether the amount of MI and redundancy exceeded chance level, we repeated the nested cross-validation procedure 100 times for each combination of participant and target colleague, each time shuffling the trials. From these surrogate data, we estimated null distributions of MI and redundancy and defined a noise threshold within each participant and target colleague condition as the 95th percentile of MI and redundancy measured in these surrogate data. We counted the number of test folds of all participants and colleagues that exceeded this noise threshold and report this as a fraction relative to all data points.

To then assess whether different predictor spaces gave rise to different levels of MI and redundancy in the presence of high between-subject variance, we employed Bayesian linear models as implemented in the brms package,[38] which provides a user-friendly interface for R[115] to such models using Stan.[116] Specifically, we had performances (MI and redundancy) for each of the nine outer folds b for each combination of target colleague j, participant i, and all predictor spaces f_1 to f_q. The factor of interest were the predictor spaces f. We used Hamiltonian Monte-Carlo sampling with four chains of 4,000 iterations each, 1,000 of which were used for their warm-up. The priors for standard deviation parameters were not changed from their default values, i.e., half-Student-t distributions with three degrees of freedom, while we used weakly informative normal priors with a mean of 0 and a variance of 10 for the effects of individual predictor spaces. Specifically, we modeled the log-transformed and thus roughly normal distributed MI and redundancy as performances k with the following model:

$$k_n \sim N(\mu_n, \sigma^2), \quad \text{(Equation 10)}$$

$$\sigma \sim |t(3, 0, 10)|,$$

$$\mu_n \sim \beta_{i:f[n]} + \beta_{i:b[n]} + \beta_{i:j[n]} + \beta_{f_1[n]} + ... + \beta_{f_q[n]},$$

$$(\beta_{i:f}, \beta_{i:b}, \beta_{i:j}) \sim N(0, \sigma^2_{\beta_{int}}),$$

$$\sigma^2_{\beta_{int}} \sim |t(3, 0, 10)|,$$

$$(\beta_{f_1}, ..., \beta_{f_q}) \sim N(0, 10).$$

To compare the resulting posterior distributions of the parameters of interest, we evaluated the corresponding hypotheses using the brms package—$\beta_{f_a} - \beta_{f_b}$>0 for all possible pairwise combinations of predictor spaces—and obtained the proportion of samples of the posterior distributions of differences that were in favor of the corresponding hypotheses.

As well as the predictions, the forward models also produced weights that linearly related predictors to predicted responses. We were interested in examining these weights to learn how individual shape features were used in the forward models. For the forward models, predicting responses from shape features was directly possible: the weights B_S mapped GMF shape features to responses and could thus be interpreted as the "shape receptive field." However, to be able to compare these weights on the vertex level, we used a differently scaled version of the shape features. This was obtained by multiplying the 4,735 * 3D Z-scored 3D vertex level shape features with the pseudoinverse of the matrix of left-singular vectors U_S from the SVD performed on the identity residuals of the 3D vertex features of the face database (see "generative model of 3D faces"). This 355-dimensional representation of the shape features performed virtually identically to the unscaled version in the forward modeling. For visualization, we could then project the weights B_S from the 355D PCA component space into the 4,735 * 3D vertex space, where the absolute values could be coded in RGB space. This resulted in a map that indicated how the random faces at each vertex affected the response predictions in the three spatial dimensions.

The weight maps B_N that form the forward models that relate DNN activations to responses were less simple to study in this shape space, since they mapped the less interpretable network activations, not GMF shape features, to behavioral responses. To interpret these models in vertex space, we re-predicted ("simulated") the response predictions $\hat{y}$ derived from DNN features with the GMF shape features to obtain re-predictions $\hat{\hat{y}}$ as well as weights B_{S_N}. We reasoned that response predictions of the ideal DNN model should be perfectly predictable by the shape features and that the corresponding simulated shape weights B_{S_N} should be identical to the original shape weights B_S in this case. We thus correlated the simulated response predictions with the DNN response predictions, as well as the simulated shape weights with the original shape weights for each test fold in each participant for each target colleague condition.

Decoding shape information from embedding layers

To understand what shape information is available on the embedding layers of the networks, independently of human behavior, we trained linear models that decoded GMF shape PCA components from embedding layer activations in response to images of faces. We used a cross-validation framework on the full set of stimuli, consisting of 43,200 RGB images and their corresponding GMF shape PCA components, using a random set of 80% of the images for training, a further 10% for tuning, and the remaining 10% for testing. Specifically, we trained mass-multivariate L2 regularized regressions, separately predicting each GMF shape component from all neurons of the DNN embedding layers. Similar to the approach taken for the forward models, we tuned the L2 regularization using BADS to maximize the prediction performance on the tuning set. We then projected all predicted GMF shape PCA components into vertex space and, at each vertex, assessed the Euclidean distance between the original GMF shape model and the predictions from the DNN embedding layers.

Reverse correlation

To reconstruct internal templates of the target colleagues' faces under the GMF, we performed a mass-univariate linear mapping from the observed behavior of the human participants to each GMF shape and texture feature.

We repeated this with the choice behavior and rating behavior predicted by the forward models to compare these forward models, human observed behavior, and the ground truth shape information of the target colleagues as captured by our 3D camera array.

We performed the linear regressions of variation in the shape vertices and texture pixels of the random stimuli on the ratings of the images chosen by the human participants and their forward models based on GMF features, as well as DNN and PCA activations. This was done separately for each vertex and spatial dimension, as well as for each pixel and RGB dimension. In principle, this is equivalent to inverting the weights of the forward model.[117,118] However, to match the procedure in Zhan et al.,[26] we re-estimated these parameters per vertex and pixel using the MATLAB function "robustfit."

Each of the $v = 1, \ ..., \ 4735 * 3$ shape vertex positions s was thus modeled as

$$s_v = b_{0_v} + b_{1_v} * r, \qquad \text{(Equation 11)}$$

and each of the $p = 1, \ ..., \ 800 * 600 * 3$ texture pixel RGB values t was modeled as

$$t_p = b_{0_p} + b_{1_p} * r. \qquad \text{(Equation 12)}$$

Here, r are the vectors of observed or predicted responses, b_0 is an intercept term, and b_1 is a slope term.

In the original experiment, new faces were then generated by multiplying the slopes obtained from the regressions with different "amplification values." The resulting faces had then been presented to the participants to titrate the "amplification" of the weights that would result in the highest perceptual similarity of the reconstructed face for each participant. An amplification of 0 here corresponds to the shape or texture feature being reconstructed as a function of the intercept term only. This corresponds to the shape or texture feature resulting from the average of the faces chosen from the array of six faces in the first stage of each trial.

We repeated this for the forward models by storing the shape and texture components and by rendering observable images of faces corresponding to amplification values ranging from 0 to 50 (the same range used to titrate the human reconstructions) in steps of 0.5. We then computed forward model predictions from GMF shape and texture features, and propagated the observable images through encoding models based on DNNs. This resulted in responses of all systems across the range of amplification values. We chose the peak of each curve and reconstructed the internal templates corresponding to the shape and texture components at these peaks.

We rendered the corresponding internal templates as intuitively visualizable faces. We also considered the explicit descriptions in vertex space to compare templates from humans and templates from forward models among each other, and with the ground truth face shape from the target colleagues. To evaluate the "humanness" of the forward models, we computed the Euclidean distances and correlations from the internal templates of the forward models with the internal templates of the humans. To also evaluate the "veridicality," we computed the Euclidean distances and correlations from the ground truth target colleagues with the internal templates from the forward models and the human participants.

This resulted in Euclidean distances and correlations for each target colleague condition j and human participant i (observed and predicted by different predictor spaces f). We then log-transformed the Euclidean distances and Fisher z-transformed the correlations to obtain evaluation measures e and modeled them with Bayesian hierarchical models similar to the ones used to model the prediction performances of the forward models:

$$e_n \sim N(\mu_n, \sigma^2), \qquad \text{(Equation 13)}$$

$$\sigma \sim |t(3, 0, 10)|,$$

$$\mu_n \sim \beta_{i:f[n]} + \beta_{i:j[n]} + \beta_{f_1[n]} + \ldots + \beta_{f_q[n]},$$

$$(\beta_{i:f}, \beta_{i:j}) \sim N\left(0, \ \sigma^2_{\beta_{int}}\right),$$

$$\sigma^2_{\beta_{int}} \sim |t(3, 0, 10)|,$$

$$\left(\beta_{f_1}, \ ..., \beta_{f_q}\right) \sim N(0, \ 10).$$

To compare the resulting posterior distributions of the parameters of interest, we evaluated the corresponding hypotheses using the brms package—$\beta_{f_a} - \beta_{f_b} > 0$ for all possible pairwise combinations of predictor spaces—and obtained the proportion of samples of the posterior distributions of differences that were in favor of the corresponding hypotheses. Prior to visualization, we back-transformed the posterior distributions of the log Euclidean distances with an exponential and the posterior distributions of correlations with the inverse Fisher z-transformation.

Generalization testing

The models of human behavior had been trained and tested under the same conditions. To also test how they would perform under data from a different distribution, we re-used data from a validation experiment originally conducted by Zhan and colleagues[26] .

We propagated the 50 stimulus images per target colleague (combinations of two levels of diagnosticity at five levels of amplification, which were each rendered in five different generalization conditions, see "experiments—generalization testing") through each of the model systems under consideration and extracted the rating predictions for each of the 14 participants of the first experiment for each of the four colleagues from each of the four correspondingly fitted forward models. Next, we normalized the predictions to values between 0 and 1 within target colleagues to eliminate possible biases from participants rating the random stimuli of the first experiment higher for one target colleague than for others. We then used the maximum predicted rating across all target colleagues for a given stimulus as the choice of the respective system. The predictions for each of the 14 participants of the first experiment were compared with the behavior of each of the 12 additional participants of the second experiment.

Since all systems were deterministic, the resulting accuracy values for the systems were thus binary (this was different for the human responses, since each stimulus had been shown to the validators five times; see "experiments—generalization testing").

We analyzed the data by first computing the absolute difference of human and model accuracies and then subjecting the resulting absolute errors to a Bayesian linear model. Since the model accuracies could only take one of six different values (from 0 to 1 in steps of 0.2), we used an ordinal model. To do so, we used a cumulative model assuming a normally distributed latent variable as implemented in brms.[119] Concretely, we modeled the probability of a model accuracy a of model type f predicting behavior in task g of participant i for target colleague j and validated by validator k to fall into category t given the linear predictor η as:

$$\Pr(a = \mathrm{t}|\eta) = F(\tau_t - \eta) - F(\tau_{t-1} - \eta), \qquad \text{(Equation 14)}$$

where F is a cumulative distribution function, τ_t is one of $T = 5$ different thresholds that partition the standard Gaussian continuous latent variable $\tilde{a}$ into $T + 1$ categories, and η describes $\tilde{a}$ corresponding to the following model:

$$\tau_t \sim t(3, 0, 10), \qquad \text{(Equation 15)}$$

$$\tilde{a}_n \sim N(\mu_n, \ 1),$$

$$\mu_n \sim \beta_{f:g[n]} + \beta_{i:j:k[n]},$$

$$(\beta_{f:g}, \beta_{i:j:k}) \sim N\left(0, \ \sigma^2_{\beta_{int}}\right),$$

$$\sigma^2_{\beta_{int}} \sim |t(3, 0, 10)|,$$

$$\left(\beta_{f_1}, \ ..., \beta_{f_q}\right) \sim N(0, \ 10).$$

To compare the resulting posterior distributions of the parameters of interest, we evaluated the corresponding hypotheses using the brms package ($\beta_{f_a:g_x} - \beta_{f_b:g_x} > 0$ for all possible pairwise combinations of model types within each task), and obtained the proportion of samples of the posterior distributions of differences that were in favor of the corresponding hypotheses.

CellPress
OPEN ACCESS

SUPPLEMENTAL INFORMATION

Supplemental information can be found online at https://doi.org/10.1016/j.patter.2021.100348.

ACKNOWLEDGMENTS

This work has been funded by the Wellcome Trust grant (Senior Investigator Award, UK; 107802) and the Multidisciplinary University Research Initiative/Engineering and Physical Sciences Research Council grant (USA, UK; 172046-01) awarded to P.G.S. The funders had no role in study design, data collection and analysis, decision to publish, or preparation of the manuscript.

AUTHOR CONTRIBUTIONS

C.D., J.Z., O.G.B.G., and P.G.S. designed the research; C.D. and T.X. developed the DNN models; O.G.B.G. and P.G.S. developed the GMF; R.A.A.I. developed the implementation of PID; J.Z. recorded the data; C.D. analyzed the data; C.D. and P.G.S. drafted the manuscript; C.D., J.Z., A.W., R.A.A.I., and P.G.S. revised the manuscript; P.G.S., O.G.B.G., and R.A.A.I. supervised the project; P.G.S. acquired the financial support for the project leading to this publication.

DECLARATION OF INTERESTS

The authors declare no competing interests.

Received: October 14, 2020
Revised: November 30, 2020
Accepted: August 20, 2021
Published: September 10, 2021

REFERENCES

1. Schyns, P.G., Goldstone, R.L., and Thibaut, J.-P. (1998). The development of features in object concepts. Behav. Brain Sci. *21*, 1–17.
2. DiCarlo, J.J., and Cox, D.D. (2007). Untangling invariant object recognition. Trends Cogn. Sci. *11*, 333–341.
3. Nestor, A., Lee, A.C.H., Plaut, D.C., and Behrmann, M. (2020). The face of image reconstruction: progress, pitfalls, prospects. Trends Cogn. Sci. *24*, 747–759.
4. Naselaris, T., Kay, K.N., Nishimoto, S., and Gallant, J.L. (2011). Encoding and decoding in fMRI. NeuroImage *56*, 400–410.
5. Yamins, D.L.K., Hong, H., Cadieu, C.F., Solomon, E.A., Seibert, D., and DiCarlo, J.J. (2014). Performance-optimized hierarchical models predict neural responses in higher visual cortex. Proc. Natl. Acad. Sci. U S A *111*, 8619–8624.
6. Eickenberg, M., Gramfort, A., Varoquaux, G., and Thirion, B. (2017). Seeing it all: convolutional network layers map the function of the human visual system. NeuroImage *152*, 184–194.
7. Kell, A.J.E., Yamins, D.L.K., Shook, E.N., Norman-Haignere, S.V., and McDermott, J.H. (2018). A task-optimized neural network replicates human auditory behavior, predicts brain responses, and reveals a cortical processing hierarchy. Neuron *98*, 1–15.
8. Kubilius, J., Schrimpf, M., Kar, K., Rajalingham, R., Hong, H., Majaj, N.J., et al. (2019). Brain-like object recognition with high-performing shallow recurrent ANNs. ArXiv, 1909.06161.
9. Kietzmann, T.C., Spoerer, C.J., Sörensen, L.K.A., Cichy, R.M., Hauk, O., and Kriegeskorte, N. (2019). Recurrence is required to capture the representational dynamics of the human visual system. Proc. Natl. Acad. Sci. U S A. *116*, 21854–21863.
10. Zhuang, C., Yan, S., Nayebi, A., Schrimpf, M., Frank, M.C., DiCarlo, J.J., et al. (2021). Unsupervised neural network models of the ventral visual stream. Proc. Natl. Acad. Sci. U S A. *118*. https://doi.org/10.1073/pnas.2014196118.
11. Kriegeskorte, N., and Douglas, P.K. (2018). Cognitive computational neuroscience. Nat. Neurosci. *21*, 1148–1160.
12. Szegedy, C., Zaremba, W., Sutskever, I., Bruna, J., Erhan, D., Goodfellow, I., et al. (2014). Intriguing properties of neural networks. ArXiv, 1312.6199.
13. Jacobsen, J.-H., Behrmann, J., Zemel, R., and Bethge, M. (2019). Excessive invariance causes adversarial vulnerability. ArXiv, 1811.00401.
14. Rajalingham, R., Issa, E.B., Bashivan, P., Schmidt, K., and DiCarlo, J.J. (2018). Large-scale, high-resolution comparison of the core visual object recognition behavior of humans, monkeys, and state-of-the-art deep artificial neural networks. J. Neurosci. *38*, 7255–7269.
15. Barbu, A., Mayo, D., Alverio, J., Luo, W., Wang, C., Gutfreund, D., et al. (2019). ObjectNet: a large-scale bias-controlled dataset for pushing the limits of object recognition models. Advances in Neural Information Processing Systems 32 (NeurIPS 2019). ArXiv, https://openreview.net/pdf?id=SkgnRNHgIS.
16. Geirhos, R., Jacobsen, J.H., MIchaelis, C., Zemel, R., Brendel, W., Bethge, M., et al. (2020). Shortcut learning in deep neural networks. ArXiv. 2004.07780. https://doi.org/10.1038/s42256-020-00257-z.
17. Golan, T., Raju, P.C., and Kriegeskorte, N. (2020). Controversial stimuli: pitting neural networks against each other as models of human recognition. PNAS *117*, 29330–29337.
18. Medin, D.L., Goldstone, R.L., and Gentner, D. (1993). Respects for similarity. Psychol. Rev. *100*, 254–278.
19. Edelman, S. (1995). Representation, similarity, and the chorus of prototypes. Minds Mach *5*, 45–68.
20. Schyns, P.G., and Rodet, L. (1997). Categorization creates functional features. J. Exp. Psychol. Learn. Mem. Cogn. *23*, 681–696.
21. Lapuschkin, S., Wäldchen, S., Binder, A., Montavon, G., Samek, W., and Müller, K.-R. (2019). Unmasking Clever Hans predictors and assessing what machines really learn. Nat. Commun. *10*, 1096.
22. Schyns, P.G., Jentzsch, I., Johnson, M., Schweinberger, S.R., and Gosselin, F. (2003). A principled method for determining the functionality of brain responses. Neuroreport *14*, 1665–1669.
23. Marr, D. (2010). Vision: A Computational Investigation into the Human Representation and Processing of Visual Information (MIT Press).
24. Krakauer, J.W., Ghazanfar, A.A., Gomez-Marin, A., MacIver, M.A., and Poeppel, D. (2017). Neuroscience needs behavior: correcting a reductionist bias. Neuron *93*, 480–490.
25. Kay, K.N. (2018). Principles for models of neural information processing. NeuroImage *180*, 101–109.
26. Zhan, J., Garrod, O.G.B., van Rijsbergen, N., and Schyns, P.G. (2019). Modelling face memory reveals task-generalizable representations. Nat. Hum. Behav. *3*, 817–826.
27. Murray, R.F. (2011). Classification images: a review. J. Vis. *11*, 2.
28. Schyns, P.G., Zhan, J., Jack, R.E., and Ince, R.A.A. (2020). Revealing the information contents of memory within the stimulus information representation framework. Philos. Trans. R. Soc. B Biol. Sci. *375*, 20190705.
29. Ince, R.A.A. (2017). Measuring multivariate redundant information with pointwise common change in surprisal. Entropy *19*. https://doi.org/10.3390/e19070318.
30. Daube, C., Giordano, B.L., Schyns, P., and Ince, R. (2019). Quantitatively comparing predictive models with the partial information decomposition. In 2019 Conference on Cognitive Computational Neuroscience (Cognitive Computational Neuroscience). https://doi.org/10.32470/CCN.2019.1142-0.
31. He, K., Zhang, X., Ren, S., and Sun, J. (2015). Deep residual learning for image recognition. ArXiv, 1512.03385.
32. Schroff, F., Kalenichenko, D., and Philbin, J. (2015). FaceNet: a unified embedding for face recognition and clustering. In 2015 IEEE Conference on Computer Vision and Pattern Recognition (CVPR) (IEEE), pp. 815–823. https://doi.org/10.1109/CVPR.2015.7298682.

33. Xu, T., Zhan, J., Garrod, O.G.B., Torr, P.H.S., Zhu, S.-C., Ince, R.A.A., et al. (2018). Deeper interpretability of deep networks. ArXiv, 1811.07807.

34. Ballard, D.H. (1987). Modular learning in neural networks. In Proceedings of the Sixth National Conference on Artificial Intelligence, *Volume 1* (AAAI Press), pp. 279–284.

35. Zhu, Z., Luo, P., Wang, X., and Tang, X. (2013). Deep learning identity-preserving face space. In 2013 IEEE International Conference on Computer Vision (IEEE), pp. 113–120. https://doi.org/10.1109/ICCV.2013.21.

36. Van der Maaten, L.van der, and Hinton, G. (2008). Visualizing Data using t-SNE. J. Mach. Learn. Res. *9*, 2579–2605.

37. Varoquaux, G., Raamana, P.R., Engemann, D.A., Hoyos-Idrobo, A., Schwartz, Y., and Thirion, B. (2017). Assessing and tuning brain decoders: cross-validation, caveats and guidelines. NeuroImage *145*, 166–179.

38. Bürkner, P.C. (2017). Brms: an R package for Bayesian multilevel models using stan. J. Stat. Softw. *80*. https://doi.org/10.18637/jss.v080.i01.

39. Saxe, A., Nelli, S., and Summerfield, C. (2021). If deep learning is the answer, what is the question? Nat. Rev. Neurosci. *22*, 55–67.

40. Diedrichsen, J. (2020). Representational models and the feature fallacy. In The Cognitive Neurosciences, D. Poeppel, G.R. Mangun, and M.S. Gazzaniga, eds. (MIT Press), pp. 669–678.

41. Daube, C., Ince, R.A.A., and Gross, J. (2019). Simple acoustic features can explain phoneme-based predictions of cortical responses to speech. Curr. Biol. *29*, 1924–1937.e9.

42. Schyns, P.G., Gosselin, F., and Smith, M.L. (2009). Information processing algorithms in the brain. Trends Cogn. Sci. *13*, 20–26.

43. Chaitin, G.J. (1975). A theory of program size formally identical to information theory. J. Assoc. Comput. Mach *22*, 329–340.

44. Morgan, C.L. (2018). An Introduction to Comparative Psychology (Forgotten Books).

45. Buckner, C. (2019). The Comparative Psychology of Artificial Intelligences (Philsci-Archive). pitt.edu/16034/.

46. Kubilius, J. (2018). Predict, then simplify. NeuroImage *180*, 110–111.

47. Olman, C., and Kersten, D. (2004). Classification objects, ideal observers & generative models. Cogn. Sci. *28*, 227–239.

48. Greene, M.R., Botros, A.P., Beck, D.M., and Fei-Fei, L. (2014). Visual noise from natural scene statistics reveals human scene category representations. ArXiv, 1411.5331.

49. Lescroart, M.D., and Gallant, J.L. (2019). Human scene-selective areas represent 3D configurations of surfaces. Neuron *101*, 178–192.e7.

50. Jack, R.E., and Schyns, P.G. (2017). Toward a social psychophysics of face communication. Annu. Rev. Psychol. *68*, 269–297.

51. Grenander, U. (1994). General Pattern Theory: A Mathematical Study of Regular Structures (Oxford University Press).

52. Yuille, A., and Kersten, D. (2006). Vision as Bayesian inference: analysis by synthesis? Trends Cogn. Sci. *10*, 301–308.

53. Yildirim, I., Belledonne, M., Freiwald, W., and Tenenbaum, J. (2020). Efficient inverse graphics in biological face processing. Sci. Adv. *6*, eaax5979.

54. Kubilius, J., Bracci, S., and Op de Beeck, H.P. (2016). Deep neural networks as a computational model for human shape sensitivity. PLoS Comput. Biol. *12*, e1004896.

55. Baker, N., Lu, H., Erlikhman, G., and Kellman, P.J. (2018). Deep convolutional networks do not classify based on global object shape. PLoS Comput. Biol. *14*, e1006613.

56. Geirhos, R., Rubisch, P., Michaelis, C., Bethge, M., Wichmann, F.A., and Brendel, W. (2019). ImageNet-trained CNNs are biased towards texture; increasing shape bias improves accuracy and robustness. ArXiv, 1811.12231.

57. Brendel, W., and Bethge, M. (2019). Approximating CNNs with Bag-of-local-Features models works surprisingly well on ImageNet. ArXiv, 1904.00760.

58. Hermann, K.L., and Kornblith, S. (2019). Exploring the origins and prevalence of texture bias in convolutional neural networks. ArXiv, 1911.09071.

59. Doerig, A., Schmittwilken, L., Sayim, B., Manassi, M., and Herzog, M.H. (2020). Capsule networks as recurrent models of grouping and segmentation. PLoS Comput. Biol. *16*, e1008017.

60. Chang, L., Egger, B., Vetter, T., and Tsao, D.Y. (2021). Explaining face representation in the primate brain using different computational models. Curr. Biol. *31*, 2785–2795.e4.

61. Jozwik, K.M., O'Keeffe, J., Storrs, K.R., and Kriegeskorte, N. (2021). Face dissimilarity judgements are predicted by representational distance in deep neural networks and principal-component face space. bioRxiv. https://doi.org/10.1101/2021.04.09.438859.

62. VanRullen, R., and Reddy, L. (2019). Reconstructing faces from fMRI patterns using deep generative neural networks. Commun. Biol. *2*, 193.

63. Bashivan, P., Kar, K., and DiCarlo, J.J. (2019). Neural population control via deep image synthesis. Science *364*, eaav9436.

64. Ponce, C.R., Xiao, W., Schade, P.F., Hartmann, T.S., Kreimann, G., and Livingstone, M.S. (2019). Evolving images for visual neurons using a deep generative network reveals coding principles and neuronal preferences. Cell *177*, 999–1009.e10.

65. Karras, T., Laine, S., Aittala, M., Hellsten, J., Lehtinen, J., Aila, T., et al. (2020). Analyzing and improving the image quality of StyleGAN. ArXiv, 1912.04958.

66. van den Oord, A., Vinyals, O., and Kavukcuoglu, K. (2018). Neural discrete representation learning. ArXiv, 1711.00937.

67. Razavi, A., Oord, A.van den, and Vinyals, O. (2019). Generating diverse high-fidelity images with VQ-VAE-2. ArXiv, 1906.00446.

68. Vahdat, A., and Kautz, J. (2020). NVAE: a deep hierarchical variational autoencoder. ArXiv, 2007.03898.

69. Suchow, J.W., Peterson, J.C., and Griffiths, T.L. (2018). Learning a face space for experiments on human identity. ArXiv, 1805.07653.

70. Bontrager, P., Lin, W., Togelius, J., and Risi, S. (2018). Deep interactive evolution. ArXiv, 1801.08230.

71. Todorov, A., Uddenberg, S., Peterson, J., Griffiths, T., and Suchow, J. (2020). Data-Driven, Photorealistic Social Face-Trait Encoding, Prediction, and Manipulation Using Deep Neural Networks (Princeton University).

72. Goetschalckx, L., Andonian, A., and Wagemans, J. (2021). Generative adversarial networks unlock new methods for cognitive science. Trends Cogn. Sci. *25*, 788–801.

73. Peterson, J., Uddenberg, S., Griffiths, T., Todorov, A., and Suchow, J. (2021). Capturing and modifying the perceived traits of all possible faces. PsyArxiv. https://doi.org/10.31234/osf.io/brzfy.

74. Mathieu, E., Rainforth, T., Siddharth, N., and Teh, Y.W. (2019). Disentangling disentanglement in variational autoencoders. ArXiv, 1812.02833.

75. Schölkopf, B., Locatello, F., Bauer, S., Ke, N.R., Kalchbrenner, N., Goyal, A., et al. (2021). Toward causal representation learning. Proc. IEEE. *109*, 612–634.

76. Hoel, E. (2021). The overfitted brain: dreams evolved to assist generalization. Patterns *2*, 100244.

77. Scholte, H.S., Losch, M.M., Ramakrishnan, K., de Haan, E.H.F., and Bohte, S.M. (2018). Visual pathways from the perspective of cost functions and multi-task deep neural networks. Cortex *98*, 249–261.

78. Standley, T., Zamir, A.R., Chen, D., Guibas, L., Malik, J., and Savarese, S. (2020). Which tasks should be learned together in multi-task learning? ArXiv, 1905.07553.

79. Mao, C., Gupta, A., Nitin, V., Ray, B., Song, S., Yang, J., et al. (2020). Multitask learning strengthens adversarial robustness. ArXiv, 2007.07236.

80. Schott, L., Rauber, J., Bethge, M., and Brendel, W. (2018). Towards the first adversarially robust neural network model on MNIST. ArXiv, 1805.09190.

CellPress
OPEN ACCESS

81. Blauch, N.M., Behrmann, M., and Plaut, D.C. (2020). Computational insights into human perceptual expertise for familiar and unfamiliar face recognition. Cognition, 104341. https://doi.org/10.1016/j.cognition.2020.104341.

82. Fabius, J.H., Fracasso, A., Nijboer, T.C.W., and Stigchel, S.V.der. (2019). Time course of spatiotopic updating across saccades. Proc. Natl. Acad. Sci. U S A *116*, 2027–2032.

83. Friston, K., Adams, R., Perrinet, L., and Breakspear, M. (2012). Perceptions as hypotheses: saccades as experiments. Front. Psychol. *3*. https://doi.org/10.3389/fpsyg.2012.00151.

84. Ince, R.A.A., Jaworska, K., Gross, J., Panzeri, S., van Rijsbergen, N.J., Rousselet, G.A., et al. (2016). The deceptively simple N170 reflects network information processing mechanisms involving visual feature coding and transfer across hemispheres. Cereb. Cortex *26*, 4123–4135.

85. Zhan, J., Ince, R.A.A., van Rijsbergen, N., and Schyns, P.G. (2019). Dynamic construction of reduced representations in the brain for perceptual decision behavior. Curr. Biol. *29*, 319–326.e4.

86. Smith, M.L., Gosselin, F., and Schyns, P.G. (2012). Measuring internal representations from behavioral and brain data. Curr. Biol. *22*, 191–196.

87. Smith, E.E., and Sloman, S.A. (1994). Similarity- versus rule-based categorization. Mem. Cognit. *22*, 377–386.

88. Griffiths, D.W., Blunden, A.G., and Little, D.R. (2017). 12—logical-rule based models of categorization: using systems factorial technology to understand feature and dimensional processing. In Systems Factorial Technology, D.R. Little, N. Altieri, M. Fifić, and C.-T. Yang, eds. (Academic Press). https://doi.org/10.1016/B978-0-12-804315-8.00015-X.

89. Chang, L., and Tsao, D.Y. (2017). The code for facial identity in the primate brain. Cell *169*, 1013–1028.e14.

90. Zeiler, M.D., and Fergus, R. (2013). Visualizing and understanding convolutional networks. ArXiv, 1311.2901.

91. Zhou, B., Khosla, A., Lapedriza, A., Oliva, A., and Torralba, A. (2015). Learning deep features for discriminative localization. ArXiv, 1512.04150.

92. Erhan, D., Bengio, Y., Courville, A., Vincent, P., and Box, P.O. (2009). Visualizing higher-layer features of a deep network. Techreport 1341 (University of Montreal).

93. Simonyan, K., Vedaldi, A., and Zisserman, A. (2014). Deep inside convolutional networks: visualising image classification models and saliency maps. ArXiv, 1312.6034.

94. Olah, C., Cammarata, N., Schubert, L., Goh, G., Petrov, M., and Carter, S. (2020). Zoom in: an introduction to circuits. Distill *5*, e00024.001.

95. Olah, C., Satyanarayan, A., Johnson, I., Carter, S., Schubert, L., Ye, K., et al. (2018). The building blocks of interpretability. Distill *3*, e10.

96. Olah, C., Mordvintsev, A., and Schubert, L. (2017). Feature visualization. Distill *2*, e7.

97. Keshishian, M., Akbari, H., Khalighinejad, B., Herrero, J.L., Mehta, A.D., and Mesgarani, N. (2020). Estimating and interpreting nonlinear receptive field of sensory neural responses with deep neural network models. eLife *9*, e53445.

98. Bach, S., Binder, A., Montavon, G., Klauschen, F., Müller, K.-R., and Samek, W. (2015). On pixel-wise explanations for non-linear classifier decisions by layer-wise relevance propagation. PLoS One *10*, e0130140.

99. Montavon, G., Samek, W., and Müller, K.-R. (2018). Methods for interpreting and understanding deep neural networks. Digit. Signal. Process. *73*, 1–15.

100. Funke, C.M., Borowski, J., Stosio, K., Brendel, W., Wallis, T.S.A., and Bethge, M. (2021). Five points to check when comparing visual perception in humans and machines. J. Vis. *21*. https://doi.org/10.1167/jov.21.3.16.

101. Thoret, E., Andrillon, T., Léger, D., and Pressnitzer, D. (2021). Probing machine-learning classifiers using noise, bubbles, and reverse correlation. J. Neurosci. Methods *362*, 109297.

102. Gan, C., Schwartz, J., Alter, S., Schrimpf, M., Traer, J., De Freitas, J., et al. (2020). ThreeDWorld: a platform for interactive multi-modal physical simulation. ArXiv, 2007.04954.

103. DI4D. DI4D. https://di4d.com/.

104. Chollet, F., et al. (2015). keras. https://github.com/fchollet/keras.

105. Abadi, M., Barham, P., Chen, J., Chen, Z., Davis, A., Dean, J., et al. (2016). TensorFlow: a system for large-scale machine learning. In 12th USENIX Symposium on Operating Systems Design and Implementation (OSDI 16) (USENIX Association), pp. 265–283.

106. Zhang, S., Huang, J.-B., Lim, J., Gong, Y., Wang, J., Ahuja, N., et al. (2017). Tracking persons-of-interest via unsupervised representation adaptation. ArXiv, 1710.02139.

107. Codella, N. (2020). tripletloss-keras-tensorflow. https://github.com/noelcodella/tripletloss-keras-tensorflow.

108. Smith, L.N. (2017). Cyclical learning rates for training neural networks. ArXiv, 1506.01186.

109. Graves, A. (2020). BVEA-tf. https://github.com/alecGraves/BVAE-tf.

110. Kingma, D.P., and Ba, J. (2017). Adam: a method for stochastic optimization. ArXiv, 1412.6980.

111. Kingma, D.P., and Welling, M. (2014). Auto-encoding variational Bayes. ArXiv, 1312.6114.

112. Higgins, I., Matthey, L., Pal, A., Burgess, P., Glorot, X., Botvinick, M., et al. (2016). Beta-VAE: Learning Basic Visual Concepts with a Constrained Variational Framework (ICLR).

113. Acerbi, L., and Ma, W.J. (2017). Practical Bayesian optimization for model fitting with Bayesian adaptive direct search. Adv. Neural Inf. Process. Syst. *30*, 1834–1844.

114. Ince, R.A.A., Giordano, B.L., Kayser, Christoph, Rousselet, G.A., Gross, J., and Schyns, P.G. (2017). A statistical framework for neuroimaging data analysis based on mutual information estimated via a Gaussian copula. Hum. Brain Mapp. *38*, 1541–1573.

115. R Core Team (2013). R: A Language and Environment for Statistical Computing (R Foundation for Statistical Computing).

116. Stan Development Team (2018). RStan: The R Interface to Stan. https://cran.r-project.org/web/packages/rstan/vignettes/rstan.html.

117. Haufe, S., Meinecke, F., Görgen, K., Dähne, S., Haynes, J.-D., Blankertz, B., et al. (2014). On the interpretation of weight vectors of linear models in multivariate neuroimaging. NeuroImage *87*, 96–110.

118. van Vliet, M., and Salmelin, R. (2020). Post-hoc modification of linear models: combining machine learning with domain information to make solid inferences from noisy data. NeuroImage *204*, 116221.

119. Bürkner. P.-C., Vuorre M. Ordinal regression models in Psychology: a tutorial. Adv. Methods Pract. Psychol. Sci. 1:77–101.

Patterns, Volume *2*

Supplemental information

Grounding deep neural network predictions of human categorization behavior in understandable functional features: The case of face identity

Christoph Daube, Tian Xu, Jiayu Zhan, Andrew Webb, Robin A.A. Ince, Oliver G.B. Garrod, and Philippe G. Schyns

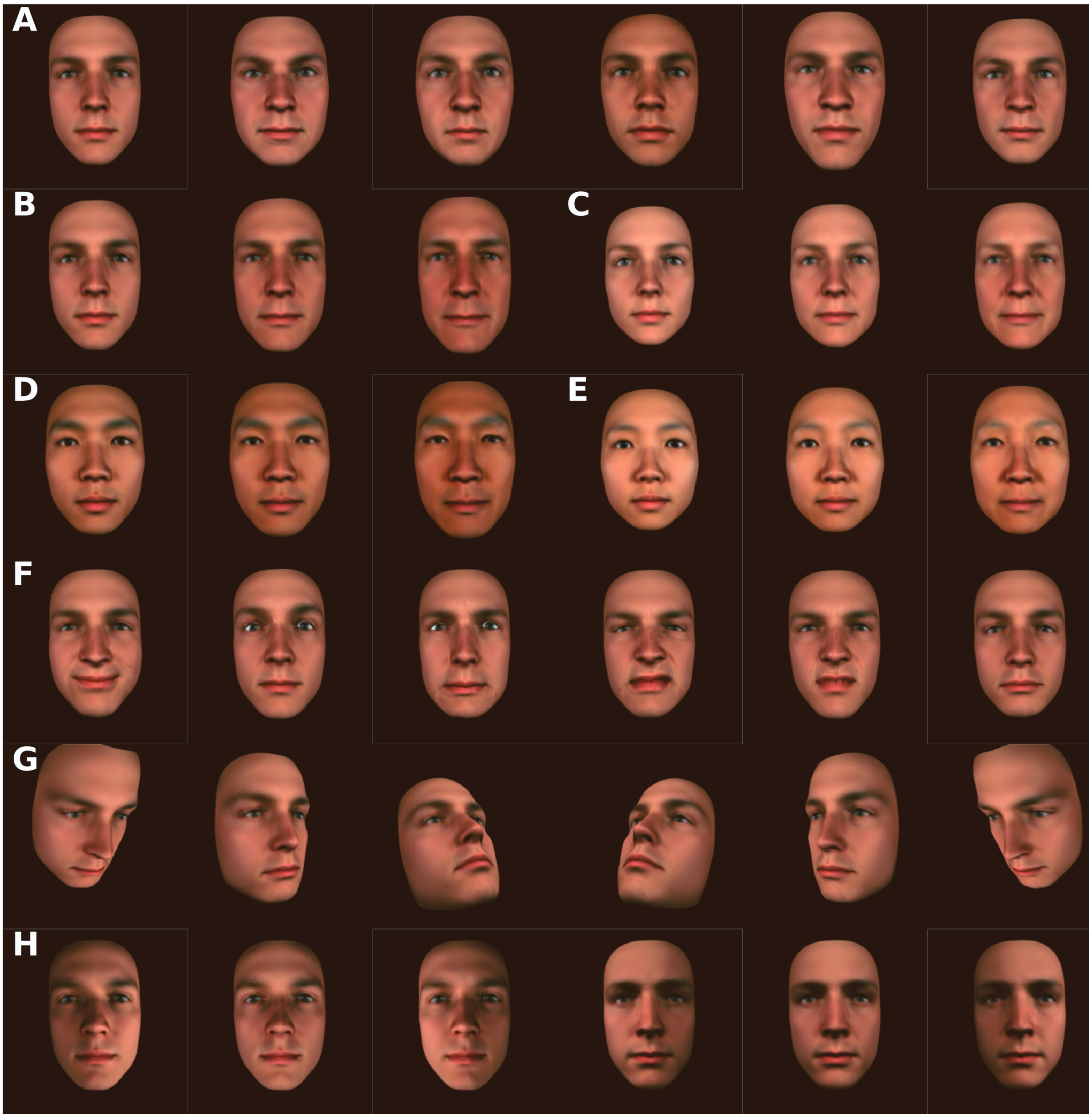

Figure S1: Demonstration of GMF variations used for training set of DNNs (related to Figure 2). **A** Six different example identities. **B** First identity from A rendered in three different ages. **C – E** Same as in B, but rendered with different sex and ethnicity. **F** First identity from A rendered with 6 additional expressions. **G** First identity from A rendered with different viewing angles. **H** First identity from A rendered with different lighting angles.

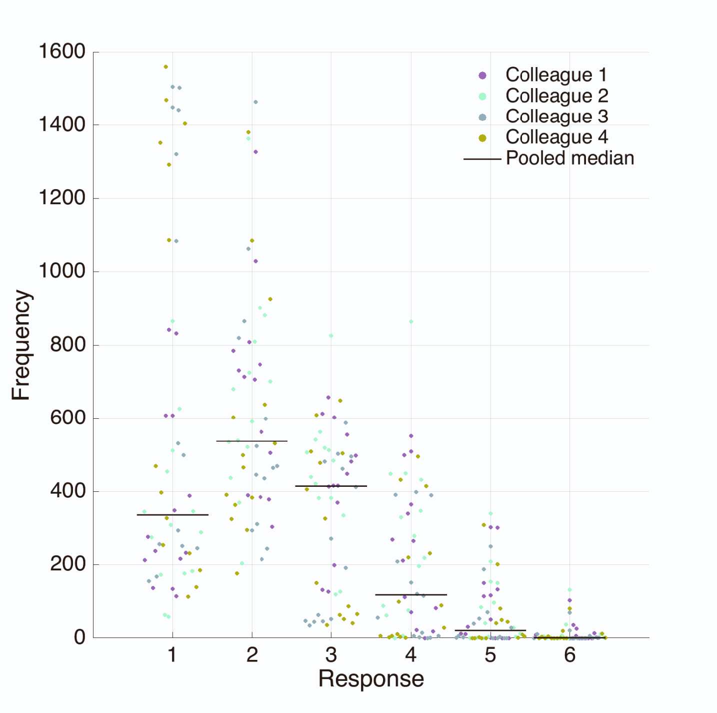

Figure S2: Distribution of rating responses in the human reverse correlation experiment (related to Figure 3).
1 codes for low similarity, 6 codes for highest similarity of stimulus to familiar target identity. Each data point represents the combination of one participant and one target familiar identity.

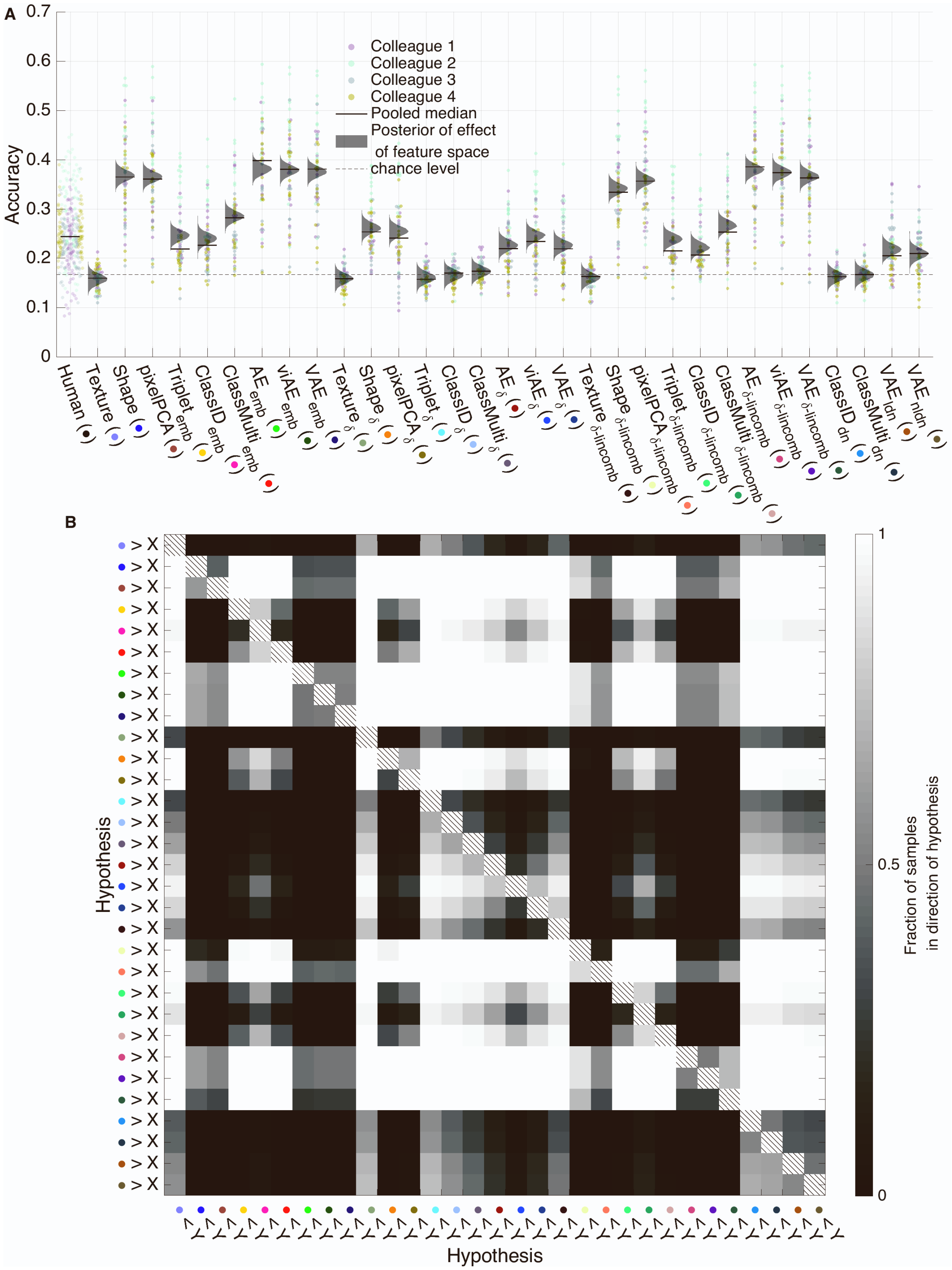

Figure S3: Accuracy of forward models in predicting choice behavior (related to Figure 3).
A Choice accuracy. On each trial, humans were presented with an array of 6 different random faces. They were asked to choose the one that most resembled the respective target colleague prior to reporting the perceived similarity on a 6-point rating scale. On each trial, the forward models "chose" the face of the array of 6 that had the highest rating among all faces of the array. The panel shows how well each model's choices matched the choices of the human participants. Pairwise matches of human participants with each other are displayed for reference. See figure S4 for explanation of the model shorthands. **B** Comparisons of the posterior distributions of the main effects for all forward models from Bayesian linear modeling of the raw performances. For each pair in the matrices, the color gradient reflects the fraction of samples of the system color coded on the y-axis that is larger than the system color coded on the x-axis. See x-axis labels for color legend.

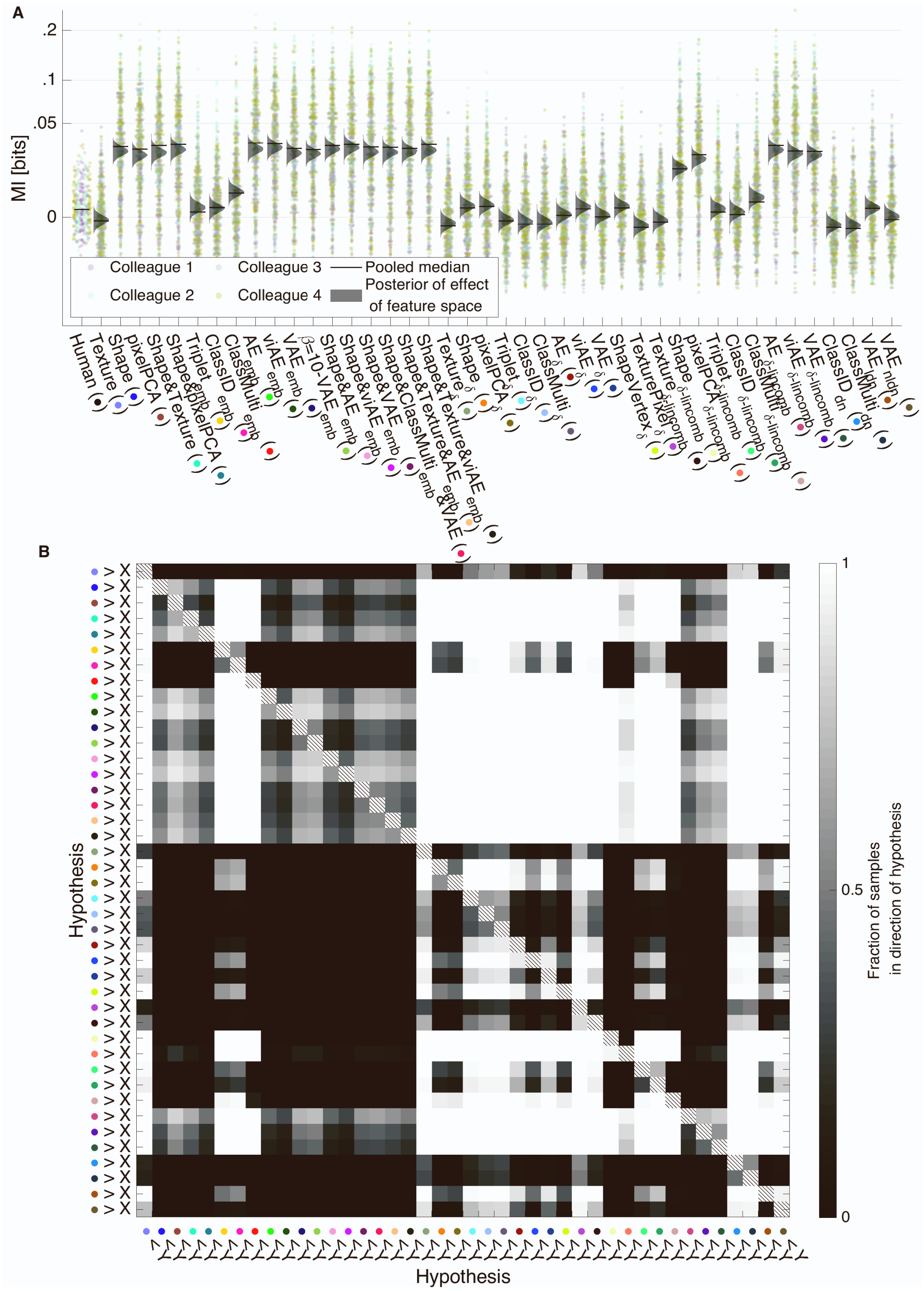

Figure S4: Bivariate evaluations of a larger set of encoding models (related to Figure 3).
A Mutual Information (MI) of observed behavior and test-set predictions from GMF features and various functions of DNN activations as well as human participants predicting other human participants (pairwise comparisons). Models include variational autoencoders ("VAE",[1]), VAEs with regularization ("β-VAE",[2]), euclidean distances of representations of the ground truth colleagues and the respective trials ("δ"), weighted euclidean distances ("δ-lincomb"), pre-softmax decision neuron activity ("logits") of the respective colleagues of ClassID and ClassMulti networks ("dn") as well as of ID classifiers trained on top of frozen VAE encoder networks (linear, "VAE$_{ldn}$", and with 2 rectified fully connected layers of 512 neurons ("VAE$_{nldn}$"). **B** Comparisons of the posterior distributions of the main effects for all systems from Bayesian linear modeling of the raw performances. For each pair in the matrices, the color gradient reflects the fraction of samples of the forward model color coded on the y-axis that is larger than the forward model color coded on the x-axis. See x-axis labels for color legend.

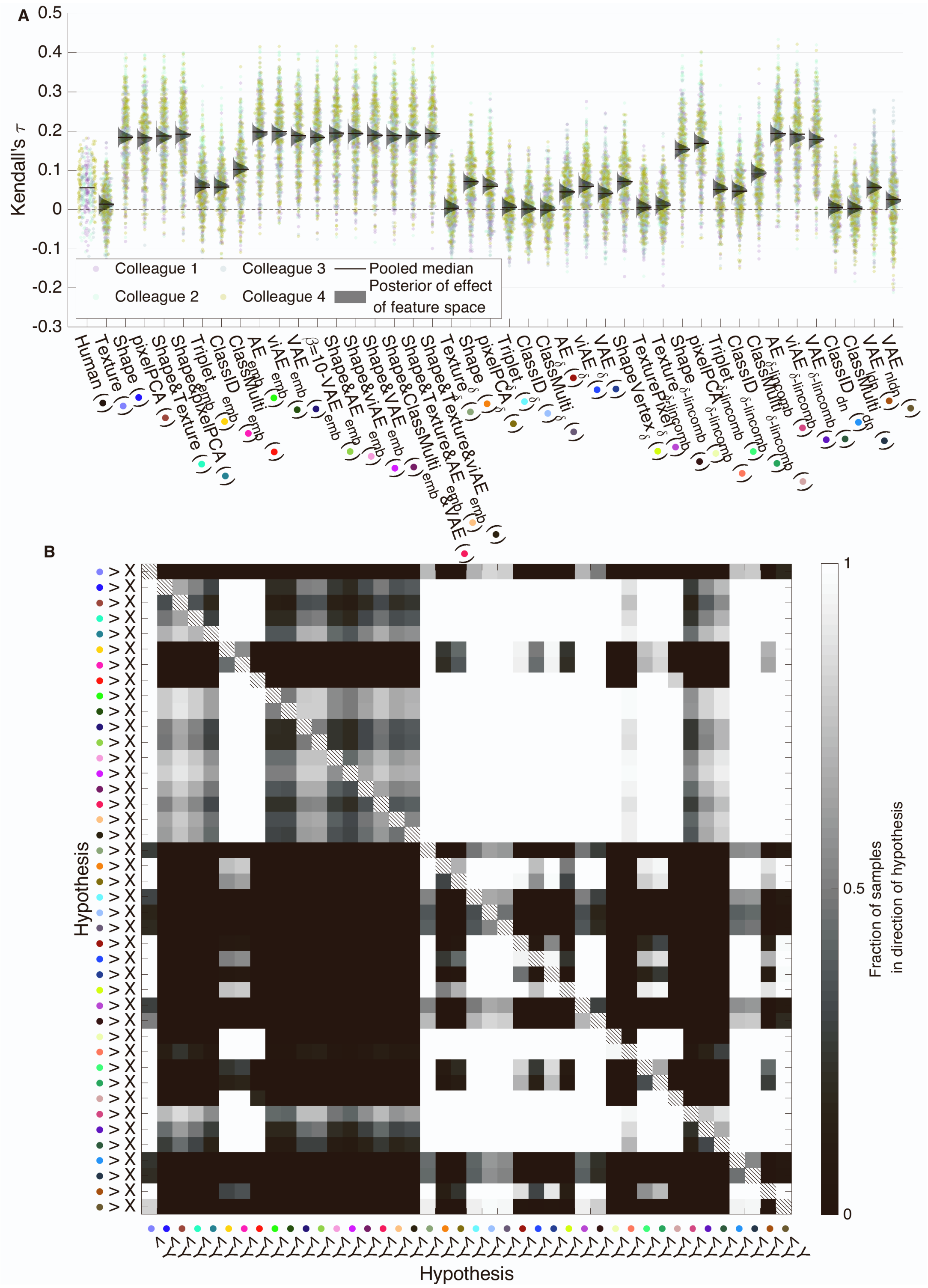

Figure S5: Bivariate evaluations of a larger set of encoding models (related to Figure 3).
A Kendall's τ of observed behavior and test-set predictions from GMF features and DNN activations as well as human participants predicting other human participants (pairwise comparisons). Except for the different metric, the analysis of this figure is identical to figure S4. See figure S4 for explanation of the model shorthands. **B** Comparisons of the posterior distributions of the main effects for all systems from Bayesian linear modeling of the raw performances. For each pair in the matrices, the color gradient reflects the fraction of samples of the forward model color coded on the y-axis that is larger than the forward model color coded on the x-axis. See x-axis labels for color legend.

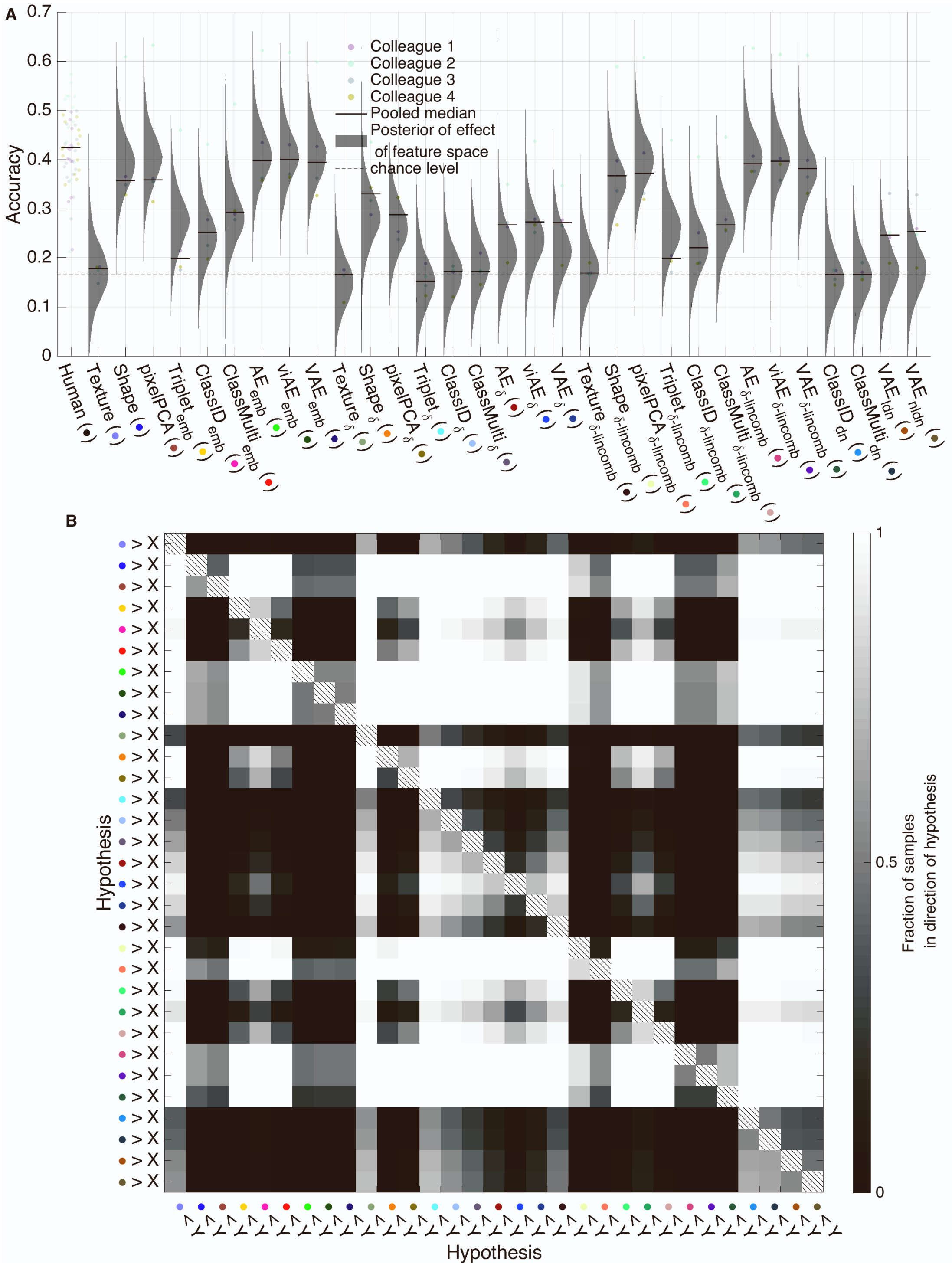

Figure S6: Accuracy of forward models in predicting choice behavior consensus across participants (related to Figure 3).
A Choice accuracy. Instead of predicting the behavior of individual human participants as in figure S3, here, for each panel of 6 faces per trial, the option chosen by the highest number of participants was used to represent the consensus across participants. See figure S4 for explanation of the model shorthands.
B Comparisons of the posterior distributions of the main effects for all systems from Bayesian linear modeling of the raw performances. For each pair in the matrices, the color gradient reflects the fraction of samples of the forward model color coded on the y-axis that is larger than the forward model color coded on the x-axis. See x-axis labels for color legend.

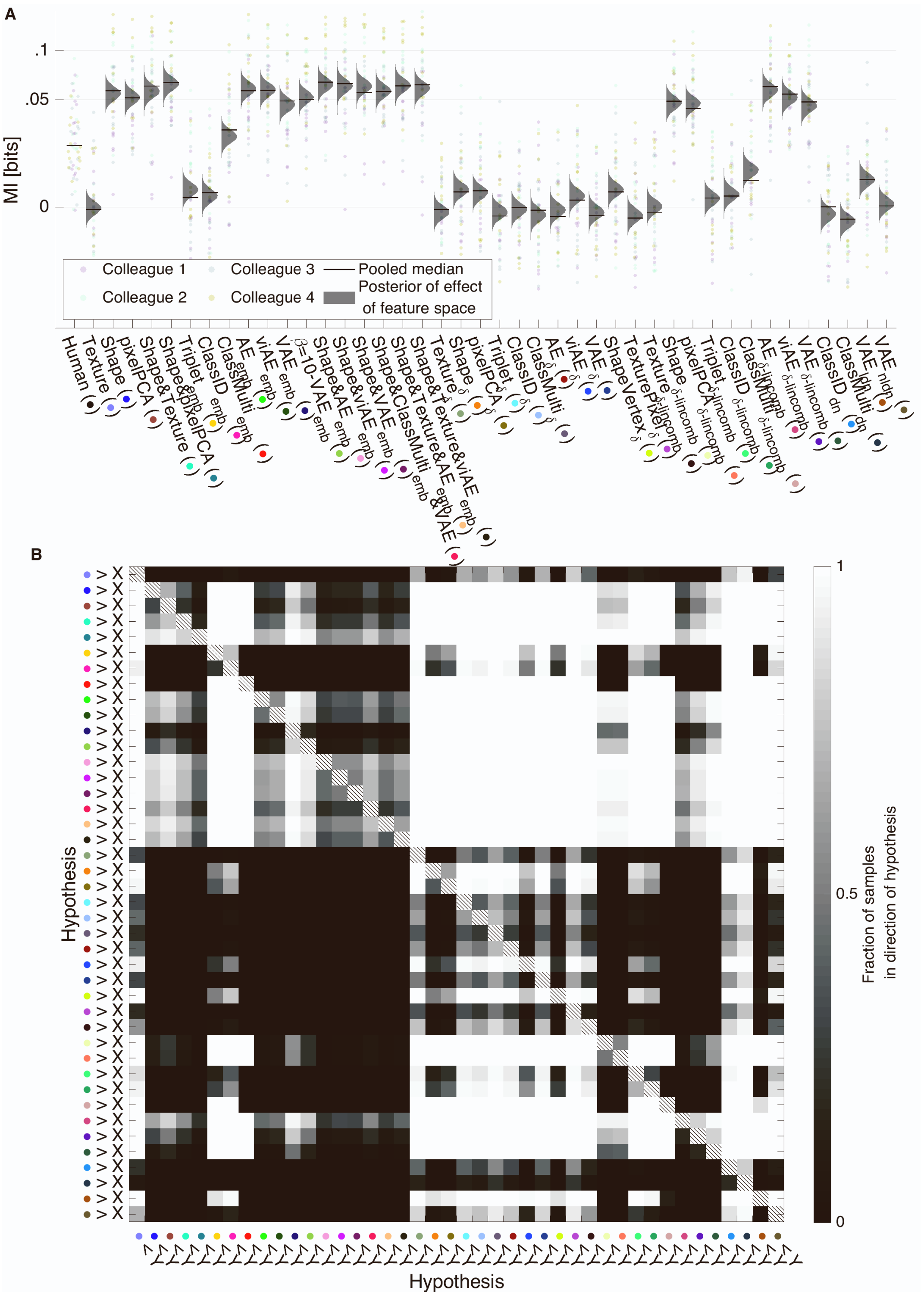

Figure S7: Bivariate evaluations of a larger set of encoding models on ratings averaged across participants (related to Figure 3).
A Mutual Information of averaged behavior and test-set predictions from GMF features and DNN activations as well as human participants predicting other human participants. Except for the different predictee, the analysis of this figure is identical to figure S4. See figure S4 for explanation of the model shorthands. **B** Comparisons of the posterior distributions of the main effects for all systems from Bayesian linear modeling of the raw performances. For each pair in the matrices, the color gradient reflects the fraction of samples of the forward model color coded on the y-axis that is larger than the forward model color coded on the x-axis. See x-axis labels for color legend.

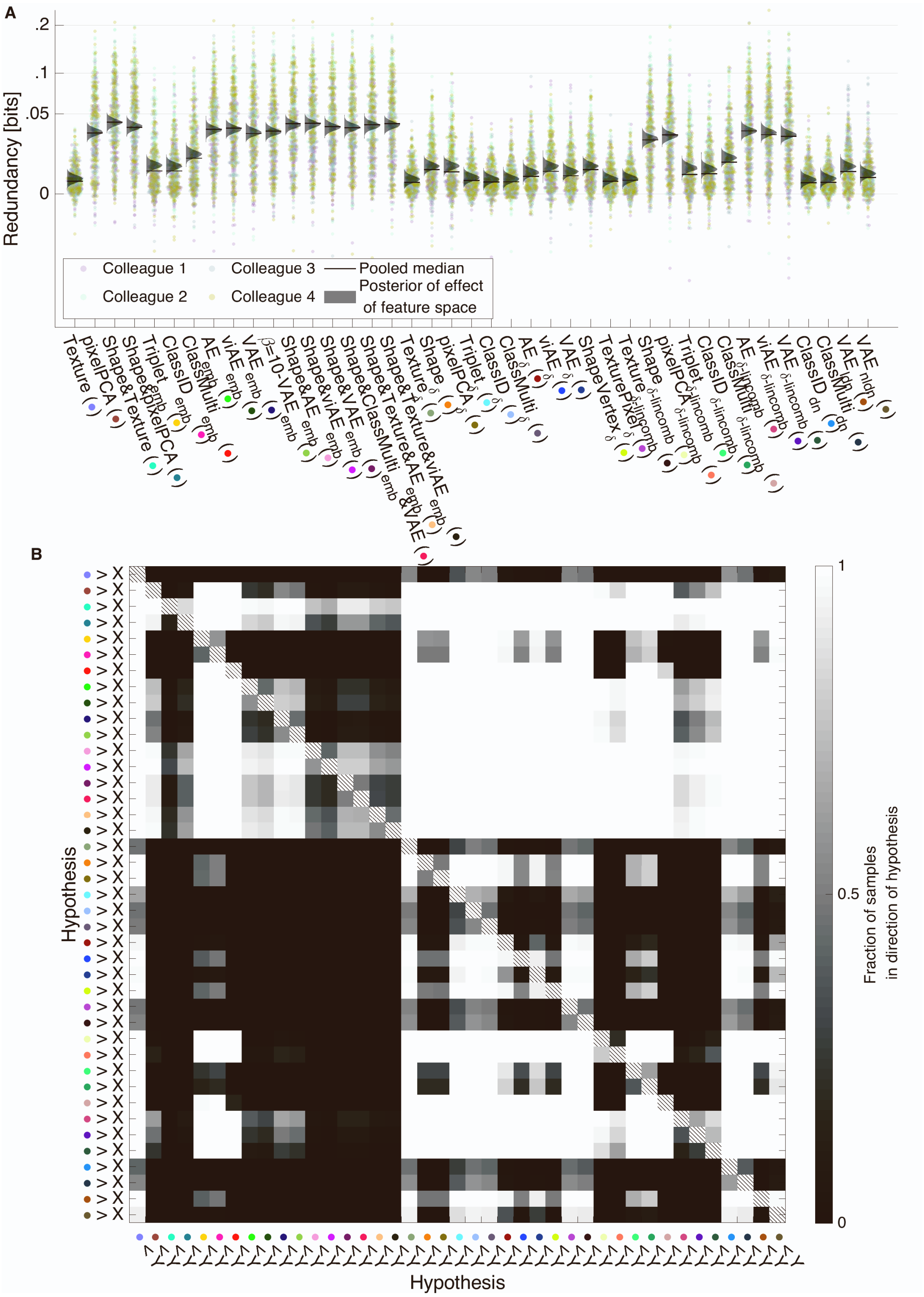

Figure S8: Redundancy with shape of a larger set of encoding models (related to Figure 3).
A Redundant information about human behavior that is shared between model predictions and GMF shape feature predictions. See figure S4 for explanation of the model shorthands. **B** Comparisons of the posterior distributions of the main effects for all systems from Bayesian linear modeling of the raw redundancies. For each pair in the matrices, the color gradient reflects the fraction of samples of the forward model color coded on the y-axis that is larger than the forward model color coded on the x-axis. See x-axis labels for color legend.

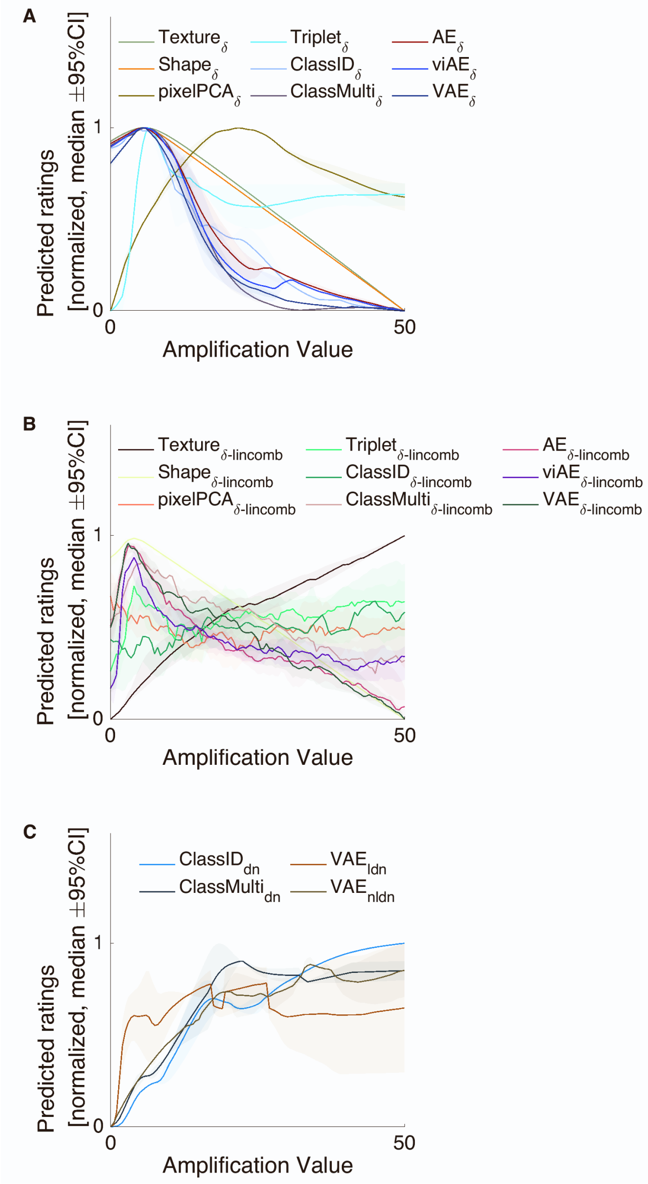

Figure S9: Amplification tuning responses of additional encoding models (related to Figure 4). **A** Amplification tuning responses of euclidean distances ("δ") of templates amplified at different levels and ground truth representations of the target colleagues. Solid lines denote the pooled median across participants and target colleagues, shaded regions denote 95% (frequentist) confidence intervals bootstrapped using $10,000$ samples. **B** Same as in A, but showing amplification tuning responses of linearly weighted euclidean distances instead ("δ-lincomb"). **C** Same as in A, but showing amplification tuning responses of pre-softmax decision neuron activities ("logits") of respective target colleagues instead ("dn").

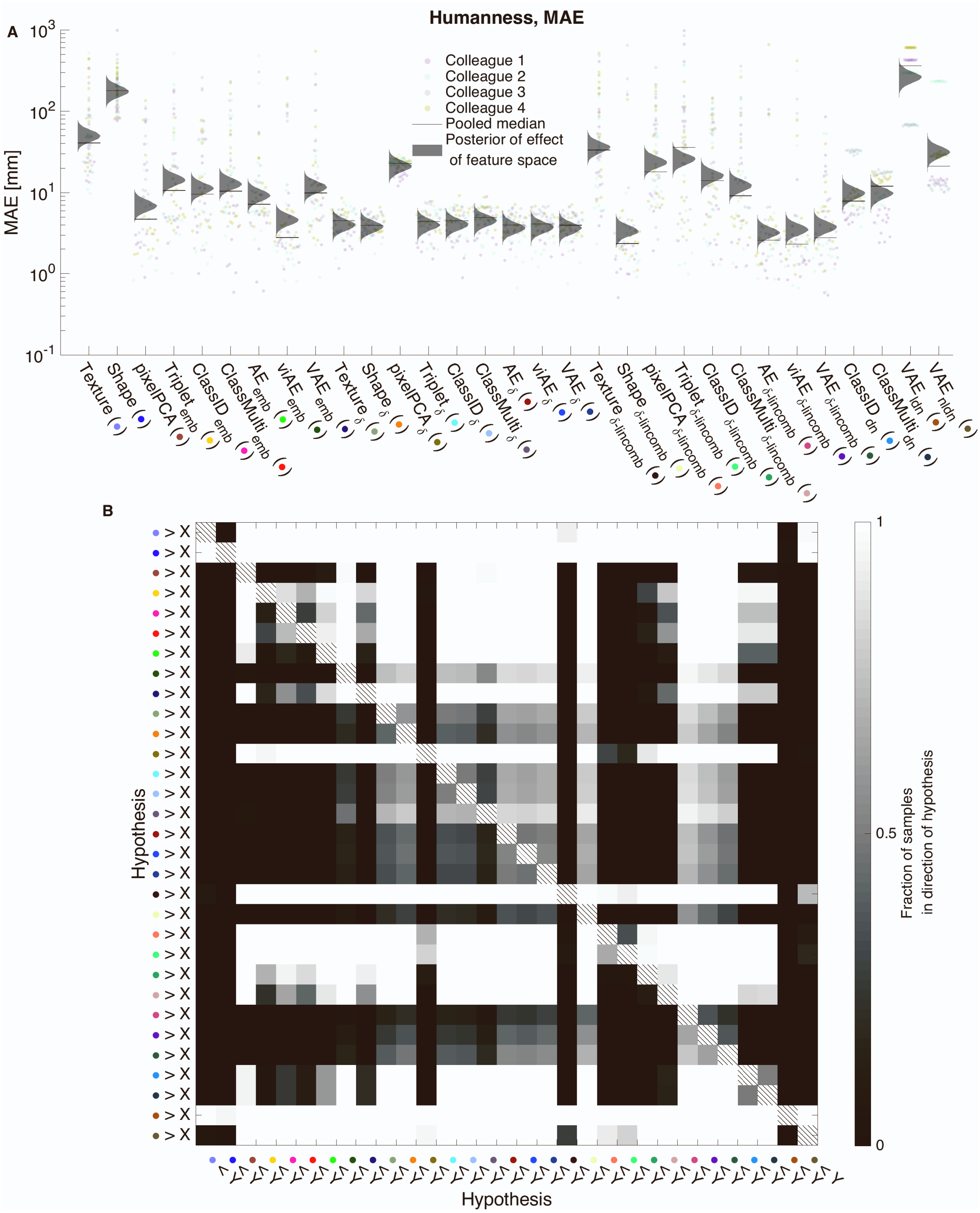

Figure S10: Evaluation of the mean absolute error between reverse-correlated faces of humans and reverse-correlated faces of models for a larger set of encoding models (related to Figure 4). **A** Mean absolute error (MAE, computed as the euclidean distances in 3D space averaged across vertices) of reverse correlated templates of the models and those of humans. See figure S4 for explanation of the model shorthands. **B** Comparisons of the posterior distributions of the main effects for all systems from Bayesian linear modeling of the raw performances. For each pair in the matrices, the color gradient reflects the fraction of samples of the forward model color coded on the y-axis that is larger than the forward model color coded on the x-axis. See x-axis labels for color legend.

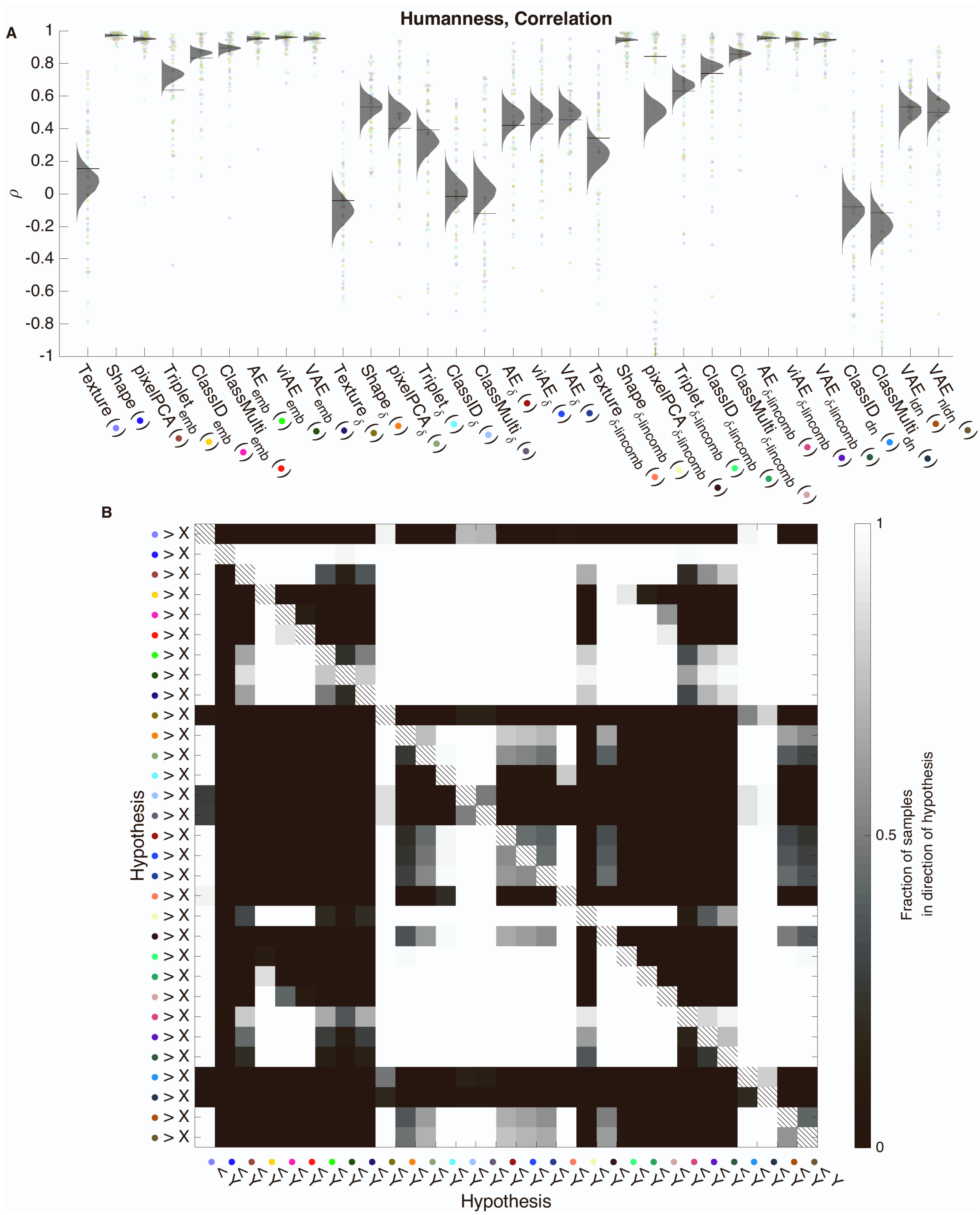

Figure S11: Evaluation of the Pearson correlation between reverse-correlated faces of humans and reverse-correlated faces of models for a larger set of encoding models (related to Figure 4). **A** Pearson correlation (computed with vectors of 3D vertices projected on a single inward-outward dimension) of reverse correlated templates of the models and those of humans. See figure S4 for explanation of the model shorthands. **B** Comparisons of the posterior distributions of the main effects for all systems from Bayesian linear modeling of the raw performances. For each pair in the matrices, the color gradient reflects the fraction of samples of the forward model color coded on the y-axis that is larger than the forward model color coded on the x-axis. See x-axis labels for color legend.

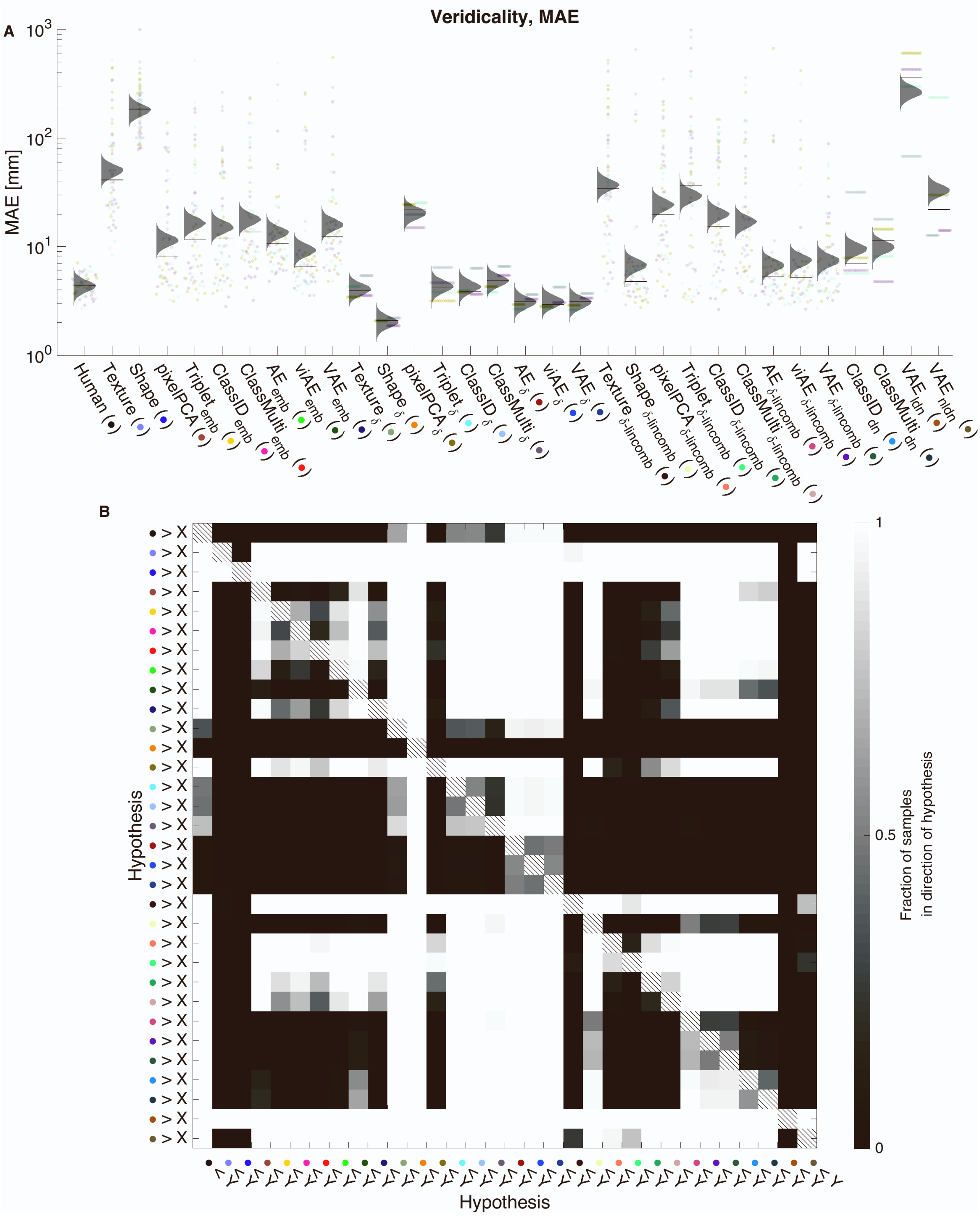

Figure S12: Evaluation of the mean absolute Error between reverse-correlated faces of humans and models and the ground truth face shapes for a larger set of encoding models (related to Figure 4).
A Mean absolute error (MAE, computed as the euclidean distances in 3D space averaged across vertices) of reverse correlated templates of the models and ground truth 3D shape of the target colleagues as captured with a 3D camera array. See figure S4 for explanation of the model shorthands. **B** Comparisons of the posterior distributions of the main effects for all systems from Bayesian linear modeling of the raw performances. For each pair in the matrices, the color gradient reflects the fraction of samples of the forward model color coded on the y-axis that is larger than the forward model color coded on the x-axis. See x-axis labels for color legend.

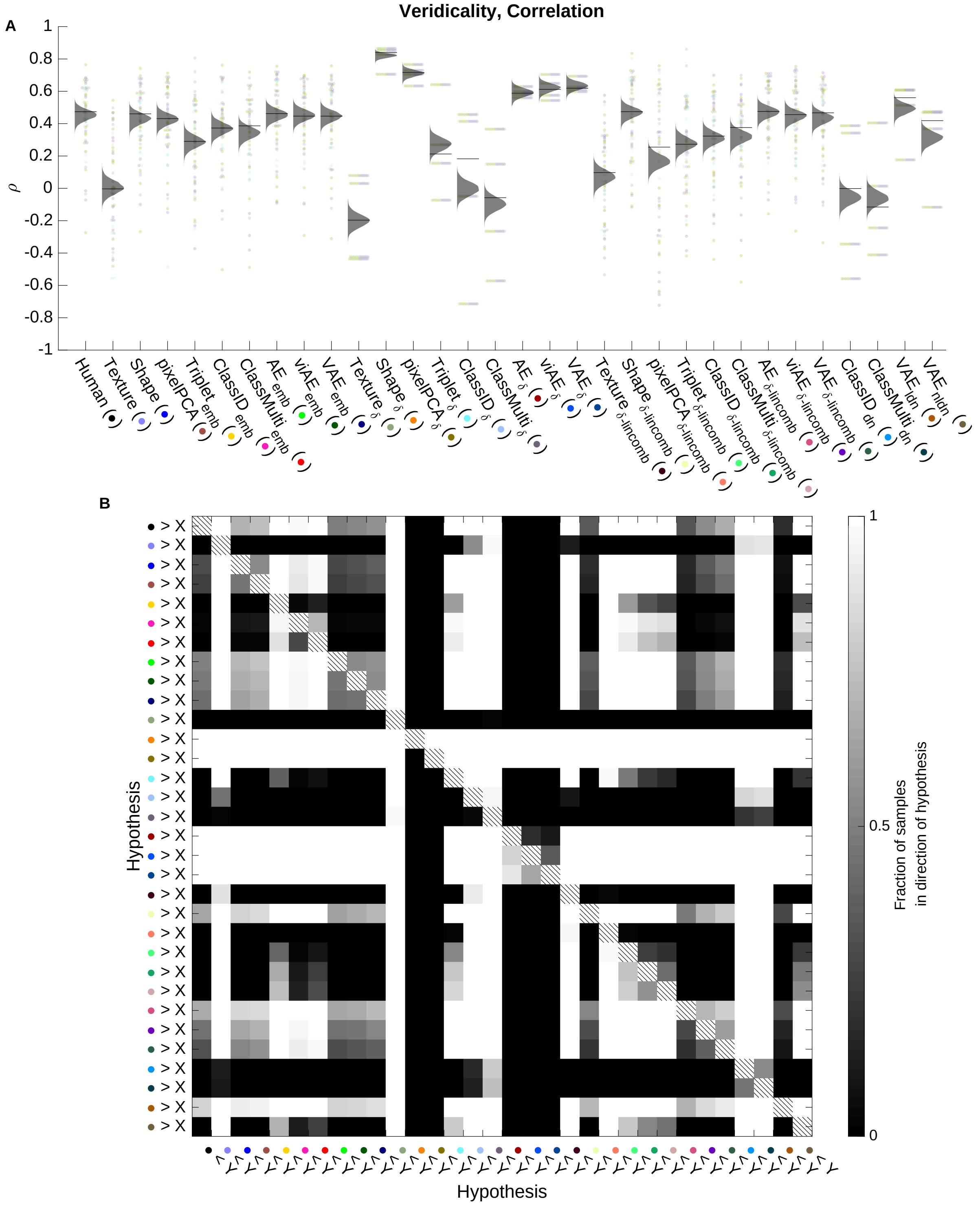

Figure S13: Evaluation of the Pearson correlation between reverse-correlated faces of humans and models and the ground truth face shapes for a larger set of encoding models (related to Figure 4).

A Pearson correlation (computed with vectors of 3D vertices projected on a single inward-outward dimension) of reverse correlated templates of the models and those of humans. See figure S4 for explanation of the model shorthands. **B** Comparisons of the posterior distributions of the main effects for all systems from Bayesian linear modeling of the raw performances. For each pair in the matrices, the color gradient reflects the fraction of samples of the forward model color coded on the y-axis that is larger than the forward model color coded on the x-axis. See x-axis labels for color legend.

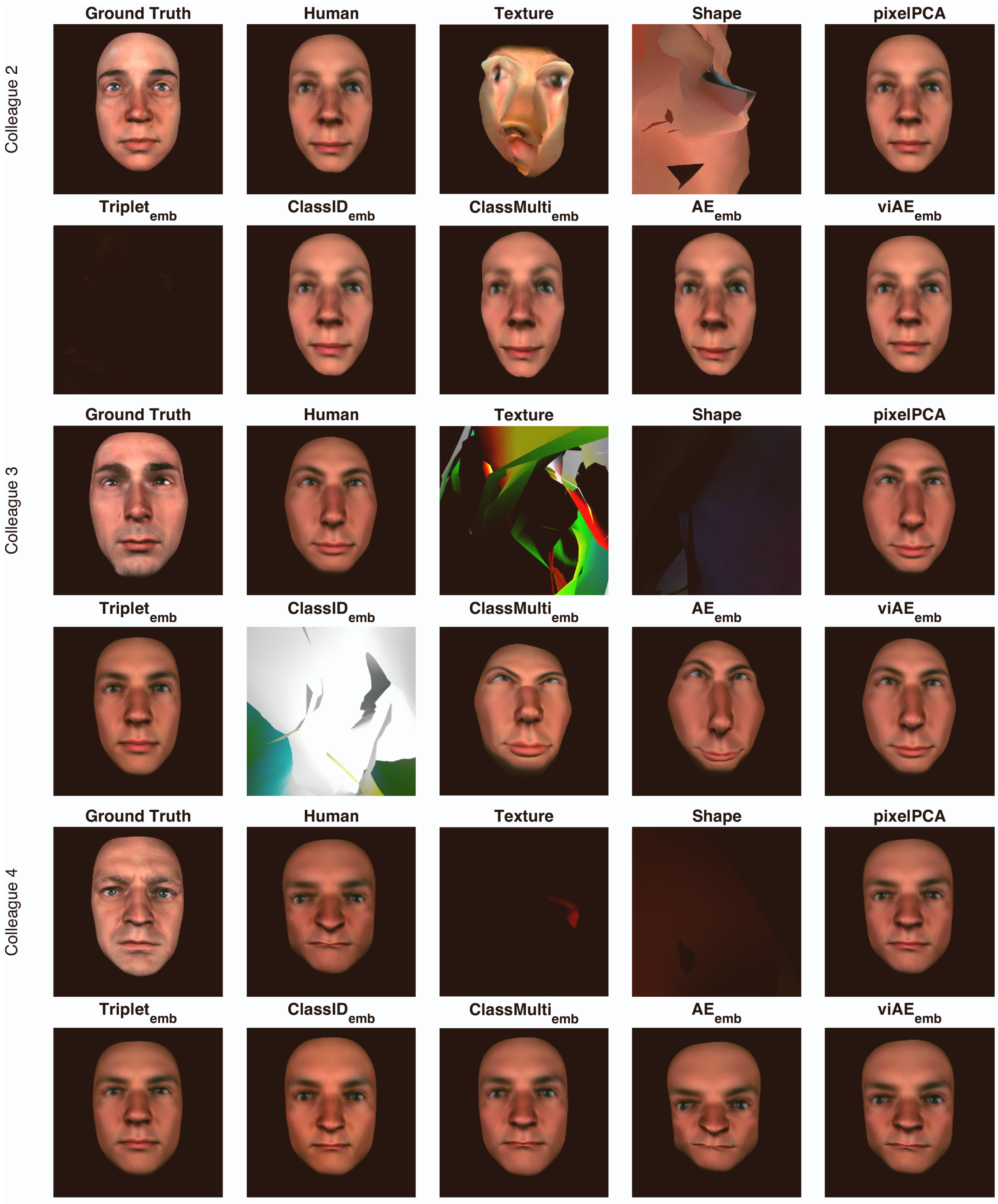

Figure S14: Renderings of reverse-correlated templates of the three remaining colleagues of exemplary participant (related to Figure 4).
Comparison of rendered faces for one exemplary target colleague. Top left panel in each block of two rows shows ground truth face of one target colleague as captured with a 3D camera array. Following panels show reconstructions of the face features from human observed and predicted behavior for one typical participant (i.e. closest to the pooled group medians shown in Figure 4D).

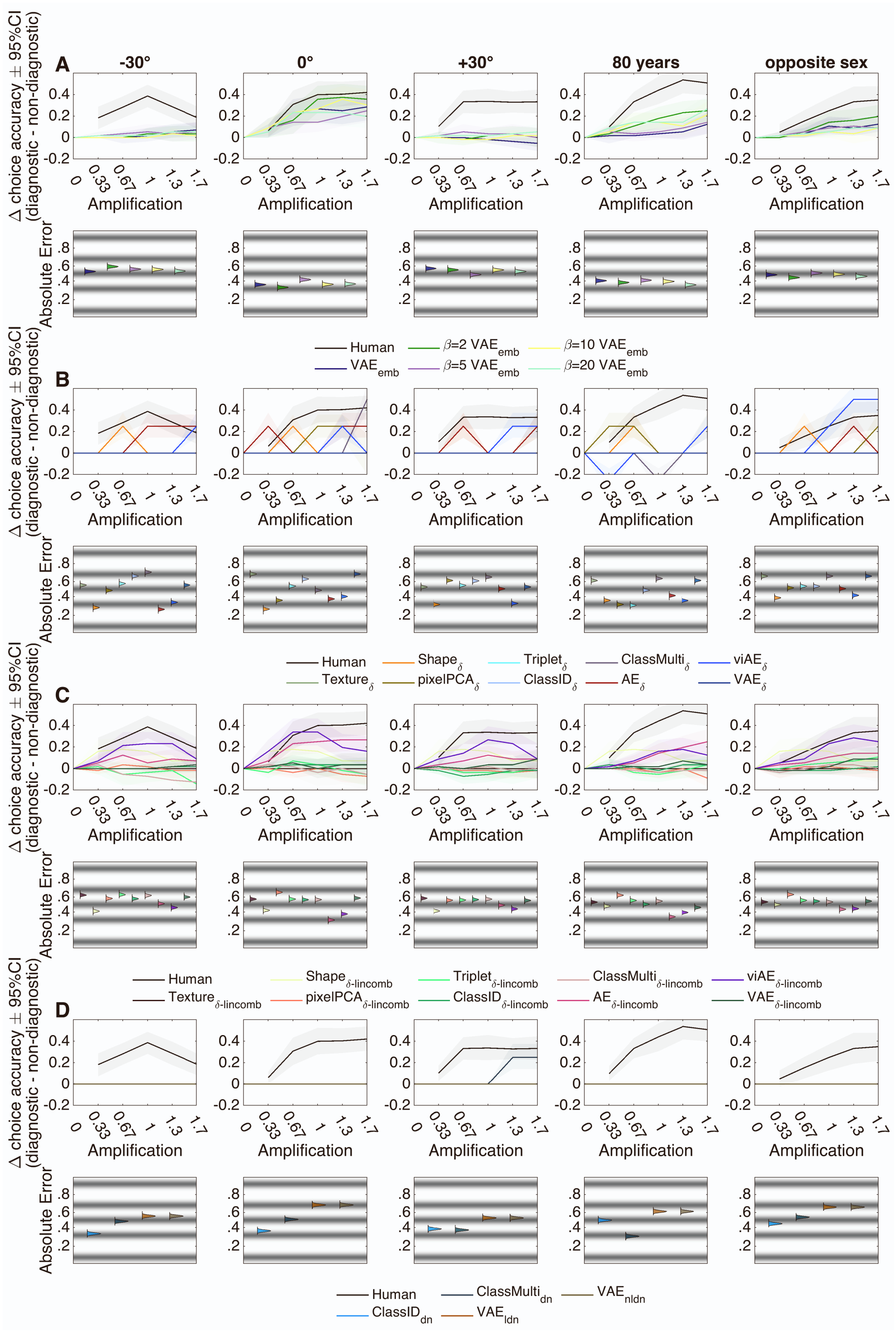

Figure S15: Generalization testing of a larger set of encoding models (related to Figure 5).
A Generalization testing for VAE models with various degrees of regularization. None yield a factorization of the latent spaces that disentangles viewing angle from other factors. Top row shows difference of choice accuracy between the diagnostic and non-diagnostic conditions. Positive values denote a higher accuracy when diagnostic features were amplified. Bottom row shows posterior distributions of main effects of feature spaces when modeling absolute error vs humans with Bayesian linear model. Grey bandings denote density estimates of thresholds separating the five different error values possible (human accuracies are averaged across five ratings of the same item). **B** – **D** show the same as in **A**, but for different forward models. See figure S4 for explanation of the model shorthands.

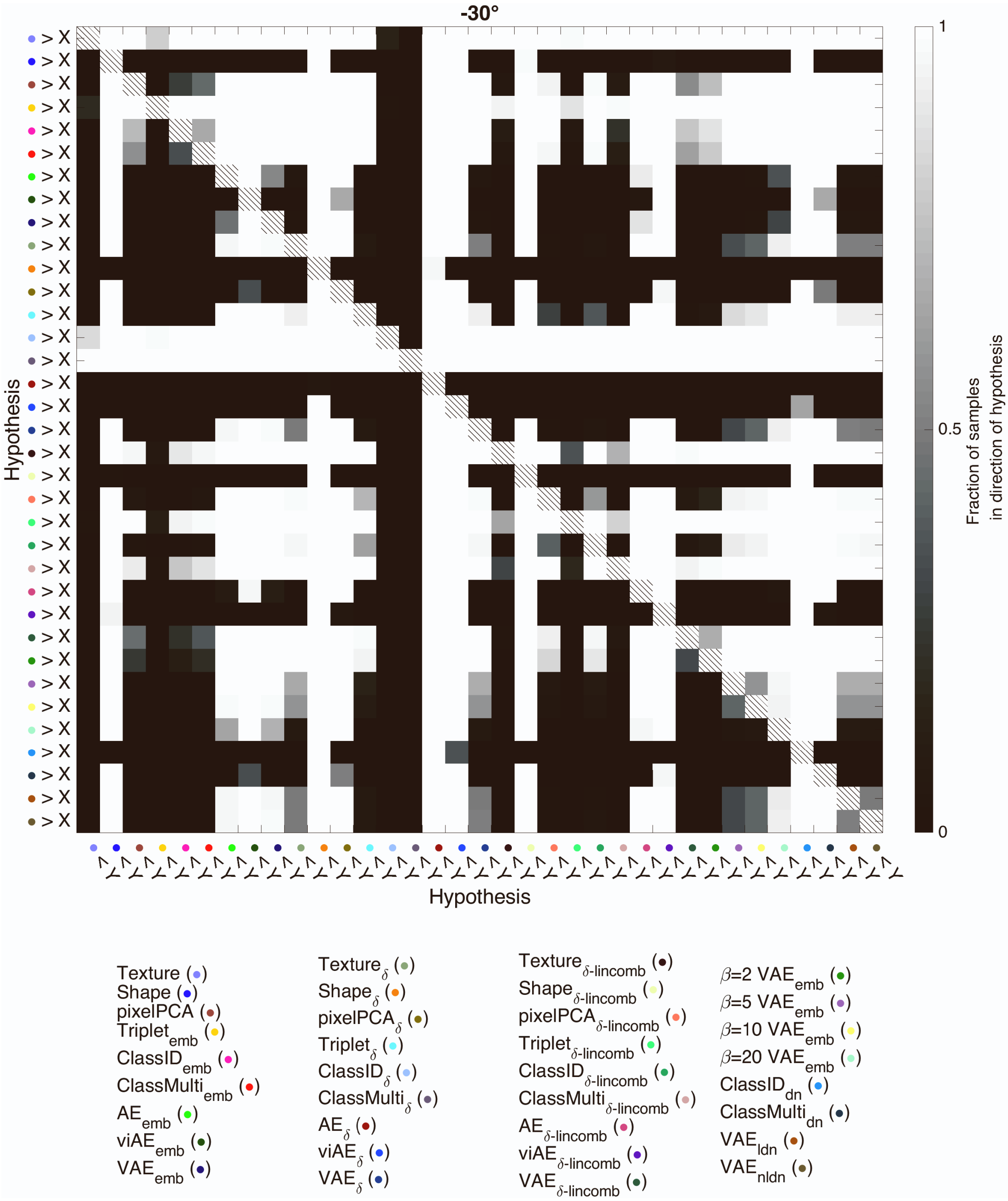

Figure S16: Comparison of posterior distributions for larger set of forward models in -30° viewing angle generalization (related to Figure 5).

Comparison of the posterior distributions of the leftmost column in figure S15. For each pair in the matrices, the color gradient reflects the fraction of samples of the feature space color coded on the y-axis that is larger than the predictor space color coded on the x-axis. See figure S4 for explanation of the model shorthands.

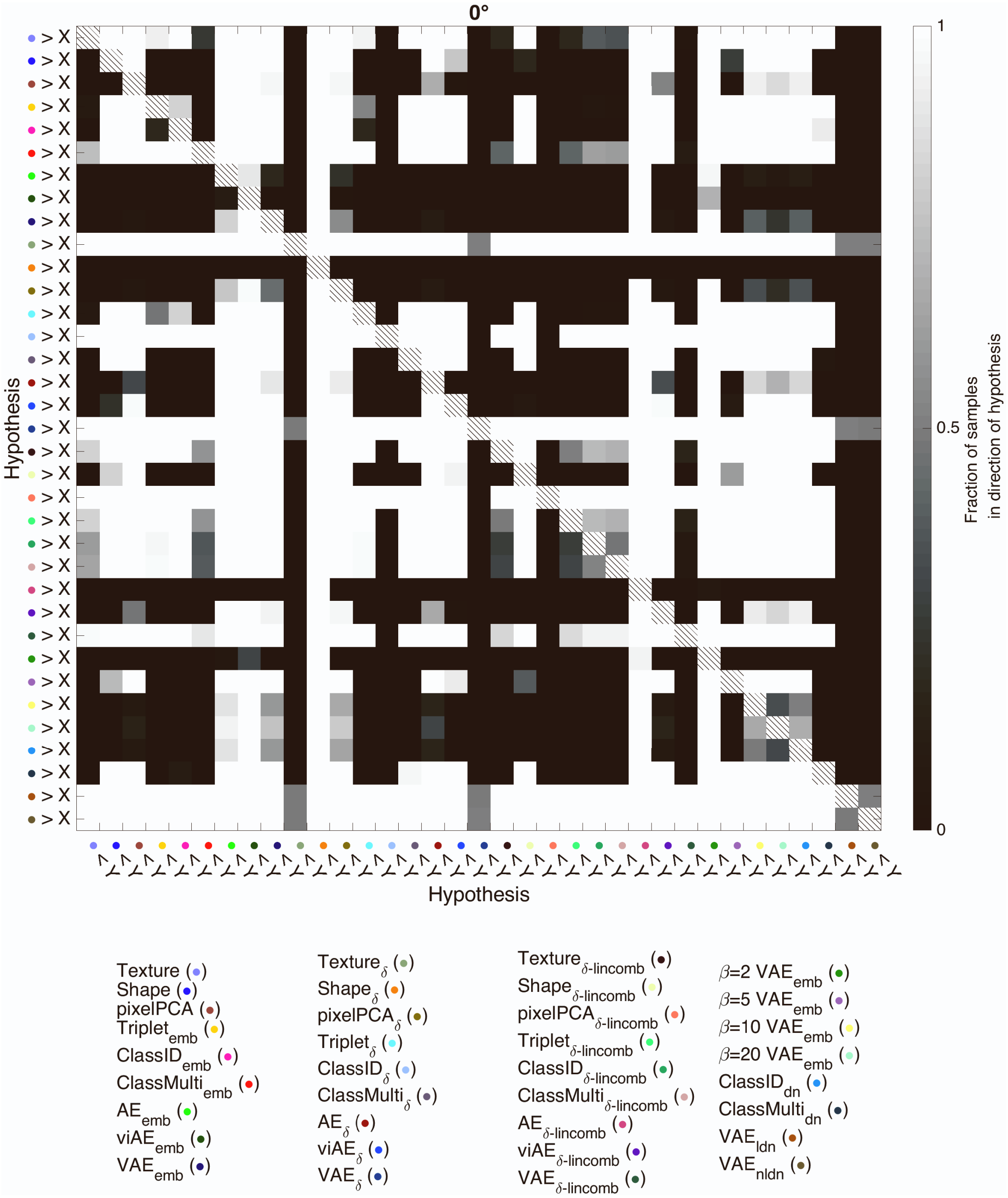

Figure S17: Comparison of posterior distributions for larger set of forward models in 0° viewing angle generalization (related to Figure 5).
Comparison of the posterior distributions of the second column in figure S15. For each pair in the matrices, the color gradient reflects the fraction of samples of the feature space color coded on the y-axis that is larger than the predictor space color coded on the x-axis. See figure S4 for explanation of the model shorthands.

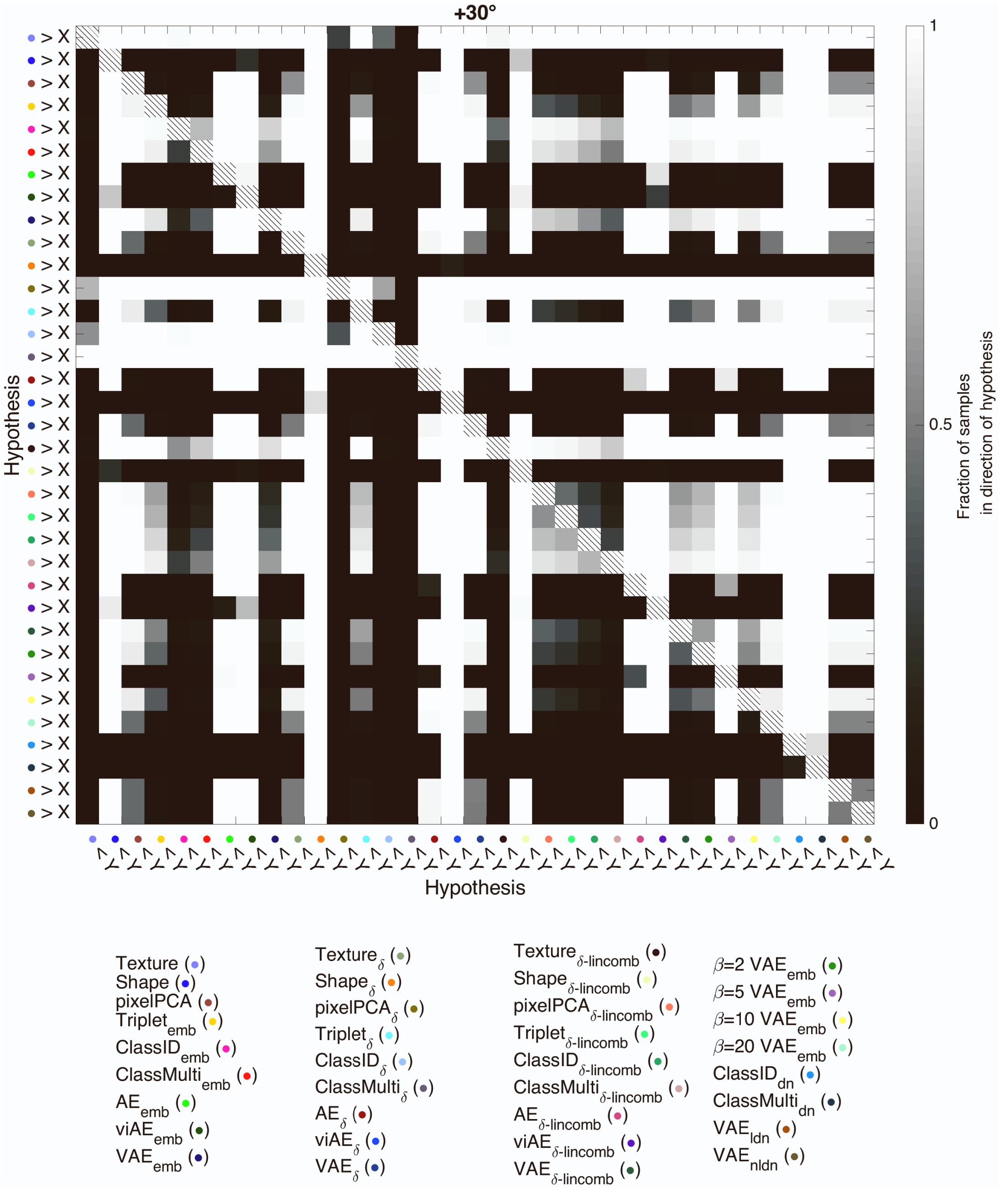

Figure S18: Comparison of posterior distributions for larger set of forward models in +30° viewing angle generalization (related to Figure 5).
Comparison of the posterior distributions of the middle column in figure S15. For each pair in the matrices, the color gradient reflects the fraction of samples of the feature space color coded on the y-axis that is larger than the predictor space color coded on the x-axis. See figure S4 for explanation of the model shorthands.

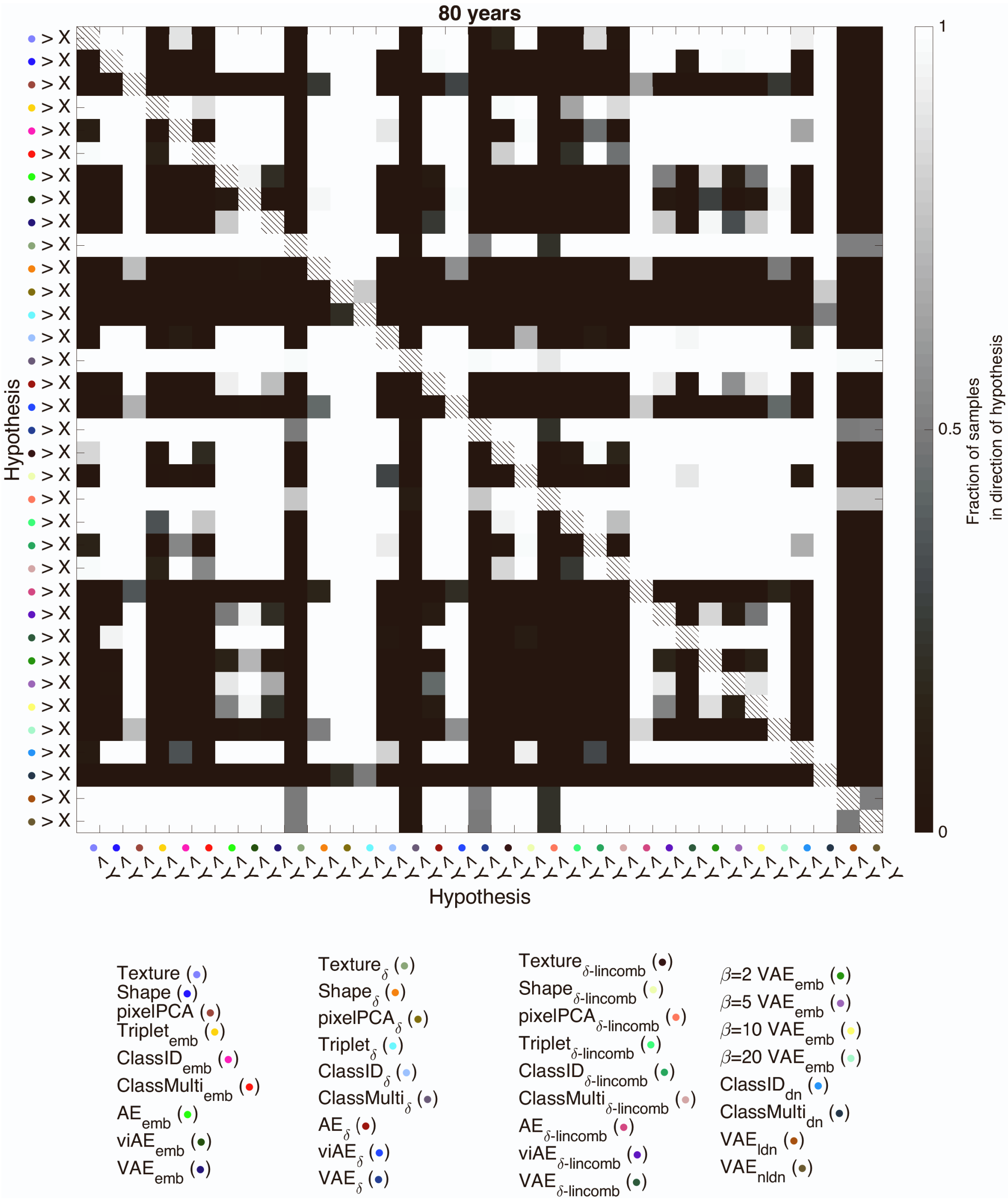

Figure S19: Comparison of posterior distributions for larger set of forward models in 80 years generalization (related to Figure 5).
Comparison of the posterior distributions of the fourth column in figure S15. For each pair in the matrices, the color gradient reflects the fraction of samples of the feature space color coded on the y-axis that is larger than the predictor space color coded on the x-axis. See figure S4 for explanation of the model shorthands.

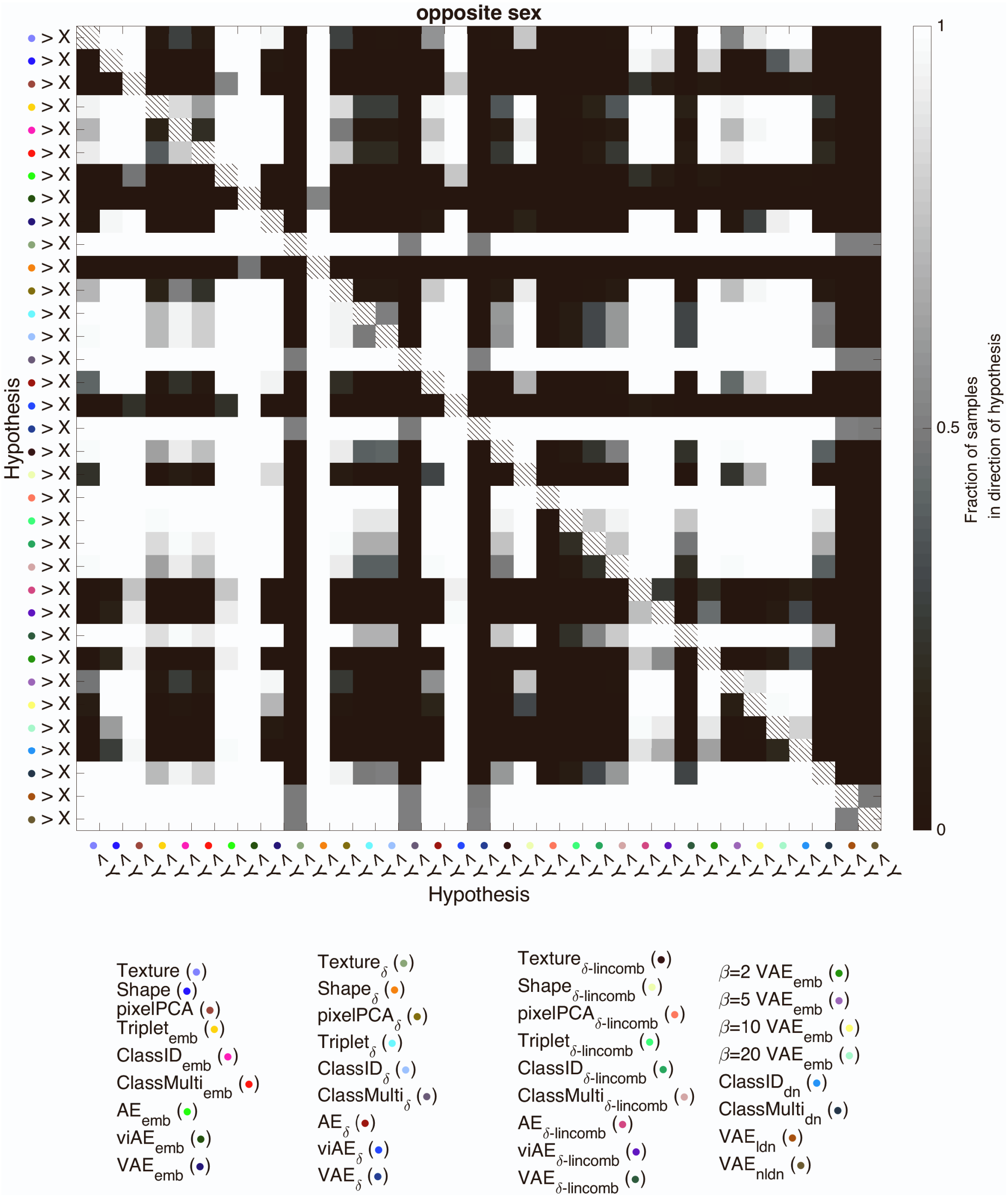

Figure S20: Comparison of posterior distributions for larger set of forward models in opposite sex generalization (related to Figure 5).
Comparison of the posterior distributions of the rightmost column in figure S15. For each pair in the matrices, the color gradient reflects the fraction of samples of the feature space color coded on the y-axis that is larger than the predictor space color coded on the x-axis. See figure S4 for explanation of the model shorthands.

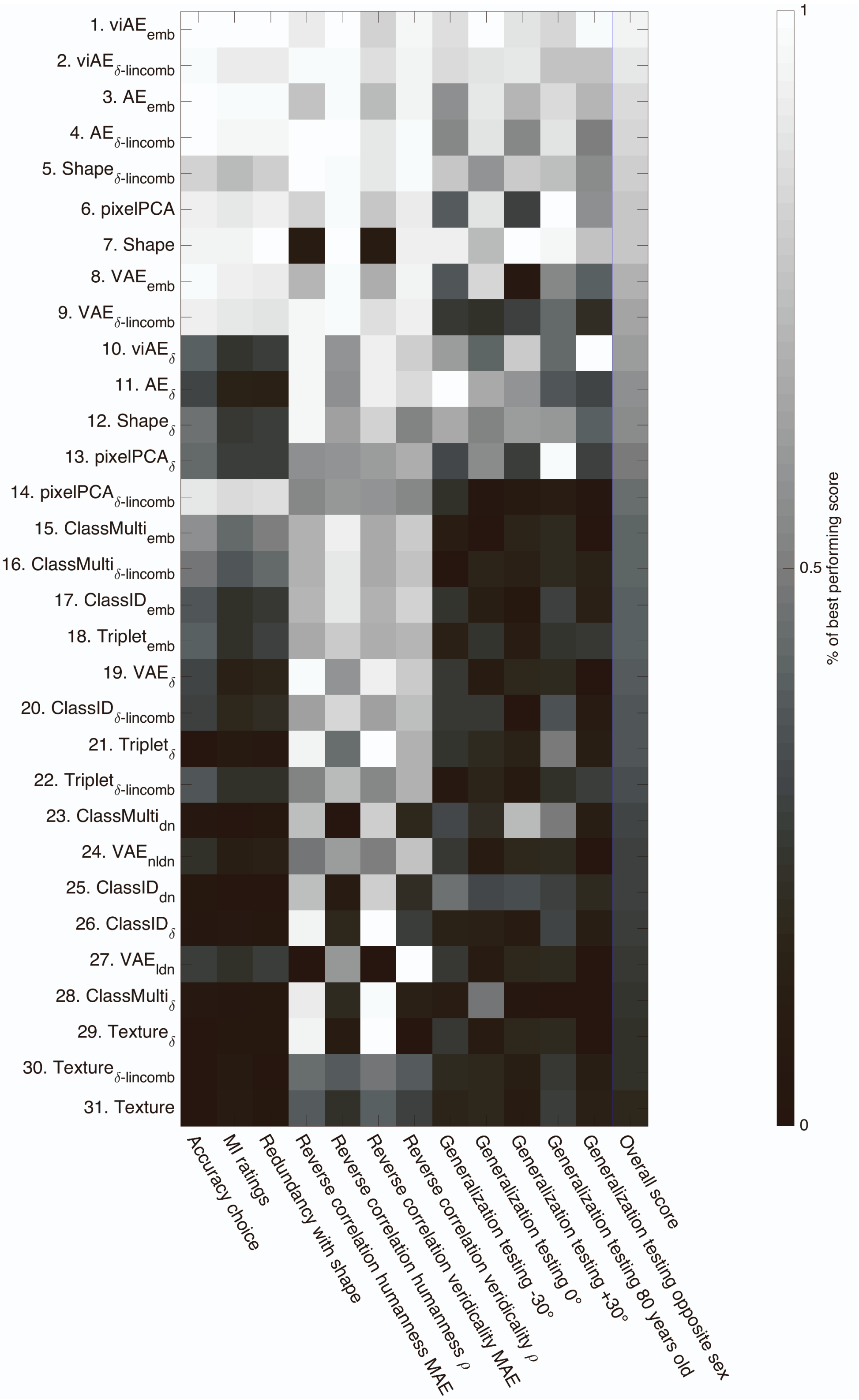

Figure S21: Ranking of extended set of models (related to Figures 3, 5, 6).
We integrated the results of the models in all comparisons except for the re-prediction analysis reported in Figure 4 (which is only applicable to linear combination forward models). Redundancy of the shape model with itself is not computable and was thus manually set to the best possible score. Scores in veridicality of reverse correlation were defined as the absolute difference to the veridicality achieved by humans. Scores in generalization testing (absolute error to human behavior) were additionally penalized for a low delta in accuracy of diagnostic and non-diagnostic stimuli. Performances of models (maxima a posteriori of Bayesian linear models) were normalized within comparisons to range from 0 (worst considered model) to 1 (best considered model). Scores were summed across comparisons and divided by the number of comparisons for the overall score. See figure S4 for explanation of the model shorthands.

Supplemental References

1. Kingma DP, Welling M. Auto-Encoding Variational Bayes. arXiv:13126114 [cs, stat]. 2014 May;ArXiv: 1312.6114. Available from: http://arxiv.org/abs/1312.6114.

2. Higgins I, Matthey L, Pal A, Burgess C, Glorot X, Botvinick M, et al. beta-VAE: Learning Basic Visual Concepts with a Constrained Variational Framework. ICLR. 2016 Nov;Available from: https://openreview.net/forum?id=Sy2fzU9gl.
